# Enantioselective total synthesis of (–)-colchicine, (+)-demecolcinone and metacolchicine: determination of the absolute configurations of the latter two alkaloids†
†Electronic supplementary information (ESI) available. CCDC 1547674 and 1524362. For ESI and crystallographic data in CIF or other electronic format see DOI: 10.1039/c7sc01341h



**DOI:** 10.1039/c7sc01341h

**Published:** 2017-05-05

**Authors:** Bo Chen, Xin Liu, Ya-Jian Hu, Dong-Mei Zhang, Lijuan Deng, Jieyu Lu, Long Min, Wen-Cai Ye, Chuang-Chuang Li

**Affiliations:** a Department of Chemistry , South University of Science and Technology of China , Shenzhen 518055 , China . Email: ccli@sustc.edu.cn; b College of Pharmacy , Jinan University , Guangzhou 510632 , China; c Institute of Chinese Medical Sciences , University of Macau , Macao , China

## Abstract

Here, we describe a concise, enantioselective, and scalable synthesis of (–)-colchicine (9.2% overall yield, >99% ee). Moreover, we have also achieved the first syntheses of (+)-demecolcinone and metacolchicine, and determined their absolute configurations. The challenging tricyclic 6-7-7 core of colchicinoids was efficiently introduced using an intramolecular oxidopyrylium-mediated [5 + 2] cycloaddition reaction. Notably, the synthesized colchicinoid **23** exhibited potent inhibitory activity toward the cell growth of human cancer cell lines (IC_50_ = ∼3.0 nM), and greater inhibitory activity towards microtubule assembly than colchicine, making it a promising lead in the search for novel anticancer agents.

## Abstract 2

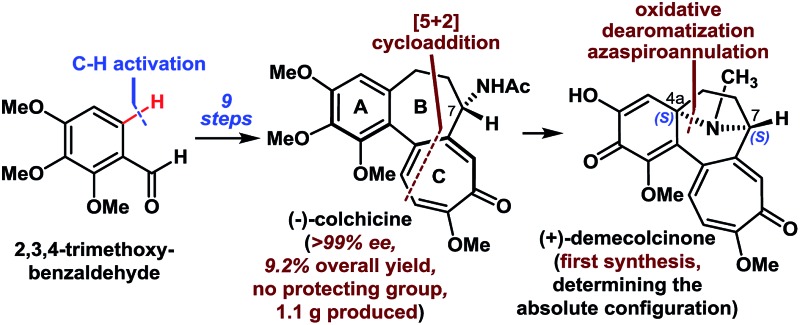
A highly concise, enantioselective synthesis of (–)-colchicine, the first syntheses of (+)-demecolcinone and metacolchicine, was reported.

## Introduction

**Fig. 1:**
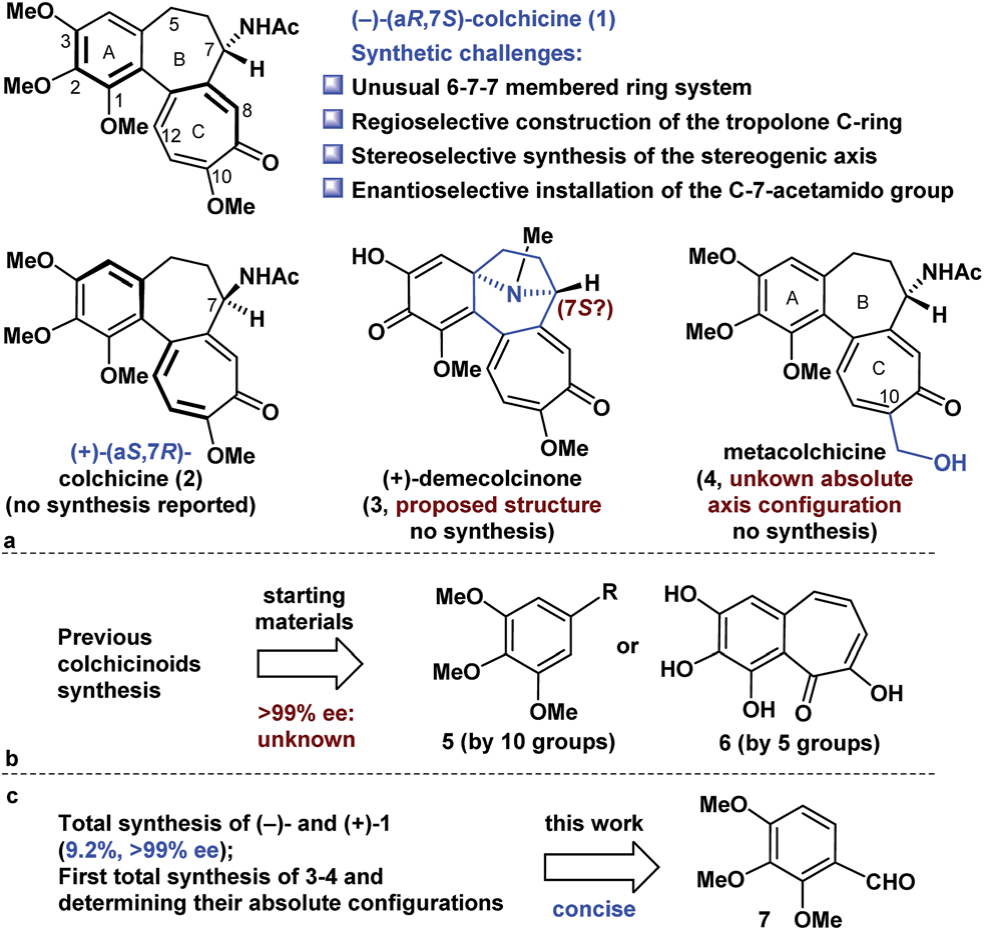
Selected colchicinoids and a summary of their syntheses.

Colchicine (**1**), an alkaloid natural product, was the first tubulin-destabilizing agent to be reported in the literature (Fig. 1a). The effects of this compound against a variety of indications have been investigated owing to its remarkable antimitotic activity. Furthermore, colchicine (**1**) has been used to treat several diseases, including acute gout, familial Mediterranean fever, and chronic myelocytic leukemia.^1^ Colchicine (**1**) has also been used as a neurotoxin in animal models of Alzheimer's disease and epilepsy.^2^ The development of novel colchicinoids with enhanced antitumor properties continues to pose a significant challenge to drug discovery scientists.^3^ From a structural perspective,^4^ the unusual 6-7-7-membered ring system of colchicinoids **1–4**, as well as the stereocenter at C-7 and the a*R*-configured stereogenic axis defined by the pivot bond joining the A and C rings, represents a formidable synthetic challenge. The regioselective construction of the highly oxidized tropolone C ring and the stereoselective installation of the C-7-acetamido group undoubtedly represent two of the more challenging features of any prospective colchicinoid synthesis.^5^ It is noteworthy that (+)-demecolcinone (**3**), which was the first naturally occurring dextrorotatory colchicinoid to be reported, contains a constrained azabicyclo[3.2.1]octane (tropane) framework that is unprecedented in nature.^6^ Nevertheless, the relative configuration of naturally occurring (+)-demecolcinone (**3**) was proposed only based on the energy-minimized representation of the molecule and the postulated biosynthetic pathway, owing to the lack of direct and conclusive evidence. The absolute configuration of (+)-demecolcinone (**3**) has not been determined through X-ray crystallographic analysis or the exciton chirality circular dichroism method. Furthermore, metacolchicine (**4**) is the first example of a naturally occurring colchicinoid bearing an additional carbon unit at its C-10 position.^7^ However, the absolute configuration of the unique stereogenic axis defined by the pivot bond joining the A and C rings of metacolchicine (**4**) has not previously been addressed, and there have been no reports on the biological activity of this new colchicinoid.

The fascinating structural motifs and promising pharmacological properties of the colchicinoids have attracted considerable interest from the synthetic chemistry community over the past 60 years, and this has culminated in an large number of interesting reports.^5a,8,9^ However, most of the existing routes to this class of compounds are heavily reliant on the use of symmetric C-5-substituted pyrogallol trimethyl ethers (**5**) and purpurogallin (**6**) as starting materials (Fig. 1b).^5*a*^ All previous asymmetric installations of the C-7-amino group are limited to the following procedure: asymmetric reduction of the ketone to the alcohol, subsequent conversion of the alcohol to the azide, and reduction of the azide to the amino group.^8b,8c,8d^ The highly enantioselective (≥99% ee) synthesis of colchicine (**1**) also remains a great challenge because of the partial racemization of relative compounds at the C-7 proton.^8*b*^ Notably, there have been no reports in the literature to date pertaining to the total synthesis of compounds **2–4**. Herein, we describe a highly concise and enantioselective synthesis of (–)-colchicine and (+)-colchicine (>99% ee). We also describe the first reported syntheses of (+)-demecolcinone (**3**) and metacolchicine (**4**), including determination of their absolute configurations, using 2,3,4-trimethoxybenzaldehyde (**7**) as a new starting material.

## Results and discussion

### Retrosynthetic analysis of colchicinoids

**Fig. 2:**
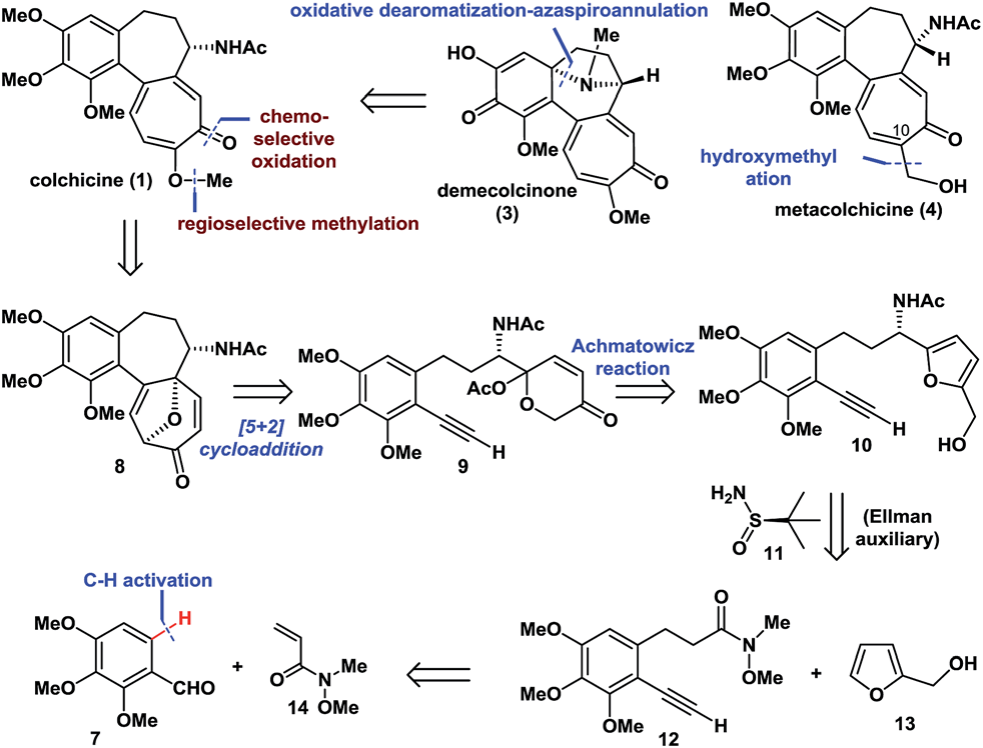
Retrosynthetic analysis of colchicinoids **1**, **3** and **4**.

Retrosynthetically (Fig. 2), demecolcinone (**3**) could be prepared from colchicine (**1**) in a biomimetic manner through an intramolecular oxidative dearomatization–azaspiroannulation reaction.^10^ Furthermore, metacolchicine (**4**) could be derived from colchicine (**1**) by regioselective hydroxymethylation at its C-10 position. It was envisioned that colchicine (**1**) could be generated from **8** through a chemoselective oxidation, followed by regioselective formation of the tropolone C-ring. Inspired by previous work,^11^ tricyclic **8** could itself be synthesized from **9** in an intramolecular oxidopyrylium-mediated [5 + 2] cycloaddition reaction.^12,13^ Compound **9** can be derived from **10** through the Achmatowicz reaction,^14^ and compound **10** could be synthesized enantioselectively by reaction of amide **12** with furfuryl alcohol (**13**) followed by the diastereoselective reductive amination of the resulting ketone with Ellman auxiliary **11**.^15^ In this sense, it was envisioned that **11** could be used as a chiral directing group to provide the required amino group as a single enantiomer. Amide **12** could be synthesized by reaction of commercially available and inexpensive compound **7** with *N*-methoxy-*N*-methylacrylamide (**14**) *via* a transition-metal-catalysed C–H functionalization reaction^16^ using the aldehyde as a directing group, followed by a Seyferth–Gilbert homologation.^17^

### Enantioselective total synthesis of (–)- and (+)-colchicine

**Scheme 1:**
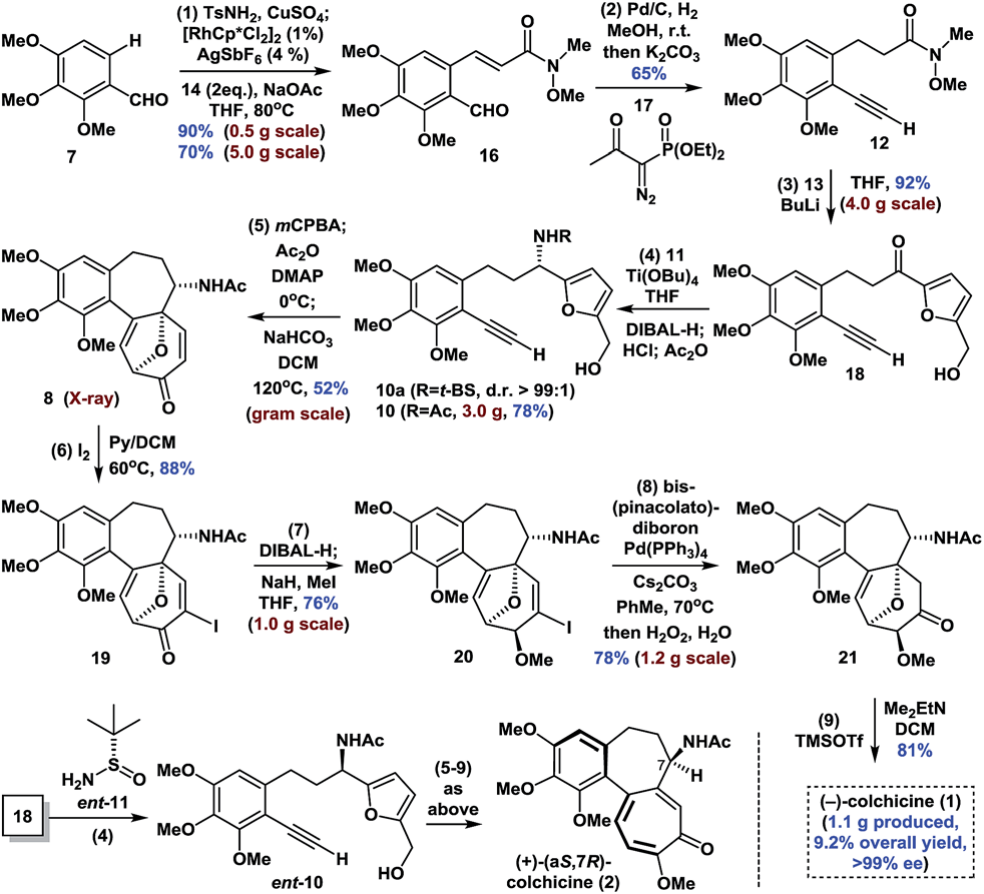
Enantioselective synthesis of (–)-colchicine and (+)-colchicine.

The synthesis began with the transition-metal-catalyzed C–H bond functionalization of **7** with **14** (Scheme 1). Inspired by Li's seminal work,^18^ we applied the strategy to compound **7**. Pleasingly, after optimization, we successfully generated the *N*-sulfonyl imine *in situ* by reaction of **7** with TsNH_2_ (**15**) in the presence of anhydrous CuSO_4_ in THF. Furthermore, subsequent treatment of this imine with [RhCp*Cl_2_]_2_ (1 mol%), AgSbF_6_ (4 mol%), NaOAc (2.0 equiv.), and **14** (2.0 equiv.) at 80 °C afforded *ortho*-olefinated benzaldehyde **16** in good yield (90% on a 0.5 g scale; 70% on a 5.0 g scale). This modified catalytic C–H bond activation involved a transient directing group.^19^

Moving forward, the subsequent selective hydrogenation of **16** (Pd/C, H_2_), followed by the Seyferth–Gilbert homologation of the aldehyde moiety in the presence of **17** gave **12** in one pot in 65% yield. 1,2-Addition of the organolithium reagent prepared by treatment of furfuryl alcohol (**13**) with BuLi to **12** provided ketone **18** in 92% yield (4.0 g scale). Subsequent condensation of ketone **18** with Ellman auxiliary **11** using Ti(OBu)_4_ (2.0 equiv.) in THF, followed by reduction of the resulting *tert*-butanesulfinyl (*t*-BS) ketimine with DIBAL-H afforded **10a** (R = *t*-BS) with very high diastereoselectivity. Treatment of **10a** with HCl and methanol in the same pot resulted in clean cleavage of the sulfinyl group to give the corresponding amine, which was acetylated *in situ* in the presence of NaHCO_3_ to give **10** (R = Ac, >99% ee) in 78% yield (one-pot, 3.0 g scale, see the ESI for details†). Oxidative rearrangement of **10** using 3-chloroperoxybenzoic acid (*m*CPBA), followed by acetylation of the anomeric hydroxyl and intramolecular [5 + 2] cycloaddition of acetoxypyranone **9** under our optimized one-pot reaction conditions, gave tricyclic 6-7-7 core-containing **8** in 52% yield (1.0 g scale), which was confirmed by X-ray crystallography.

Next, we continued with our proposed total synthesis of colchicine (**1**) from **8**. After extensive experimentation, we found that treatment of **8** with iodine in a mixture of pyridine and DCM, afforded α-iodoenone **19** in 88% yield. Reduction of the ketone group in **19** followed by *in situ* chemoselective methylation of the resulting alcohol afforded **20** in 76% yield (1.0 g scale). A palladium-catalyzed cross-coupling reaction of bis(pinacolato)diboron with **20** followed by oxidation under mild conditions (H_2_O_2_/H_2_O) gave ketone **21** with 78% yield (1.2 g scale). Finally, double elimination of the oxa-bridge in **21** using a slightly modified version of Cha's procedure^8*c*^ in the presence of TMSOTf and Me_2_EtN proceeded smoothly to complete our total synthesis of (–)-**1** in >99% ee. We also achieved the first synthesis of (+)-(a*S*,7*R*)-colchicine (**2**, *ent*-**1**), using Ellman auxiliary *ent*-**11** for condensation with ketone **18**, according to a similar sequence to that shown in Scheme 1. Notably, this route provided facile access to a total of 1.1 g of (–)-**1**, thereby highlighting the robust nature of this chemistry.

### Enantioselective synthesis of (+)-demecolcinone (**3**) and metacolchicine (**4**)

**Scheme 2:**
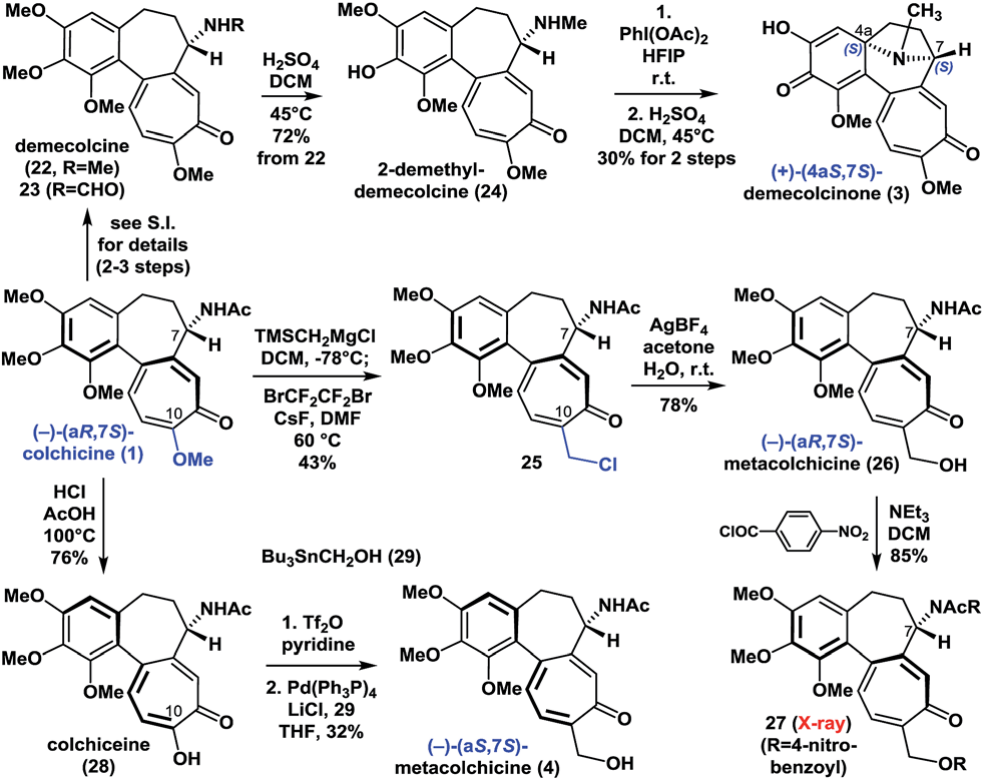
Enantioselective synthesis of (+)-demecolcinone (**3**) and metacolchicine (**4**), and determination of their absolute configurations.

With (–)-colchicine (**1**) in hand, we proceeded to investigate our proposed syntheses of the remaining colchicinoids (Scheme 2). Demecolcine (**22**, R = Me) and **23** (R = CHO) were rapidly prepared from colchicine (**1**) through a series of slightly modified procedures from the literature (see ESI†). The application of a modified version of Brossi's procedure^20^ (H_2_SO_4_ in DCM at 45 °C) to **22** gave 2-demethyldemecolcine (**24**) in 72% yield. It is worth noting that compound **22** exhibited much better chemoselectivity towards *O*-demethylation than did colchicine (**1**) and **23** under the same conditions.

Our initial efforts to construct the 8-azabicyclo[3.2.1]octane structure in demecolcinone (**3**) involved the intramolecular oxidative dearomatization–azaspiroannulation reaction of **22** using a variety of hypervalent iodine sources^21^ in several different solvents. Unfortunately, none of these reactions afforded any of the desired products. However, after extensive experimentation, we found that treatment of **24** with PhI(OAc)_2_ in the highly polar solvent hexafluoroisopropanol at 25 °C followed by demethylation completed our synthesis of (+)-demecolcinone (**3**). The ^1^H and ^13^C NMR spectra of synthetic (+)-demecolcinone (**3**), as well as its optical rotation, were identical to those of the natural product. Thus, the absolute configuration of naturally occurring (+)-**3**, which was the first naturally occurring dextrorotatory colchicinoid to be reported in the literature,^6^ was unambiguously established to be 4a*S*,7*S* based on our total synthesis. It is noteworthy that this study represents the first reported application of an oxidative dearomatization–azaspiroannulation strategy to the synthesis of a medicinally significant tropane structure.^22^

We then continued to investigate the synthesis of metacolchicine (**4**), although we believed it would be challenging to regioselectively install the hydroxymethyl group at the C-10 position of colchicine (**1**). After a long period of exploration, we pleasingly found that treatment of colchicine (**1**) with (trimethylsilyl)methylmagnesium chloride, followed by halogenation in the presence of CsF and BrCF_2_CF_2_Br in the same pot, gave **25**. This novel process involved a series of sequential reactions, including the regioselective 1,8-conjugate addition of (trimethylsilyl)methylmagnesium chloride to colchicine (**1**) at C10, elimination of the C10-methoxyl group, and desilylation, bromination, and chlorination (see ESI†). It is noteworthy that the chlorine atom in **25** is derived from the (trimethylsilyl)methylmagnesium chloride. Later, chloride **25** was smoothly hydrolyzed with AgBF_4_ in acetone and H_2_O to afford (–)-(a*R*,7*S*)-metacolchicine (**26**) in 78% yield. The structure of **26** was determined by 2D-NMR spectroscopy and confirmed by X-ray crystallographic analysis of its derivative **27**. Surprisingly, however, the ^1^H and ^13^C NMR spectra of **26** differed from those of the natural product of metacolchicine (**4**).^7^ The absolute configuration of naturally occurring metacolchicine (**4**) has only two possibilities: a*R*,7*S* or a*S*,7*S*. Thus, the absolute configuration of naturally occurring metacolchicine (**4**) was determined to be a*S*,7*S* according to our synthesis. To the best of our knowledge, naturally occurring (–)-(a*S*,7*S*)-metacolchicine (**4**) is the first compound to be identified that has a different absolute configuration from that of other colchicine-type natural products.

Finally, the C10-methoxyl group of colchicine (**1**) was demethylated with high regioselectivity to give colchiceine (**28**) in good yield. Triflation of **28**, followed by a Stille–Migita coupling reaction (Pd(PPh_3_)_4_, Bu_3_SnCH_2_OH (**29**)) furnished metacolchicine (**4**) in 32% overall yield. The ^1^H and ^13^C NMR spectra and the optical rotation (synthetic: [*α*]_D_^20^ = –167 (*c* = 1.0, CHCl_3_); natural: [*α*]_D_^21^ = –160 (*c* = 0.39, CHCl_3_)) of newly synthesized metacolchicine (**4**) were identical to those of the natural product. In this process (from **1** to **4**) clean inversion of axial stereochemistry was found, indicating that the inversion probably occurred during the conversion of **1** to **28** at 100 °C in the presence of acid. This approach could also be applied to the syntheses of structurally diverse analogues of colchicinoids bearing different groups at their C-10 positions and with different stereogenic axes. This would make it possible to carry out structure–activity relationship (SAR) studies on these compounds.

### Cell growth inhibitory activities of colchicinoids and tubule-targeting activities of **23**

The cell growth inhibitory activities of compounds **1–4** and **22–28** were determined in three human cancer cell lines, namely lung adenocarcinoma (A549), breast carcinoma (MDA-MB-231), and colon adenocarcinoma (LoVo) cells (Table 1, see ESI for details†). Of the compounds tested in this study, compound **23** displayed the most potent inhibitory effects, with IC_50_ values of 2.8, 3.2, and 3.5 nM against A549, MDA-MB-231, and LoVo cells, respectively.

**Table 1:** Cell growth inhibitory effects of selected synthesized colchicinoids

Compounds	IC_50_ ^*a*^ (×±SD) μM		
	A549	MDA-MB-231	LoVo
**1**	0.0710 ± 0.0110	0.0332 ± 0.0310	0.0087 ± 0.0023
**2**	>50	>50	>50
**3**	>50	>50	>50
**4**	0.4481 ± 0.1190	0.5752 ± 0.4881	0.4030 ± 0.0647
**22**	0.0276 ± 0.0106	0.0310 ± 0.0047	0.0346 ± 0.0032
**23**	0.0028 ± 0.0009	0.0032 ± 0.0003	0.0035 ± 0.0006

^*a*^IC_50_ values are expressed as the mean values ± S.D. from three independent experiments.

**Fig. 3:**
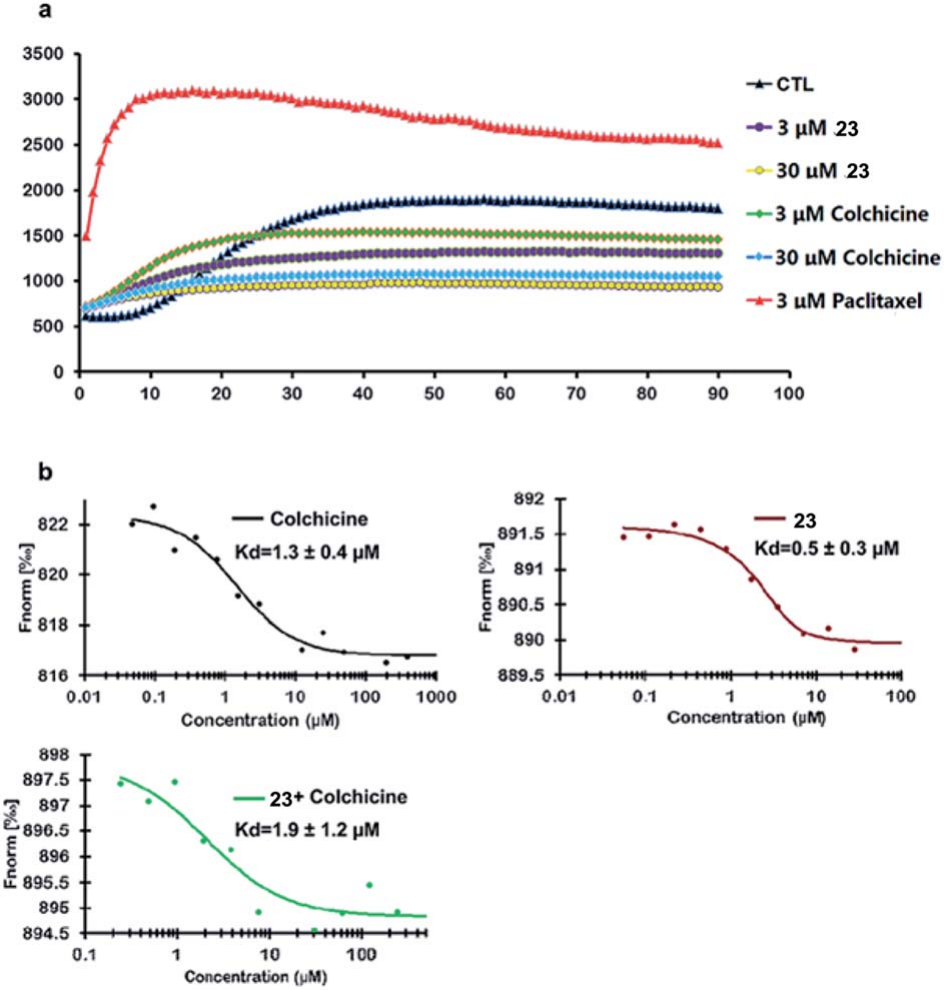
Inhibition of the polymerization of tubulin by **23** and direct binding of **23** to tubulin *in vitro*.

Compound **23** was selected as a representative example to determine whether the synthesized colchicinoids acted as microtubule-targeting agents. As shown in Fig. 3a (see ESI†), **23** inhibited the polymerization of tubulin in a dose-dependent manner, thereby exhibiting similar behavior to that of colchicine (**1**). Microscale thermophoresis (MST) was used with purified recombinant tubulin to further confirm that **23** directly interfered with the assembly of tubulin monomers into microtubules. Colchicine (**1**) was also analyzed by MST as a positive control. The resulting MST measurements gave dissociation constants (*K*_d_) of 0.5 ± 0.3 and 1.3 ± 0.4 μM for **23** and colchicine (**1**), respectively (Fig. 3b). Furthermore, pre-treatment of recombinant tubulin with **23** did not lead to any discernible difference in the binding affinity of colchicine (**1**) (1.9 ± 1.2 μM) for tubulin, which indicates that **23** binds to a different binding site on tubulin than does colchicine (**1**). It is noteworthy that the inhibitory activity of **23** towards the polymerization of tubulin and the binding affinity of **23** to tubulin were both more potent than those of colchicine (**1**) *in vitro*.

**Fig. 4:**
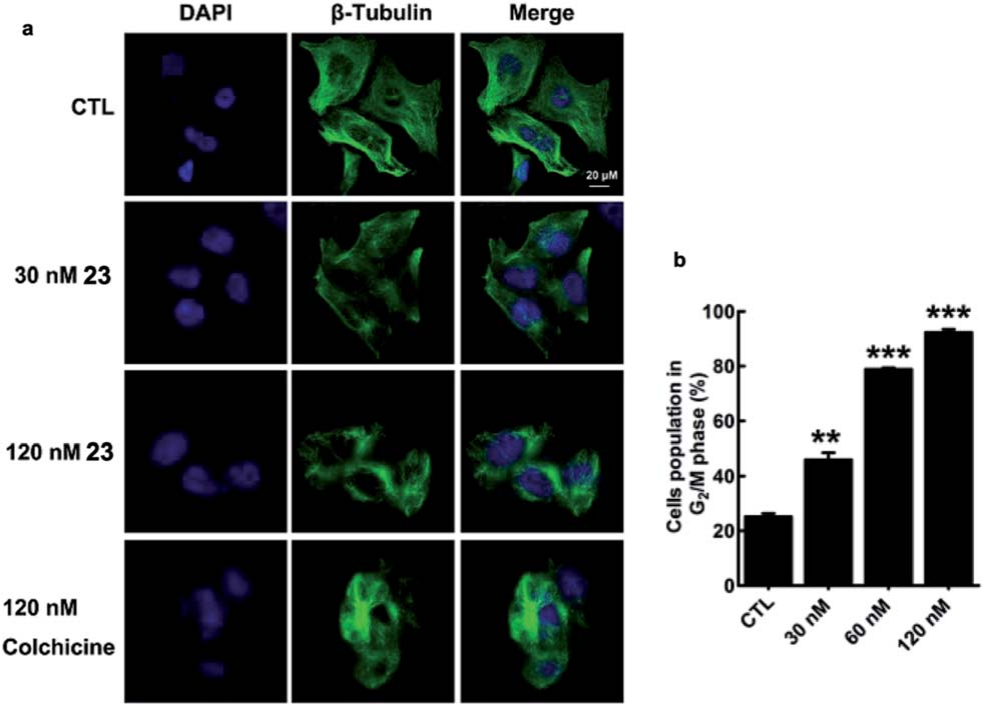
Disruption of the microtubule structure and induction of G_2_/M cell cycle arrest in MDA-MB-231 cells by **23**.

Microtubule-targeting drugs damage the microtubule structure of cells and induce cell cycle arrest in the G_2_/M phase by disrupting the formation of the mitotic spindle required for mitosis.^1*d*^ We observed morphological changes in the microtubule structure of MDA-MB-231 cells after they had been exposed to **23** or colchicine (**1**) for 12 h. Treatment of MDA-MB-231 cells with **23** resulted in considerable disruption to their microtubule network with short microtubules. Similar effects were also observed for colchicine (**1**) (Fig. 4a). As shown in Fig. 4b, treatment of MDA-MB-231 cells with **23** led to a considerable dose-dependent increase in the number of cells in the G_2_/M phase of the cell cycle, with values increasing from 25.19 ± 1.97% (CTL) to 46.03 ± 4.42% (30 nM), 78.85 ± 0.94% (60 nM) and 92.32 ± 2.08% (120 nM). These results therefore indicate that the activity of **23** stems from its effect on the depolymerization of the microtubules.

## Conclusions

In summary, we have developed a concise and highly enantioselective synthesis of colchicine (**1**) (1.1 g, >99% ee) in nine steps^23^ and 9.2% overall yield, without the need for protecting groups.^24^ An intramolecular oxidopyrylium-mediated [5 + 2] cycloaddition reaction was used as a key step in this synthesis for efficient formation of the challenging tricyclic 6-7-7 core. In addition to achieving the most enantioselective total synthesis of colchicine (**1**) reported to date, we have also succeeded in describing the first reported asymmetric syntheses of (+)-demecolcinone (**3**) and metacolchicine (**4**), including the determination of their absolute configurations. Notably, demecolcinone (**3**), consisting of a novel and challenging azabicyclo[3.2.1]octane structure, was synthesized *via* an unusual oxidative dearomatization–azaspiroannulation reaction. It is noteworthy that an Ellman auxiliary was successfully used in the current study as a chiral directing group for the stereo- and enantioselective installation of the C7-acetamido group in a single step. Furthermore, the *in vitro* biological evaluation of compound **23** revealed that this material displayed the most potent inhibitory effects of all the compounds tested toward A549, MDA-MB-231, and LoVo cells (IC_50_ = ∼3.0 nM), as well as more potent inhibition of tubulin assembly than colchicine (**1**). These results therefore suggest that colchicinoid **23** could be used as a promising lead for the development of novel anticancer agents with improved properties.
