# Supplementary material for: Enantioselective total synthesis of (–)-colchicine, (+)-demecolcinone and metacolchicine: determination of the absolute configurations of the latter two alkaloids
Source: Chem Sci. 2017 May 5;8(7):4961–6. doi: 10.1039/c7sc01341h (PMC5607857; doi:10.1039/c7sc01341h)
Supplement: Supplementary file 1 [file SC-008-C7SC01341H-s001.pdf]

Electronic Supplementary Material (ESI) for Chemical Science.
This journal is © The Royal Society of Chemistry 2017

Supporting Information

Enantioselective Total Synthesis of (–)-Colchicine, (+)-Demecolcinone and Metacolchicine: Determination of the Absolute Configurations of the Latter Two Alkaloids

Bo Chen,[†,#] Xin Liu,[†,‖,#] Ya-Jian Hu,[†] Dong-Mei Zhang,[‡] Lijuan Deng,[‡] Jieyu Lu,[†] Long Min,[†] Wen-Cai Ye[‡] and Chuang-Chuang Li*,[†]

[†]Department of Chemistry, South University of Science and Technology of China, Shenzhen 518055, P. R. China

[‡]College of Pharmacy, Jinan University, Guangzhou 510632, P. R. China

[‖] Institute of Chinese Medical Sciences, University of Macau, Macao, China

[#] *These authors contributed equally to the work.*

Table of Contents

I. **General Information……………………………………………………………………………S3**

II. **Experimental Procedures…………………………………………………………………S4–S25**

III. **HPLC Analysis of *ee* Value……………………………………………………………..S26–S29**

IV. **X-ray Crystallographic Data………………………………………………………………..S30-S32**

V. **^{1}H and ^{13}C NMR Spectra…………………………………………………………………S33-S56**

VI. **Biological Study…..…………………………………………………………………………S57-S61**

VII. **Table S4: A Summary of Colchicine Syntheses……………………………………S62-S63**

VIII. **Table S5: A Summary of the Introduction of C-7-Amino Chiral Center…….S64**

IX. **References………………………………………………………………………………………S65**

I. General Information

Unless otherwise mentioned, all reactions were carried out under a nitrogen atmosphere under anhydrous conditions and all reagents were purchased from commercial suppliers without further purification. Solvent purification was conducted according to *Purification of Laboratory Chemicals* (Peerrin, D. D.; Armarego, W. L. and Perrins, D. R., Pergamon Press: Oxford, 1980). Yields refer to chromatographically and spectroscopically (^{1}H NMR) homogeneous materials, unless otherwise stated. Reactions were monitored by Thin Layer Chromatography on plates (GF254) supplied by Yantai Chemicals (China) using UV light as visualizing agent, an ethanolic solution of phosphomolybdic acid, or basic aqueous potassium permanganate ($KMnO_4$), and heat as developing agents. If not specially mentioned, flash column chromatography uses silica gel (200-300 mesh) supplied by Tsingtao Haiyang Chemicals (China), Preparative thin layer chromatography (PTLC) separations were carried out 0.50 mm Yantai (China) silica gel plates. NMR spectra were recorded on Bruker AV500, Bruker ARX400, and calibrated using residual undeuterated solvent as an internal reference ($CHCl_3$, δ 7.26 ppm ^{1}H NMR, δ 77.00 ^{13}C NMR). The following abbreviations were used to explain the multiplicities: s = singlet, d = doublet, t = triplet, q = quartet, b = broad, m = multiplet.

High-resolution mass spectra (HRMS) were recorded on a Bruker Apex IV FTMS mass spectrometer using ESI (electrospray ionization). Infrared spectra were recorded on a Shimadzu IR Prestige 21, using thin films of the sample on KBr plates. Optical rotations were measured with a Rudolph autopol I automatic polarimeter using 10 cm glass cells with a sodium 589 nm filter.

Caution: Due to the cytotoxic of colchicine and its analogs, the associated experiments were operated carefully in the fume hoods with breathing mask and double gloves.

II. Experimental Procedures

Synthesis of 15

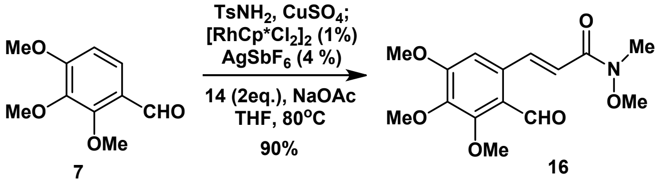

$TsNH_2$ (0.52 g, 3.06 mmol) and the aldehyde **7** (0.5 g, 2.55 mmol) were dissolved in THF (10 mL), then reactivated $CuSO_4$[a] (0.81 g, 5.1 mmol) was added. The mixture was heated at 120 °C for 36 h, cooled, and NaOAc (0.42 g, 5.1 mmol), $[RhCp*Cl_2]_2$ (16 mg, 0.026 mmol), $AgSbF_6$ (36 mg, 0.10 mmol), compound **14** (0.59 g, 5.1 mmol) were added to the system. The reaction mixture was stirred at 80 °C for 10h, cooled, and filtered through a pad of celite. The solids were washed with CH_2Cl_2, and the combined filtrates were evaporated in vacuo. Purification of the crude product by flash column chromatography on silica gel (hexane/ethyl acetate = 2/1) afforded **16** (0.71 g, 90% yield) as yellow oil.

[a] The $CuSO_4$ was reactivated at 80°C under vacuum for 3h.

Note 1: We got 5.5 gram of compound **16** in one pot operation in 70% yield on a 5.0 g scale.

Note 2: A traditional strategy involving Heck coupling of *ortho*-halogenated benzaldehyde **7a** (X = Br or I) with **14** could also be used for the synthesis of **16**. [1] However, in this strategy the preparation of **7a** from **7** would require three or four steps through an *ortho*-lithiation[2] or nitration[3,4] protocol, which would result in a low yield because of the difficulties associated with the direct halogenation of **7**. With this in mind, it is clear that the current scalable C–H bond activation strategy represents a considerably more efficient step- and atom-economical approach to the direct construction of **16**.

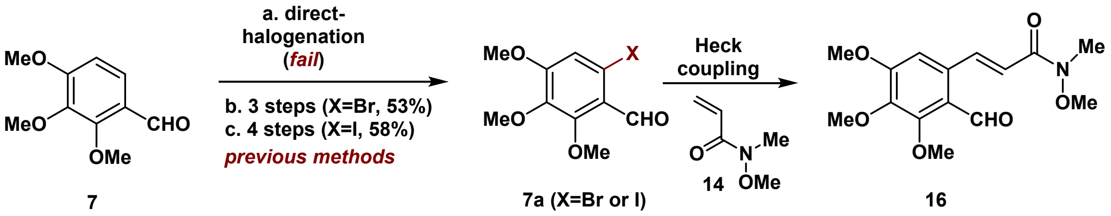

Note 3: It has been reported that aldehydes can be used as efficient directing groups for ruthenium-catalysed *ortho*-C–H alkenylation of aromatic aldehydes with activated alkenes.[5] However, our initial attempts to react **7** with various activated alkenes under the previously reported conditions failed to afford any of the desired products.

R_f = 0.3 (hexane/ethyl acetate = 2/1);

IR (film) 2938, 1653, 1595, 1490, 1196, 1124, 1033, 988 cm^{-1};

^{1}H NMR (500 MHz, $CDCl_3$) δ 10.35 (s, 1H), 8.31 (d, *J* = 15.7 Hz, 1H), 6.79 (s, 1H), 6.74 (d, *J* = 15.6 Hz, 1H), 3.94 (s, 3H), 3.93 (s, 3H), 3.85 (s, 3H), 3.70 (s, 3H), 3.24 (s, 3H);

^{13}C NMR (101 MHz, $CDCl_3$) *δ* 190.0, 166.2, 157.7, 157.5, 142.5, 142.3, 134.2, 121.5, 119.3, 106.9, 62.3, 61.8, 61.0, 56.2, 32.4;

HRMS (ESI) calcd for $C_{15}H_{20}O_6N[M+H]^+$: 310.1285; found: 310.1276.

Synthesis of 12

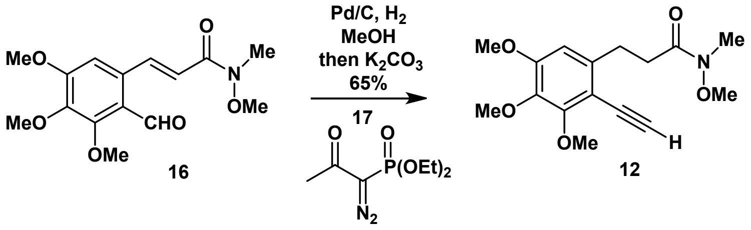

To a solution of **16** (1.0 g, 3.23 mmol) in MeOH (15 mL) was added Pd/C (0.1 g), and the resulting solution was saturated with hydrogen gas for 5 min, then stirred 30 minutes under the hydrogen atmosphere. Dimethyl (1-diazo-2-oxypropyl) phosphonate (0.85 g, 3.87 mmol) was added to the reaction system and cooled to 0 °C. K_2CO_3 (0.89 g, 6.46 mmol) was added and the resulting mixture was stirred for 10 min at 0 °C before allowed to warm to room temperature and stirred for 3 h. The reaction mixture was filtered through celite, and the filtrate was diluted with ether (60 mL) and washed with saturated aqueous $NaHCO_3$ (2 x 25 mL). The combined aqueous phases were re-extracted with ether (2 x 20 mL). The combined organic phase was washed with brine (20 mL), dried over Na_2SO_4, filtered and concentrated under reduced pressure. Purification of the crude product by flash column chromatography on silica gel (hexane/ethyl acetate = 5/1) afforded **12** (0.64 g, 65% yield) as a colorless oil.

R_f = 0.4 (hexane/ethyl acetate = 5/1);

IR (film) 2935,1674, 1647, 1558, 1494, 1381, 1330, 1255, 1130, 995 cm^{-1};

^{1}H NMR (400 MHz, Acetone) δ 6.79 (s, 1H), 3.90 (s, 3H), 3.89 (s, 1H), 3.88 (s, 3H), 3.78 (s, 3H), 3.68 (s, 3H), 3.14 (s, 3H), 3.06 – 2.97 (m, 2H), 2.79 – 2.70 (m, 2H);

^{13}C NMR (101 MHz, Acetone) δ 172.9, 155.6, 154.3, 140.7, 140.5, 108.8, 108.7, 84.6, 78.1, 60.7, 60.5, 60.2, 55.5, 32.3, 31.6, 29.4;

HRMS (ESI) calcd for $C_{16}H_{22}O_5N[M+H]^+$: 308.1493; found: 308.1484.

Synthesis of 18

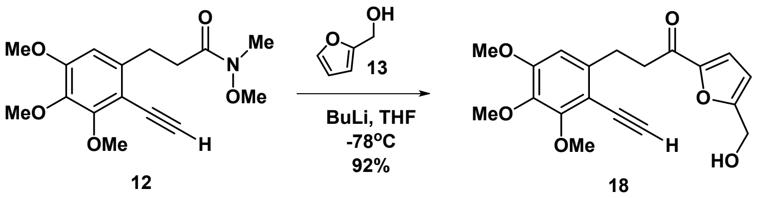

To a solution of furfuryl alcohol **13** (2.54 g, 26.0 mmol) in THF (150 mL) was added BuLi (21.6 mL, 2.4 M, 52.0 mmol) at -78°C. The reaction mixture was stirred at -78°C for 10 minutes, then warmed to 0 °C, and stirred for another 30 minutes. Compound **12** (4.0 g, 13.0 mmol) dissolved in THF (20 mL) was added dropwise. The reaction mixture was stirred for 3h, then poured into saturated aqueous NH_4Cl (100 mL) and dilute with ether (150 mL). After separation, the aqueous phase was re-extracted with ether (2 x 50 mL). The combined organic phase was washed with brine (100 mL), dried over Na_2SO_4, filtered and concentrated under reduced pressure. Purification of the crude product by flash column chromatography on silica gel (hexane/ethyl acetate = 1/1) afforded **18** (4.12 g, 92% yield) as a colorless oil.

R_f = 0.2 (hexane/ethyl acetate = 1/1);

IR (film) 2937, 1670, 1517, 1490, 1404,1197, 1122, 1029, 810 cm^{-1};

^{1}H NMR (400 MHz, $CDCl_3$) δ 7.14 (d, *J* = 3.5 Hz, 1H), 6.58 (s, 1H), 6.41 (d, *J* = 3.5 Hz, 1H), 4.67 (d, *J* = 5.9 Hz, 2H), 3.96 (s, 3H), 3.84 (s, 3H), 3.83 (s, 3H), 3.43 (s, 1H), 3.13 (t, *J* = 3.5 Hz, 4H);

^{13}C NMR (126 MHz, $CDCl_3$) δ 188.5, 159.1, 155.5, 154.0, 151.8, 140.3, 140.3, 118.8, 109.6, 108.6, 108.5, 84.1, 78.1, 61.3, 61.1, 57.5, 56.0, 38.9, 29.5;

HRMS (ESI) calcd for $C_{19}H_{21}O_6[M+H]^+$: 345.1333; found: 345.1324.

Asymmetric synthesis of 10

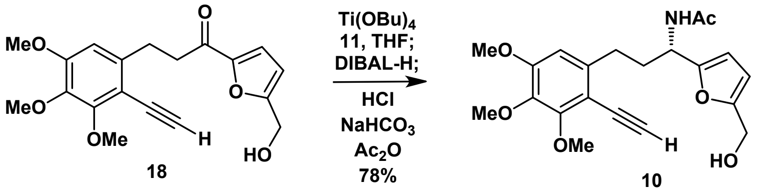

A solution of compound **18** (3.0 g, 8.72 mmol) in anhydrous THF (50 mL) was cooled to 0 °C and *R-tert*-butanesulfinamide **11** (1.27 g, 10.5 mmol) was added, followed by $Ti(OBu)_4$ (5.8 g, 17.0 mmol). The reaction

mixture was stirred at 120 °C for 12 h, and then cooled to -78°C. DIBAL-H (1 M, 17mL) was added dropwise, and the reaction mixture was allowed to warm to room temperature. Once the reduction was determined to be completed by TLC, the reaction mixture was cooled to 0 °C and MeOH (1 mL) was added dropwise until gas evolution was no longer observed. Aqueous solution of HCl (1M) was added until the pH = 1-2, and then the reaction mixture was stirred at room temperature for 1h. Saturated aqueous $NaHCO_3$ was added at 0 °C until pH of the reaction mixture was up to 10. MeOH (5 mL) was added to the reaction mixture, followed by slowly addition of Ac_2O. The reaction mixture was evaporated under reduced pressure, and the aqueous phase was extracted with ethyl acetate (50 mL x 2). The combined organic phase was washed with brine (15 mL), dried over Na_2SO_4, filtered and concentrated under reduced pressure. Purification of the crude product by flash column chromatography on silica gel (hexane/ethyl acetate = 1/2) afforded alcohol **10** (2.63 g, 78% yield) as colorless oil.

R_f = 0.3 (hexane/ethyl acetate = 1/2);

[α]20 D = 62.9 (c = 1.0, $CHCl_3$);

IR (film) 3280, 2938, 1655, 1491, 1404, 1337, 1123, 797 cm^{-1};

^{1}H NMR (400 MHz, $CDCl_3$) δ 6.52 (s, 1H), 6.18 (d, *J* = 3.1 Hz, 1H), 6.14 (d, *J* = 3.2 Hz, 1H), 5.98 (d, *J* = 8.8 Hz, 1H), 5.16 – 5.06 (m, 1H), 4.54 (s, 2H), 3.93 (s, 3H), 3.86 (s, 3H), 3.82 (s, 3H), 3.38 (s, 1H), 2.82 – 2.73 (m, 1H), 2.72 – 2.62 (m, 1H), 2.22 – 2.12 (m, 1H), 2.10 – 2.04 (m, 1H), 1.98 (s, 3H);

^{13}C NMR (101 MHz, $CDCl_3$) δ 169.6, 155.6, 154.2, 154.2, 153.6, 140.7, 140.3, 108.64, 108.59, 108.4, 107.3, 83.8, 78.4, 61.4, 61.1, 57.5, 56.2, 47.2, 34.6, 31.4, 23.5;

HRMS-ESI calcd for $C_{21}H_{26}O_6N[M+H]^+$: 388.1755, found 388.1746.

Synthesis of 8

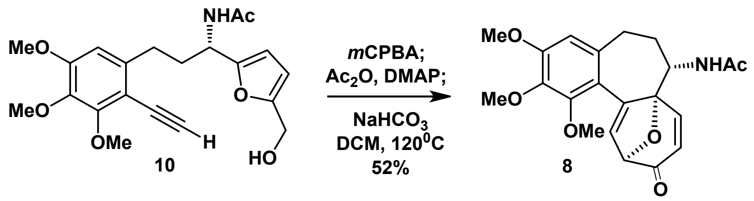

A solution of **10** (1.0 g 2.58 mmol) in DCM (25 mL) at 0 °C was treated with *meta*-chloroperoxybenzoic acid (*m*CPBA) (0.72 g, 3.1 mmol) and the resulting solution was stirred at 0 °C for 30 minutes. The mixture was allowed to warm to room temperature and stirred for 2 h. Once the reaction was complete, as determined by TLC analysis, the reaction was cooled to 0 °C and Ac_2O (0.53 g, 5.16 mmol) was added. DMAP (0.68 g, 5.16 mmol) was added with portions. The resulting reaction solution was stirred at 0 °C for 10 minutes. DCM (100 mL) was

added, followed by $NaHCO_3$ (0.43 g, 5.16 mmol) and the reaction mixture was heated at 120 °C for 20h in a sealed tube. After cooled to room temperature, the reaction solution was washed with water (150 mL), brine (100 mL) and dried over Na_2SO_4. Filtered and concentrated under reduced pressure, the residue obtained was purified by flash column chromatography on silica gel (hexane/ethyl acetate = 1/2) afforded **8** (0.51 g, 52% yield) as a white solid.

$\boldsymbol{R_f}$ = 0.4 (hexane/ethyl acetate = 1/2);

[α]20 D = -158.4 (c = 0.57, $CHCl_3$);

IR (film) 3267, 1634, 1595, 1539, 1408, 1333, 1098, 803 cm^{-1};

^{1}H NMR (400 MHz, $CDCl_3$) δ 7.12 (d, *J* = 9.9 Hz, 1H), 6.53 (s, 1H), 6.30 (d, *J* = 2.5 Hz, 1H), 5.98 (d, *J* = 9.2 Hz, 1H), 5.60 (dd, *J* = 9.9, 1.5 Hz, 1H), 5.06 – 5.03 (m, 1H), 4.34 – 4.23 (m, 1H), 3.85 (s, 3H), 3.84 (s, 3H), 3.67 (s, 3H), 2.95 – 2.85 (m, 1H), 2.85 – 2.76 (m, 1H), 2.10 – 2.01 (m, 1H), 1.98 (s, 3H), 1.91 – 1.84 (m, 1H);

^{13}C NMR (101 MHz, $CDCl_3$) δ 193.0, 169.6, 154.3, 153.8, 150.8, 148.2, 141.0, 133.8, 127.1, 123.8, 118.5, 109.4, 90.5, 88.0, 62.0, 61.0, 56.1, 49.7, 31.3, 26.5, 23.4;

HRMS (ESI) calcd for $C_{21}H_{24}O_6N[M+H]^+$: 386.1598, found 386.1593.

Synthesis of 19

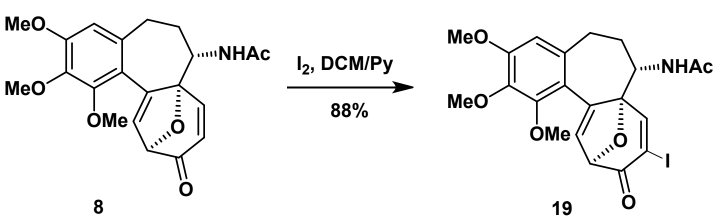

8 19

To a solution of **8** (0.60 g, 1.56 mmol) in DCM/pyridine (10 mL/10 mL), was added I_2 (0.47 g, 1.87 mmol) in one portion. The resulting reaction mixture was heated at 60°C in a sealed tube for 10h. The reaction solution was poured into saturated $Na_2S_2O_3$ (50 mL), and extracted with DCM (20 mL X 2). The combined organic phase was washed successively with aqueous HCl (1 M, 100 mL), saturated aqueous $NaHCO_3$ (30 mL), brine (30 mL), then dried over Na_2SO_4, filtered and concentrated under reduced pressure. Purification of the crude product by flash column chromatography on silica gel (hexane/ethyl acetate = 1/1) afforded **19** (0.71 g, 88% yield) as a white solid.

$\boldsymbol{R_f}$ = 0.25 (hexane/ethyl acetate = 1/1);

[α]20 D = 29.0 (c = 1.0, $CHCl_3$);

IR (film) 2954, 2922, 2848, 1714, 1651, 1568, 1539, 1456, 1136,1095 cm^{-1};

^{1}H NMR (500 MHz, $CDCl_3$) δ 7.87 (d, *J* = 3.4 Hz, 1H), 6.53 (s, 1H), 6.33 (d, *J* = 2.2 Hz, 1H), 6.22 (d, *J* = 9.1 Hz, 1H), 5.31 (d, *J* = 2.2 Hz, 1H), 4.37 – 4.29 (m, 1H), 3.85 (s, 3H), 3.84 (s, 3H), 3.66 (s, 3H), 2.93 – 2.77 (m, 2H), 2.12 – 2.04 (m, 1H), 2.01 (s, *3*H), 1.84 (ddd, *J* = 13.8, 7.9, 3.8 Hz, 1H).

^{13}C NMR (126 MHz, $CDCl_3$) δ 186.23, 169.83, 162.23, 153.91, 150.57, 148.79, 140.86, 133.67, 126.83, 117.81, 109.28, 97.06, 93.14, 86.51, 61.85, 60.88, 56.04, 49.32, 31.20, 26.51, 23.34.

HRMS-ESI calcd for $C_{21}H_{23}O_6NI[M+H]^+$: 512.0574, found 512.0565.

Synthesis of 20

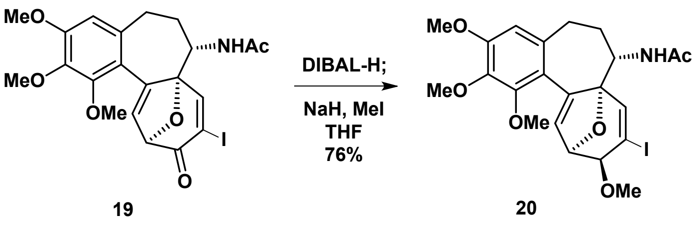

To the solution of **19** (1.0 g, 1.96 mmol) in THF (30 mL) at -78°C was added DIBAL-H (1M, 5.0 mL), and the reaction solution was stirred at this temperature for 1h. H_2O (0.1 mL, 5.6 mmol) was added and stirred at 0°C for 30 minutes. NaH (60%, 200 mg, 5.0 mmol) was added, followed by MeI (0.70g, 5.0 mmol), and the resulting mixture was stirred at room temperature for 24h. The reaction solution was quenched with saturated aqueous potassium sodium tartrate (50 mL) and diluted with ether (100 mL). After separation, the aqueous phase was re-extracted with ether (50 mL x 2). The combined organic phase was washed with brine (30 mL), dried over Na_2SO_4, filtered and concentrated under reduced pressure. Purification of the crude product by flash column chromatography on silica gel (hexane/ethyl acetate = 1/2) afforded **20** (0.78 g, 76% yield) as a white solid.

R_f = 0.26 (hexane/ethyl acetate = 1/1);

[α]20 D = 190.1 (c = 1.0, $CHCl_3$);

IR (film) 2935, 1462, 1408, 1328, 1317, 1236, 1195, 1024 cm^{-1};

^{1}H NMR (500 MHz, $CDCl_3$) δ 6.80 (d, *J* = 1.2 Hz, 1H), 6.52 (s, 1H), 6.10 (d, *J* = 2.0 Hz, 1H), 5.88 (d, *J* = 8.8 Hz, 1H), 5.39 (dd, *J* = 5.6, 2.0 Hz, 1H), 4.08 (m, 2H), 3.88 (s, 3H), 3.87 (s, 3H), 3.77 (s, 3H), 3.60 (s, 3H), 2.88 (ddd, *J* = 23.0, 15.7, 8.3 Hz, 2H), 2.01 (s, 3H), 2.00 – 1.96 (m, 1H), 1.85 – 1.77 (m, 1H).

^{13}C NMR (126 MHz, $CDCl_3$) δ 169.57, 153.38, 151.10, 148.30, 146.95, 140.92, 134.25, 126.56, 118.35, 109.25, 100.35, 90.36, 80.98, 77.20, 62.21, 60.95, 58.92, 56.00, 49.94, 31.54, 26.42, 23.48.

HRMS-ESI calcd for $C_{22}H_{27}O_6NI[M+H]^+$: 528.0883, found 528.0878.

Synthesis of 21

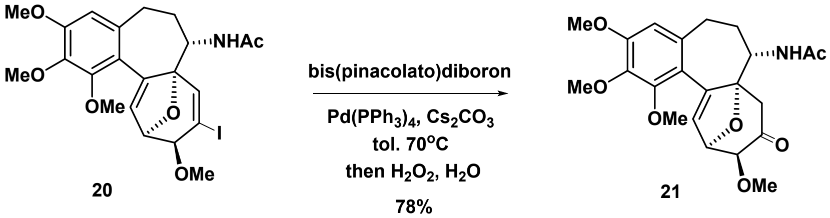

To an over dried round bottom flask, was added **20** (1.2 g, 2.3 mmol), bis(pinacolato)diboron (0.87 g, 3.4 mmol), Cs_2CO_3 (1.5 g, 4.6 mmol), $Pd(PPh_3)_4$ (0.26 g, 0.23 mmol), then degassed with argon for 3 times. Toluene (20 mL) was added, and the resulting mixture was stirred at 70 ^oC for 6 h. H_2O (5 mL) was added at 0 ^oC, followed by H_2O_2 (2 mL, 30%) which was added dropwise. The reaction mixture was stirred at room temperature for 1h, and then was diluted with ethyl acetate (100 mL). The organic phase was separated, then washed with saturated aqueous $Na_2S_2O_3$ (30 mL), brine (30 mL), dried over Na_2SO_4, filtered and concentrated under reduced pressure. Purification of the crude product by flash column chromatography on silica gel (hexane/ethyl acetate = 2/1) afforded **21** (0.75 g, 78% yield) as a white solid.

$\boldsymbol{R_f}$ = 0.2 (hexane/ethyl acetate = 1/2);

[α]20 D = 86.7 (c = 0.5, $CHCl_3$);

IR (film) 2932, 1651, 1504, 1198, 1308, 799 cm^{-1};

^1H NMR (500 MHz, $CDCl_3$) δ 6.45 (s, 1H), 6.19 (d, *J* = 1.8 Hz, 1H), 5.90 (d, *J* = 9.2 Hz, 1H), 5.13 (dd, *J* = 4.9, 1.9 Hz, 1H), 4.05 – 3.98 (m, 1H), 3.96 (d, *J* = 4.9 Hz, 1H), 3.84 (s, 3H), 3.81 (s, 3H), 3.75 (s, 3H), 3.66 (s, 3H), 3.02 – 2.95 (m, 1H), 2.89 – 2.82 (m, 1H), 2.64 (d, *J* = 16.0 Hz, 1H), 2.27 (d, *J* = 16.0 Hz, 1H), 2.19 – 2.12 (m, 1H), 2.02 (s, 3H), 1.83 – 1.76 (m, 1H);

^{13}C NMR (126 MHz, $CDCl_3$) δ 204.2, 169.5, 153.6, 151.2, 142.1, 140.4, 134.2, 128.9, 117.4, 108.6, 89.8, 84.8, 78.8, 61.3, 60.7, 59.9, 56.0, 50.0, 47.3, 31.7, 27.1, 23.5;

HRMS-ESI calcd for $C_{22}H_{28}O_7N[M+H]^+$: 418.1860, found 418.1852.

Synthesis of colchicine (1)

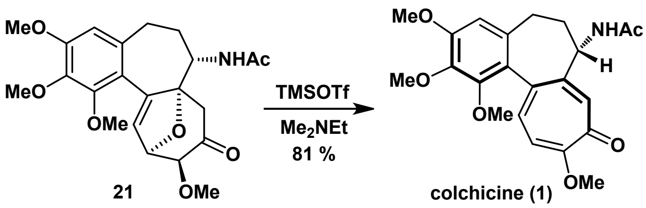

Trimethylsilyl trifluoromethanesulfonate (0.8 mL) was added dropwise at 0 °C to a solution of **21** (280 mg, 0.68 mmol) and N,N-Dimethylethylamine (1.2 mL) in DCM (20 mL). After the reaction mixture was stirred at room temperature for 12 h, saturated aqueous $NaHCO_3$ (20 mL) was added. After separation, the aqueous phase was further extracted with DCM (20 mL x 2). The combined organic phases were washed with saturated NH_4Cl (10 mL), brine (10 mL) and dried over Na_2SO_4. The solution was concentrated under reduced pressure and purified by chromatography (DCM/MeOH = 9/1) to afford **1** (220 mg, 81%) as a white solid.

R_f = 0.3 (DCM/MeOH = 9/1);

[α]20 D = -150 (c = 0.33, $CHCl_3$);

IR (film) 3315, 3240, 2935, 1732, 1614, 1487, 1249, 1138, 1020 cm^{-1};

^{1}H NMR (500 MHz, $CDCl_3$) δ 8.42 (d, *J* = 4.4 Hz, 1H), 7.68 (s, 1H), 7.37 (d, *J* = 10.7 Hz, 1H), 6.92 (d, *J* = 10.9 Hz, 1H), 6.53 (s, 1H), 4.74 – 4.57 (m, 1H), 4.02 (s, 3H), 3.93 (s, 3H), 3.90 (s, 3H), 3.65 (s, 3H), 2.52 (m, 1H), 2.41 – 2.32 (m, 2H), 2.02 – 1.97 (m, 1H), 1.95 (s, 3H).

^{13}C NMR (126 MHz, $CDCl_3$) δ 179.3, 170.2, 164.0, 153.5, 153.0, 151.1, 141.5, 137.2, 135.8, 134.3, 130.3, 125.5, 113.2, 107.2, 61.6, 61.3, 56.4, 56.1, 52.8, 36.3, 29.8, 22.7;

HRMS-ESI calcd for $C_{22}H_{26}O_6N[M+H]^+$: 400.1755, found 400.1747.

Note: Using the described route, a total of 1.1 g of colchicine was prepared readily after five simple parallel operations.

Racemic synthesis of (±)-10 for HPLC analysis

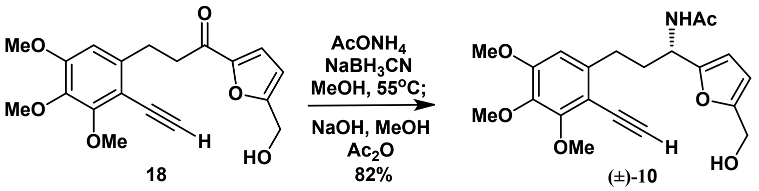

To a solution of $AcONH_4$ (4.9 g, 64.0 mmol) in methanol (30 mL) was added **18** (2.2 g, 6.4mmol), the resulting mixture was cooled to 0 °C. $NaBH_3CN$ (2.0 g, 32.0 mmol) was added with portions. The mixture was stirred for 24 h at 55 °C and cooled to 0 °C. Aqueous NaOH (1M, 10 mL) was added, followed by MeOH (5 mL). To the resulting

mixture was added Ac_2O (3 mL). After the reaction mixture was stirred at room temperature for 10 minutes, the reaction mixture was evaporated under reduced pressure. The residue was extracted with DCM (50 mL x 2), and the combined organic phase was washed with saturated aqueous NH_4Cl (20 mL), brine (20 mL), dried over Na_2SO_4, filtered and concentrated under reduced pressure. Purification of the crude product by flash column chromatography on silica gel (hexane/ethyl acetate = 1/2) afforded **(±)-10** (2.03 g, 82% yield) as colorless oil.

The synthesis of (±)-colchicine (1) from (±)-10 was using the same route as described above.

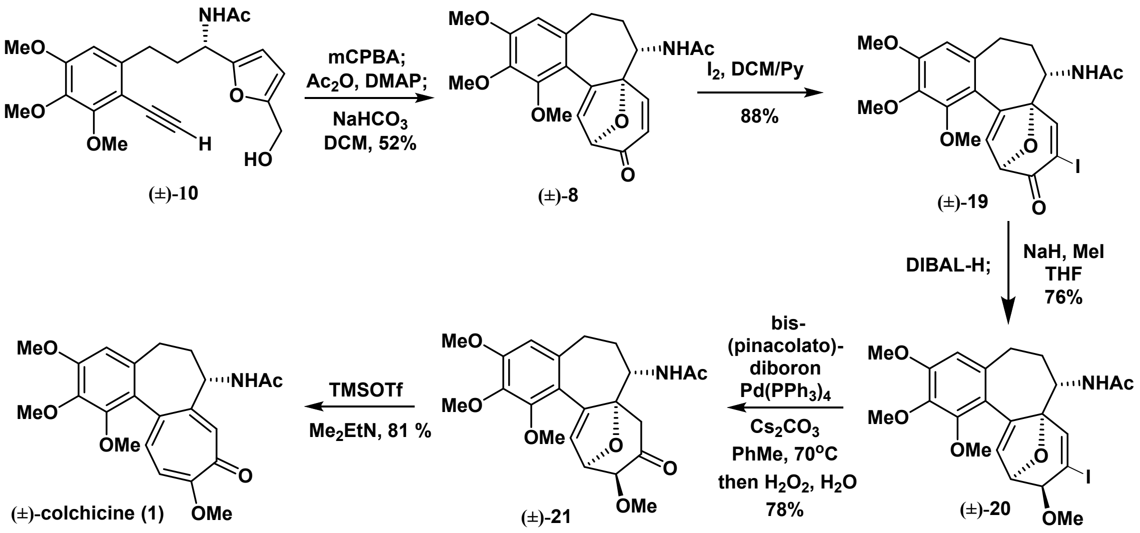

Synthesis of (+)-(a*S*,7*R*)-colchicine (2, *ent*-1):

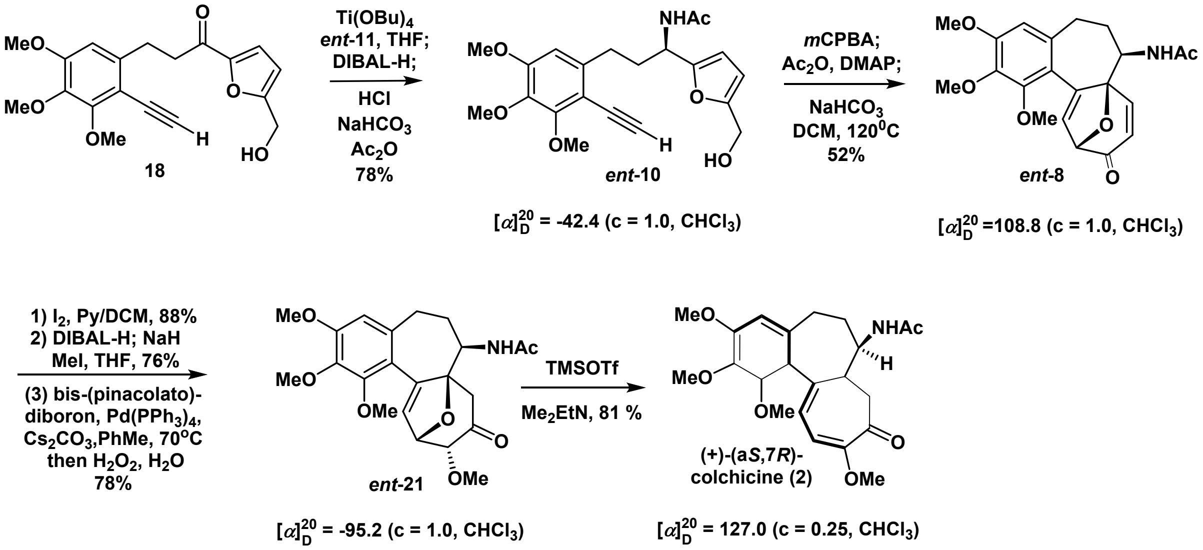

Synthesis of N-Boc-N-deacetylcolchicine (30)

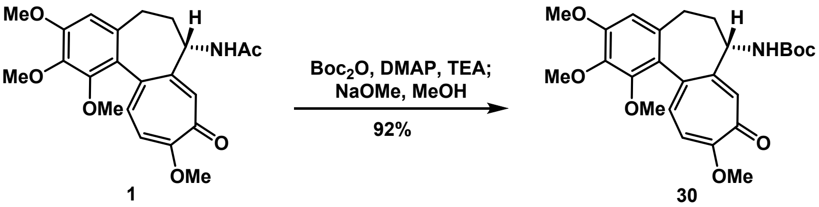

A mixture of colchicine **1** (172 mg, 0.43 mmol), Boc_2O (0.25 mL, 1.59 mmol), DMAP (60.2 mg, 0.43 mmol) and trimethylamine (0.12 mL, 0.86 mmol) in CH_3CN (15 mL) was refluxed for 10 h. The mixture was extracted with $CHCl_3$ (30 mL), washed with 10% citric acid (3 mL) followed by brine (5 mL). Concentration under vacuum gave the crude product, which was treated with NaOMe (45.2 mg, 0.84 mmol) in MeOH (7.5 mL) at 0°C for 0.5 h. The reaction was worked up by addition of saturated aqueous NH_4Cl (6 mL), followed by extraction with DCM (3 x 10 mL). The combined organic extracts were washed with brine (6 mL) and then were dried over by $MgSO_4$. The solvents were removed under vacuum, and the residue was purified by a flash column chromatography on silica gel (DCM/MeOH = 25/1) to give product N-Boc-N-deacetylcolchicine **30** (181 mg, 92%) as a yellowish solid.

R_f = 0.65 (DCM/MeOH = 8/1);

^{1}H NMR (400 MHz, $CDCl_3$) δ 7.48 (s, 1H), 7.22 (d, *J* = 10.7 Hz, 1H), 6.78 (d, *J* = 10.8 Hz, 1H), 6.50 (s, 1H), 5.06 (d, *J* = 7.6 Hz, 1H), 4.36 (dt, *J* = 11.8, 7.0 Hz, 1H), 3.96 (s, 3H), 3.89 (s, 3H), 3.86 (s, 3H), 3.61 (s, 3H), 2.47 (dd, *J* = 13.2, 6.1 Hz, 1H), 2.35 (td, *J* = 13.0, 6.5 Hz, 1H), 2.29 – 2.17 (m, 1H), 1.67 (td, *J* = 11.6, 6.2 Hz, 1H), 1.32 (s, 9H);

^{13}C NMR (101 MHz, $CDCl_3$) δ 179.53, 163.90, 154.36, 153.42, 151.28, 151.12, 141.56, 136.10, 135.01, 134.28, 131.18, 125.61, 112.15, 107.23, 79.80, 61.48, 61.31, 56.29, 56.08, 53.09, 37.62, 29.97, 28.29;

HRMS (ESI) Calcd for $C_{25}H_{32}NO_7$ $[M+H]^+$: 458.2173; Found: 458.2175.

Synthesis of N-deacetylcolchicine (31)

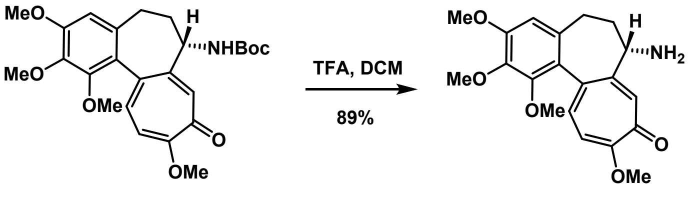

30 31

N-Boc-N-deacetylcolchicine **30** (64 mg, 0.13 mmol) was dissolved in DCM (5 mL), and trifluoroacetic acid (24 mg, 0.21 mmol) was added. The reaction mixture was stirred at room temperature for 3 h. Aqueous 1N NaOH was added into the mixture until the pH was 10. It was extracted with DCM (15 mL) and concentrated under reduced pressure. The crude product was purified by column chromatography (DCM/MeOH = 10/1) to give pure N-deacetylcolchicine **31** as a pale yellow foam (45 mg, 89%). Anal. data are consistent with those described in

reference[6].

R$_f$ = 0.2 (DCM/MeOH = 10/1);

[α]26 D = - 82 (c = 1.0, $CHCl_3$) {lit. [α] - 152 (c = 1.17, $CHCl_3$)}[6];

^{1}H NMR (400 MHz, $CDCl_3$) δ 7.67 (s, 1H), 7.12 (d, *J* = 10.7 Hz, 1H), 6.74 (d, *J* = 10.8 Hz, 1H), 6.47 (s, 1H), 3.92 (s, 3H), 3.84 (s, 3H), 3.83 (s, 3H), 3.67 – 3.61 (m, 1H), 3.58 (s, 3H), 2.44 – 2.18 (m, 3H), 1.55 (dd, *J* = 10.6, 5.3 Hz, 1H);

^{13}C NMR (101 MHz, $CDCl_3$) δ 179.56, 163.70, 154.21, 153.26, 150.65, 141.06, 136.35, 135.15, 134.52, 131.69, 125.39, 111.79, 106.95, 61.10, 60.99, 56.22, 56.01, 53.66, 40.34, 30.54;

HRMS (ESI) Calcd for $C_{20}H_{24}NO_5$ $[M+H]^+$: 358.1649; Found: 358.1648.

Synthesis of N-formyl-N-deacetylcolchicine (23)

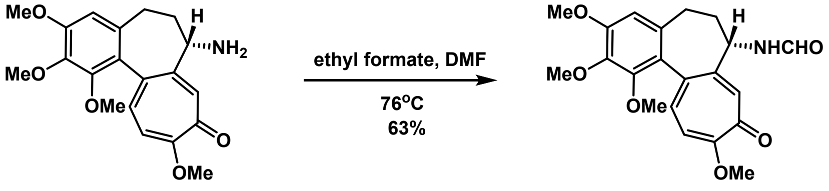

A mixture of N-deacetylcolchicine **31** (25 mg, 0.07 mmol), and ethyl formate (0.06 mL, 0.7 mmol) in DMF (10 mL) was stirred at 76 °C for 8 h. The reaction was quenched by addition of water (5 mL), followed by extraction with Et_2O (3 x 15 mL). The combined organic extracts were dried over by $MgSO_4$. The solvents were removed under reduced pressure, and the residue was purified by a flash column chromatography on silica gel (DCM/MeOH = 20/1) to give product N-formyl-N-deacetylcolchicine **23** (17 mg, 63%) as a yellowish solid.

R$_f$ = 0.4 (DCM/MeOH = 10/1);

[α]26 D = - 124 (c = 1.0, $CHCl_3$) {lit. [α] - 175 (c = 1.0, $CHCl_3$)}[7];

^{1}H NMR (400 MHz, $CDCl_3$) δ 8.26 (d, *J* = 6.8 Hz, 1H), 8.14 (s, 1H), 7.59 (s, 1H), 7.33 (d, *J* = 10.8 Hz, 1H), 6.88 (d, *J* = 10.8 Hz, 1H), 6.52 (s, 1H), 4.77 – 4.60 (m, 1H), 3.99 (s, 3H), 3.92 (s, 3H), 3.88 (s, 3H), 3.62 (s, 3H), 2.56 – 2.45 (m, 1H), 2.34 (ddd, *J* = 18.2, 12.9, 6.4 Hz, 2H), 2.00 – 1.88 (m, 1H);

^{13}C NMR (101 MHz, $CDCl_3$) δ 179.61, 164.10, 161.13, 153.65, 151.67, 151.22, 141.66, 136.82, 135.80, 134.24, 130.78, 125.48, 113.03, 107.39, 61.65, 61.45, 56.54, 56.18, 51.25, 36.60, 29.90;

HRMS (ESI) Calcd for $C_{21}H_{24}NO_6$ $[M+H]^+$: 386.1598; Found: 386.1598.

Synthesis of demecolcine (22)

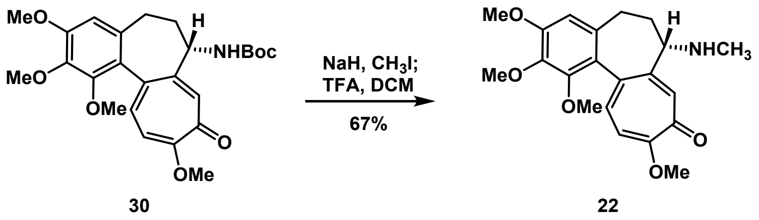

To a solution of N-Boc-N-deacetylcolchicine **30** (110 mg, 0.48 mmol) and NaH (19.7 mg, 0.49 mmol) in DMF (10 mL) was added CH_3I (0.034 mL, 0.49 mmol) dropwise at 0 °C, and the mixture was stirred at the same temperature for 5 minutes, followed by stirring at room temperature for 10 minutes. The reaction was quenched by water (5 mL), followed by extraction with Et_2O (3 x 15 mL). The combined organic extracts were dried over by $MgSO_4$. The solvents were removed under reduced pressure to give the crude product, which was used directly in the next step. To the solution of the crude product in DCM (10 mL) was added TFA (34 mg, 0.3 mmol) at 25 °C. The resulting mixture was stirred at the same temperature for 20 minutes, then quenched with saturated aqueous $NaHCO_3$ (5 mL) carefully. The mixture was extracted with DCM (3 x 15 mL). The combined organic extracts were washed with brine (8 mL) and then were dried over by $MgSO_4$. The solvents were removed under vacuum, and the residue was purified by a flash column chromatography on silica gel (DCM/MeOH = 15/1) to give product demecolcine **22** (60 mg) in 67% yield for two steps as a yellowish solid. Anal. data are consistent with those described in reference[8].

R_f = 0.32 (DCM/MeOH = 8/1);

[α]26 D = - 93 (c = 1.0, $CHCl_3$) {lit. [α] - 129 (c = 1.0, $CHCl_3$)}[9];

^{1}H NMR (400 MHz, $CDCl_3$) δ 7.64 (s, 1H), 7.17 (d, *J* = 10.7 Hz, 1H), 6.75 (d, *J* = 10.8 Hz, 1H), 6.48 (s, 1H), 3.94 (s, 3H), 3.86 (s, 3H), 3.85 (s, 3H), 3.54 (s, 3H), 3.20 (dd, *J* = 10.9, 6.2 Hz, 1H), 2.40 (dd, *J* = 13.4, 6.2 Hz, 1H), 2.31 (dt, *J* = 13.3, 6.6 Hz, 1H), 2.18 – 2.09 (m, 4H), 1.58 (dd, *J* = 11.4, 4.8 Hz, 1H);

^{13}C NMR (101 MHz, $CDCl_3$) δ 179.72, 163.82, 153.22, 150.65, 150.56, 141.17, 137.11, 135.15, 134.64, 132.11, 125.62, 111.82, 107.14, 62.59, 61.24, 60.73, 56.21, 56.02, 38.55, 34.42, 30.24;

HRMS (ESI) Calcd for $C_{21}H_{26}NO_5$ $[M+H]^+$: 372.1805; Found: 372.1808.

Synthesis of 2-demethyldemecolcine (24)

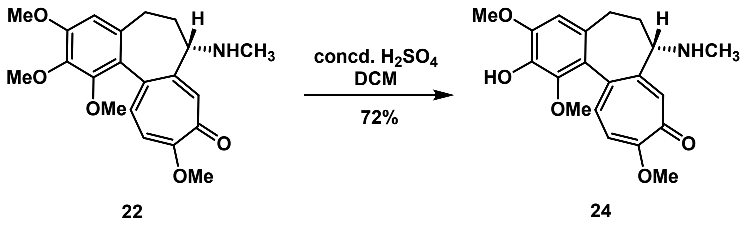

To a solution of demecolcine **22** (60 mg, 0.16 mmol) in DCM (0.5 mL) was added concd. H_2SO_4 (2.5 mL) at 25 °C, and then the mixture was stirred at 45 °C for 4-5 h. The reaction was worked up by pouring the mixture to the ice and then quenched by addition of NaOH (2 M solution) carefully until the pH was 6 - 7. The mixture was extracted with DCM (3 x 15 mL). The combined organic extracts were washed with brine (8 mL) and then were dried over by $MgSO_4$. The solvents were removed under reduced pressure, and the residue was purified by a flash column chromatography on silica gel (DCM/MeOH = 50/1-10/1) to give the product 2-demethyldemecolcine **24** (42 mg, 72%) as yellowish solid.

$\boldsymbol{R_f}$ = 0.3 (DCM/MeOH = 8/1);

[α]26 D = - 124 (c = 0.1, $CHCl_3$) {lit. [α] - 128 (c = unknown, $CHCl_3$)}[10];

IR (film) 2943, 2821, 1601, 1478, 1079, 852 cm^{-1};

^{1}H NMR (400 MHz, $CDCl_3$) δ 7.68 (s, 1H), 7.26 (d, *J* = 10.7 Hz, 1H), 6.80 (d, *J* = 10.8 Hz, 1H), 6.52 (s, 1H), 4.00 (s, 3H), 3.93 (s, 3H), 3.56 (s, 3H), 3.35 (dd, *J* = 11.0, 6.1 Hz, 1H), 2.45 (dd, *J* = 13.1, 6.2 Hz, 1H), 2.40 – 2.32 (m, 1H), 2.28 (s, 3H), 2.24 (d, *J* = 6.2 Hz, 1H), 1.73 (td, *J* = 11.2, 6.0 Hz, 1H);

^{13}C NMR (101 MHz, $CDCl_3$) δ 179.8, 163.9, 150.5, 147.3, 144.2, 137.4, 137.1, 134.4, 132.2, 130.7, 125.0, 111.9, 106.6, 62.7, 60.3, 56.3, 56.3, 38.8, 34.4, 30.0;

HRMS (ESI) Calcd for $C_{20}H_{24}NO_5$ $[M+H]^+$: 358.1649; Found: 358.1647.

Synthesis of O-methyl demecolcinone (32)

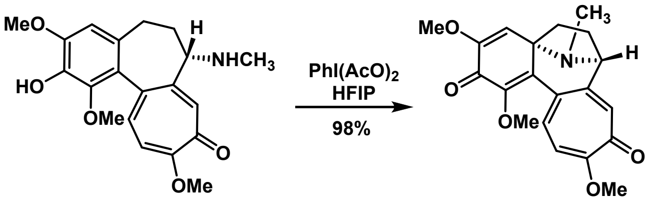

24 32

To a solution of 2-demethyldemecolcine **24** (42 mg, 0.12 mmol) in $(CF_3)_2CHOH$ (HFIP, 5 mL) was added $PhI(OAc)_2$ (57 mg, 0.18 mmol) at 25 °C, and then the mixture was stirred at the same temperature for 5 minutes. The reaction was quenched by addition of saturated aqueous $Na_2S_2O_3$ (2.5 mL). The mixture was extracted with DCM (3 x 10 mL). The combined organic extracts were washed with brine (5 mL) and then were dried over by $MgSO_4$. The solvents were removed under reduced pressure, and the residue was purified by a flash column chromatography on silica gel (DCM/MeOH = 50/1) to give the product **32** (48.5 mg) in 98% yield as yellow solid.

$\boldsymbol{R_f}$ = 0.55 (DCM/MeOH = 15/1);

IR (film) 2929, 2860, 1547, 1446, 1232, 970 cm^{-1};

^{1}H NMR (400 MHz, $CDCl_3$) δ 8.34 (d, *J* = 10.9 Hz, 1H), 7.00 (s, 1H), 6.75 (d, *J* = 11.0 Hz, 1H), 5.68 (s, 1H), 4.00 (d, *J* = 4.7 Hz, 4H), 3.73 (s, 3H), 3.66 (s, 3H), 2.41 (dd, *J* = 12.3, 6.1 Hz, 1H), 2.26 (dd, *J* = 13.1, 3.1 Hz, 1H), 2.21 (s, 3H), 2.11 – 1.98 (m, 1H), 1.63 (ddd, *J* = 12.5, 8.1, 3.2 Hz, 1H);

^{13}C NMR (101 MHz, $CDCl_3$) δ 179.5, 178.1, 164.7, 151.2, 149.0, 148.1, 139.3, 135.9, 133.3, 129.8, 118.6, 111.2, 70.5, 63.7, 60.4, 56.6, 55.4, 36.0, 32.5, 31.7;

HRMS (ESI) Calcd for $C_{20}H_{22}NO_5$ $[M+H]^+$: 356.1492; Found: 356.1493.

Synthesis of (+)-demecolcinone (3)

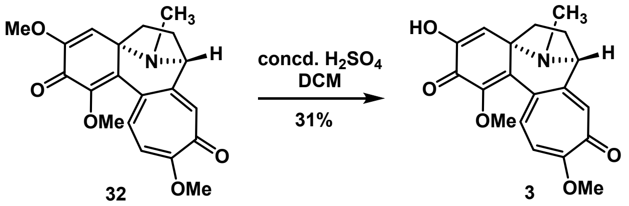

To a solution of **32** (10 mg, 0.028 mmol) in DCM (0.2 mL) was added concd. H_2SO_4 (0.4 mL) at 25 °C, and then the mixture was stirred at 45 °C for 4-5 h. The reaction was worked up by pouring the mixture to the ice and then quenched by addition of NaOH (2 M solution) carefully until the pH was 6 - 7. The mixture was extracted with DCM (3 x 4 mL). The combined organic extracts were washed with brine (2 mL) and then were dried over by $MgSO_4$. The solvents were removed under reduced pressure, and the residue was purified by a flash column chromatography on silica gel (DCM/MeOH = 50/1-15/1) to give the inseparable mixed products as white solids. Then the mixed products were separated by preparative HPLC. Preparative HPLC was carried out on an Aglient 1260 instrument equipped with MWD detector (Aglient 1260, MWDVL, USA) and a MG II column (5 μm, 20 mm I. D. × 250 mm; SHISEIDO, JPN) with a weak base mobile phase (53% methanol-water (v/v) with 0.01% diethylamine). Preparative HPLC gave the compounds **3** (3.0 mg, 31%).

R_f = 0.52 (DCM/MeOH = 15/1);

[α]20 D = + 139 (c = 0.3, MeOH) {lit. [α] + 242 (c = 0.3, MeOH)}[8];

IR (film) 2926, 2854, 1558, 1456, 1259, 1026 cm^{-1};

^{1}H NMR (400 MHz, $CDCl_3$) δ 7.16 (s, 1H), 7.11 (d, *J* = 10.4 Hz, 1H), 6.91 (s, 1H), 6.69 (d, *J* = 10.4 Hz, 1H), 5.25 (s, 1H), 4.30 (d, *J* = 1.6 Hz 1H), 4.14 (s, 3H), 3.92 (s, 3H), 3.03 (s, 3H), 2.49 – 2.38 (m, 1H), 2.08 – 1.98 (m, 1H), 1.86 (d, *J* = 11.2 Hz, 1H), 1.69 (s, 1H);

^{13}C NMR (101 MHz, $CDCl_3$) δ 180.2, 179.4, 164.5, 163.0, 145.8, 140.5, 137.4, 133.6, 130.6, 129.9, 111.7, 90.4, 65.6, 60.2, 56.3, 51.0, 37.0, 26.3, 23.4;

HRMS (ESI) Calcd for $C_{19}H_{20}NO_5Na$ $[M+Na]^+$: 364.1155; Found: 364.1157.

Table S1 Compared NMR data [CDC1$_3$] between the synthetic (+)-demecolcinone and the isolated natural product.

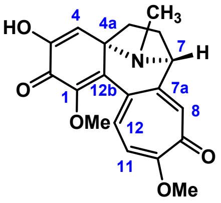

	^{1}H & ppm (J)			^{13}C & ppm		
Carbon	isolated (500M)	synthesized (400M)	error (iso. - syn.)	isolated (125M)	synthesized (101M)	error (iso. - syn.)
1				140.5	140.5	0
2				180.2	180.2	0
3				133.6	133.6	0
4	5.25 s	5.25 s	0	90.4	90.4	0
4a				51.0	51.0	0
5	1.69 m/2.43 m	1.69 m/2.49 m	0	26.3	26.3	0
6	1.85 m/2.04 m	1.86 m/2.08 m	0	23.4	23.4	0
7	4.31 dd (2.2)	4.30 d (1.6)	0.01	65.6	65.6	0
7a				145.8	145.8	0
8	7.16 s	7.16 s	0	130.6	130.6	0
9				179.4	179.4	0
10				164.5	164.5	0
11	6.66 d (11)	6.69 d (10.4)	-0.03	111.6	111.7	-0.1
12	7.11 d (11)	7.11 d (10.4)	0	129.9	129.9	0
12a				137.4	137.4	0
12b				163.0	163.0	0
1-OCH3	4.14 s	4.14 s	0	60.1	60.0	0.1
10-OCH3	3.93 s	3.92 s	0.01	56.3	56.3	0
NCH3	3.03 s	3.03 s	0	37.0	37.0	0

Synthesis of 25

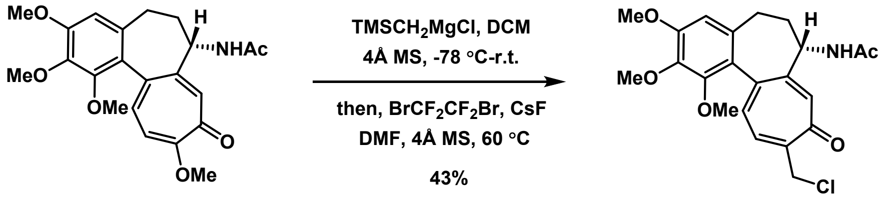

colchicine (1) **25**

Colchicine (50 mg, 0.13 mmol) and freshly dried 4Å molecular sieve (50 mg) were added in a 25mL flask. The solids were dissolved in dried DCM (5 mL) at -78 ºC and then $TMSCH_2MgCl$ (commercially available, 0.38 mL, 1M in Et_2O, 0.38 mmol) was added dropwise at the same temperature. After stirring for 5 minutes, the reaction mixture (orange turbid solution) was transferred to room temperature and stirred for additional 4 hours. When the starting material disappeared, the solution was diluted with dried DCM (5 mL) and filtered through Celite® quickly and washed with DCM until all the orange mud disappeared. The filtrate was concentrated in *vacuo*

quickly to give a solid which was dissolved in dried DMF (5 mL) and then freshly dried 4Å molecular sieve (50 mg) was added. After the solution was stirred at room temperature for 5 minutes, $BrCF_2CF_2Br$ (97 mg, 0.05 mL, 0.38 mmol) and CsF (57 mg, 0.38 mmol) were added to the solution in sequence. The lutescens solution was transferred to 60 ºC and stirred overnight. The reaction was diluted with DCM (5 mL) and washed with water (2 x 5 mL). The layers were separated and the aqueous layer was extracted with DCM (2 x 5 mL). The combined organic layers were dried over Na_2SO_4, filtered, and concentrated under reduced pressure. The crude residue was purified by column chromatography (DCM/MeOH = 100/1-30/1) to afford alkene **25** (23 mg, 43% yield) as a yellow solid.

R_f = 0.55 (DCM/MeOH = 10/1);

^{1}H NMR (500 MHz, $CDCl_3$) δ 7.74 (d, J = 9.8 Hz, 1H), 7.53 (s, 1H), 7.37 (d, J = 9.8 Hz, 1H), 7.11 (d, J = 7.2 Hz, 1H), 6.53 (s, 1H), 4.73 – 4.59 (m, 3H), 3.94 (s, 3H), 3.90 (s, 3H), 3.68 (s, 3H), 2.56 (dd, J = 13.7, 6.4 Hz, 1H), 2.41 (dt, J = 13.6, 6.8 Hz, 1H), 2.28 – 2.23 (m, 1H), 2.02 (s, 3H), 1.91 (dt, J = 12.0, 6.0 Hz, 1H);

^{13}C NMR (126 MHz, $CDCl_3$) δ 184.16, 169.88, 154.20, 152.87, 151.36, 147.07, 144.86, 141.75, 137.12, 136.28, 134.64, 134.25, 125.03, 107.46, 61.79, 61.42, 56.14, 52.29, 44.82, 36.36, 29.85, 23.00.

IR (ν cm^{-1}) 2934, 2987, 1710, 1553, 1138, 1091, 901;

HRMS-ESI (*m/z*) $[M+H]^+$ calcd for $C_{22}H_{25}O_5NCl$ 418.1416, found 418.1415.

The process from 1 to 25 involved a series of sequential reactions as follow.

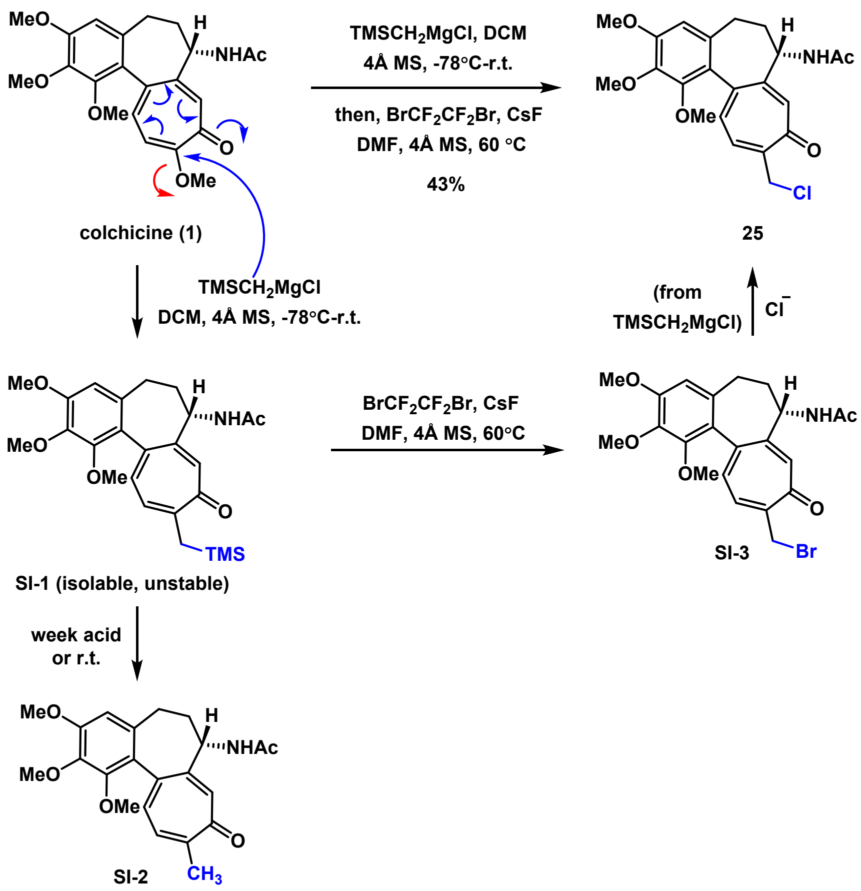

Compound SI-1:

R_f = 0.5 (DCM/MeOH = 10/1);

^{1}H NMR (400 MHz, $CDCl_3$) δ 7.41 (d, J = 7.2 Hz, 1H), 7.33 (s, 1H), 7.25 (d, 1H), 7.17 (d, J = 9.9 Hz, 1H), 6.52 (s, 1H), 4.64 (dd, J = 11.9, 6.7 Hz, 1H), 3.93 (s, 3H), 3.89 (s, 3H), 3.65 (s, 3H), 2.52 (dd, J = 13.3, 6.6 Hz, 1H), 2.42 (dt, J = 13.4, 6.4 Hz, 1H), 2.34 (s, 2H), 2.23 (dt, J = 12.6, 6.4 Hz, 1H), 1.98 (s, 3H), 1.85 (dt, J = 11.6, 5.9 Hz, 1H), 0.05 (s, 9H);

^{13}C NMR (101 MHz, $CDCl_3$) δ 185.32, 169.69, 155.58, 153.50, 151.38, 150.09, 141.66, 139.55, 135.85, 134.24, 132.99, 131.93, 125.77, 107.29, 61.52, 61.41, 56.13, 51.99, 36.57, 29.99, 27.62, 23.00, -1.03;

HRMS-ESI (*m/z*) $[M+H]^+$ calcd for $C_{25}H_{34}O_5NSi$ 456.2201, found 456.2196.

Compound SI-2:

R_f = 0.45 (DCM/MeOH = 10/1);

^{1}H NMR (500 MHz, $CDCl_3$) δ 7.39 (dd, J = 9.6, 1.2 Hz, 1H), 7.25 (s, 1H), 7.16 (d, J = 9.6 Hz, 1H), 6.59 (d, J = 7.0 Hz, 1H), 6.52 (s, 1H), 4.61 (dt, J = 11.7, 6.8 Hz, 1H), 3.93 (s, 3H), 3.90 (s, 3H), 3.66 (s, 3H), 2.56 – 2.50 (m, 1H), 2.44 (td,

J = 13.2, 6.8 Hz, 1H), 2.32 (d, J = 1.2 Hz, 3H), 2.22 (tt, J = 12.7, 5.9 Hz, 1H), 1.99 (s, 3H), 1.79 (tdd, J = 11.9, 6.8, 1.3 Hz, 1H).

HRMS-ESI (*m/z*) $[M+H]^+$ calcd for $C_{22}H_{26}O_5N$ 384.1805, found 384.1799.

Compound SI-3:

$\boldsymbol{R_f}$ = 0.56 (DCM/MeOH = 10/1);

^{1}H NMR (500 MHz, $CDCl_3$) δ 7.63 (d, J = 9.8 Hz, 1H), 7.50 (s, 1H), 7.37 (d, J = 7.2 Hz, 1H), 7.24 (d, J = 9.7 Hz, 1H), 6.53 (s, 1H), 4.68 – 4.60 (m, 1H), 4.56 (d, J = 9.6 Hz, 1H), 4.49 (d, J = 9.6 Hz, 1H), 3.93 (s, 3H), 3.90 (s, 3H), 3.68 (s, 3H), 2.55 (dd, J = 13.7, 6.3 Hz, 1H), 2.42 (td, J = 13.3, 6.7 Hz, 1H), 2.24 (dq, J = 12.6, 6.4 Hz, 1H), 2.01 (s, 3H), 1.88 (dt, J = 11.8, 5.9 Hz, 1H).

^{13}C NMR (126 MHz, $CDCl_3$) δ 184.06, 169.88, 154.10, 152.06, 151.34, 147.74, 144.81, 141.70, 137.67, 135.76, 135.13, 134.24, 125.12, 107.41, 61.80, 61.41, 56.13, 52.18, 36.21, 32.36, 29.88, 22.99.

HRMS-ESI (*m/z*) $[M+H]^+$ calcd for $C_{22}H_{25}O_5NBr$ 462.0911, found 462.0909.

Synthesis of (–)-(a*R*,7*S*)-metacolchicine (26)

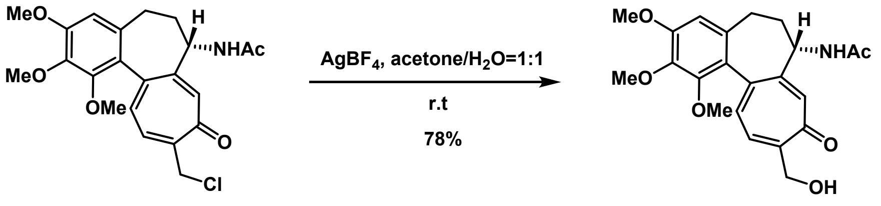

25

(–)-(aR,7S)-metacolchicine (26)

Compound **25** (20 mg, 0.05 mmol) was dissolved in a solution of acetone and water (1:1, 5 mL) at room temperature and then $AgBF_4$ (48 g, 0.25 mmol) was added at the same temperature. After stirred for 10 hours at room temperature, the reaction was diluted with DCM (3 mL) and washed with water (2 x 5mL). The layers were separated and the aqueous layer was extracted with DCM (2 x 5 mL). The combined organic layers were dried over Na_2SO_4, filtered, and concentrated under reduced pressure. The crude residue was purified by column chromatography (DCM/MeOH = 50/1-20/1) to afford alkene 25 (15.5 mg, 78% yield) as a yellow solid.

$\boldsymbol{R_f}$ = 0.3 (DCM/MeOH = 10/1);

[α]26 D = - 109 (c = 0.1, $CHCl_3$);

^{1}H NMR (500 MHz, $CDCl_3$) δ 7.67 (d, J = 9.8 Hz, 1H), 7.52 (s, 1H), 7.43 (d, J = 9.7 Hz, 1H), 7.34 (d, J = 6.9 Hz, 1H), 6.54 (s, 1H), 4.77 – 4.55 (m, 3H), 3.93 (s, 3H), 3.90 (s, 3H), 3.66 (s, 3H), 2.55 (dd, J = 13.6, 6.3 Hz, 1H), 2.37 (td, J =

13.3, 6.8 Hz, 1H), 2.25 (dt, J = 12.7, 6.2 Hz, 1H), 2.01 (s, 3H), 1.91 (td, J = 12.0, 6.8 Hz, 1H);

^{13}C NMR (126 MHz, $CDCl_3$) δ 185.68, 170.00, 154.13, 153.68, 151.28, 150.95, 144.34, 141.69, 137.56, 135.36, 134.22, 134.03, 125.08, 107.42, 65.01, 61.72, 61.42, 56.14, 52.50, 36.41, 29.81, 22.95;

IR (ν cm^{-1}) 3277, 2924, 1750, 1613, 1550, 1134, 884;

HRMS-ESI (*m/z*) $[M+H]^+$ calcd for $C_{22}H_{26}O_6N$ 400.1755, found 400.1757.

Synthesis of 27

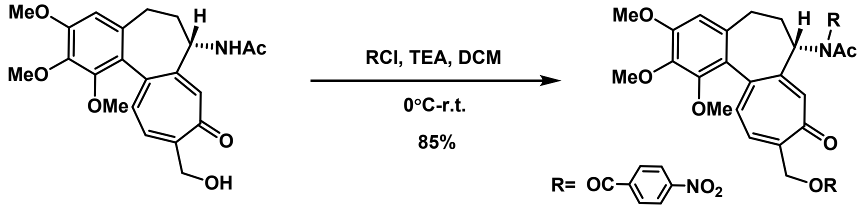

(–)-(aR,7S)-metacolchicine (26) **27**

Compound **26** (12 mg, 0.03 mmol) was dissolved in DCM (5 mL) at room temperature and then the solution was transferred to 0 ºC. After stirred for 5 minutes, triethylamine (0.01 mL, 0.08 mmol) and p-nitrobenzoyl chloride (15 mg, 0.08 mmol) were added to the solution in sequence. After stirred at the same temperature for 10 minutes, the reaction was transferred to room temperature and stirred for additional 8 hours. The reaction was quenched by saturated aqueous NH_4Cl (4 mL) and washed by water (5 mL). The layers were separated and the aqueous layer was extracted with DCM (2 x 5 mL). The combined organic layers were dried over Na_2SO_4, filtered, and concentrated under reduced pressure. The crude residue was purified by column chromatography (Hexane/EA = 5/1-1/1) to afford **27** (17 mg, 85% yield) as a yellow solid.

R_f = 0.2 (Hexane/EA = 2/1);

^{1}H NMR (400 MHz, $CDCl_3$) δ 8.41 – 8.20 (m, 6H), 7.94 (d, J = 8.4 Hz, 2H), 7.63 (s, 1H), 7.46 (d, J = 9.7 Hz, 1H), 7.23 (s, 1H), 5.53 – 5.40 (m, 2H), 5.34 (dd, J = 12.1, 5.9 Hz, 1H), 3.94 (s, 3H), 3.92 (s, 3H), 3.74 (s, 3H), 2.59 (ddt, J = 30.4, 13.5, 6.4 Hz, 3H), 1.84 (s, 3H), 0.85 (dd, J = 17.7, 6.8 Hz, 1H);

^{13}C NMR (126 MHz, $CDCl_3$) δ 184.75, 164.23, 154.08, 151.32, 150.71, 150.57, 148.80, 142.81, 141.86, 141.42, 137.11, 135.35, 135.23, 133.91, 133.13, 130.97, 130.24, 125.58, 124.45, 123.68, 107.35, 65.23, 61.69, 61.34, 60.15, 56.11, 31.87, 29.99, 14.21;

Synthesis of colchiceine (28)

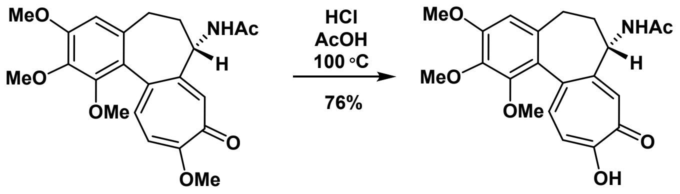

colchicine (1) colchiceine (28)

Compound **1** (50 mg, 1.25 mmol) was dissolved in HOAc (0.5 mL), and then aqueous HCl (0.1N, 3 mL) was added. The clear solution was heated at 90-100 °C for 2 h. After the solution was cooled to room temperature, solid Na_2CO_3 was added until the pH reached 6-7. The mixture was extracted with $CHCl_3$, (3 x 5 mL), washed with brine (1 mL), and then was dried over by $MgSO_4$. The solvent was removed under reduced pressure and gave a yellow solid, which was crystallized from MeOH/H_2O to give **28**[11] (34.4 mg, 76%) as a light yellow solid.

***R**$_f$* = 0.15 (DCM/MeOH = 20/1);

[α]20 D = -206.1 (c = 1.0, $CHCl_3$);

IR (ν cm^{-1}) 2938, 1611, 1491, 1402, 1275, 1136, 1011, 845;

^{1}H NMR (500 MHz, $CDCl_3$) δ 7.61 (d, *J* = 11.6 Hz, 1H), 7.60 (s, 1H), 7.35 (d, *J* = 11.6 Hz, 1H), 6.66 (d, *J* = 5.7 Hz, 1H), 6.58 (s, 1H), 4.72 – 4.65 (m, 1H), 3.96 (s, 3H), 3.93 (s, 3H), 3.65 (s, 3H), 2.59 – 2.48 (m, 1H), 2.41 – 2.27 (m, 2H), 2.05 (s, 3H), 1.98 – 1.90 (m, 1H);

^{13}C NMR (126 MHz, $CDCl_3$) δ 170.4, 170.0, 169.8, 153.7, 151.01, 150.99, 141.7, 141.5, 136.2, 134.3, 126.0, 122.6, 119.2, 107.4, 61.50, 61.45, 56.1, 52.8, 37.7, 29.8, 23.0;

HRMS (ESI) calcd for $C_{21}H_{24}O_6N$ $[M+H]^+$: 386.1559, found 386.1592.

Synthesis of metacolchicine (4)

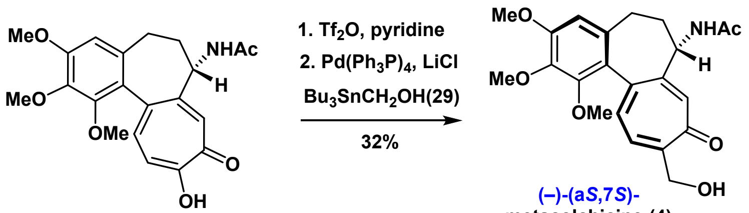

colchiceine (28) metacolchicine (4)

Triflic anhydride (8.5 uL, 0.05 mmol) was added dropwise to a stirred solution of **28** (10 mg, 0.026 mmol) in pyridine (0.4 mL) at 0 °C. The reaction mixture was allowed to warm to room temperature and stirred for 1 h. Then the mixture was diluted with ether (3 mL) and washed with 1M HCl (0.4 mL), water (0.5 mL) and brine (0.5 mL) sequentially. Organic layer was dried over Na_2SO_4, filtered, concentrated and the residue was purified by flash column chromatography (DCM: MeOH = 40:1 $\rightarrow$ 30:1) to yield the triflates mixture as a yellow oil, which

was used in the next step.

To a solution of the above triflates mixture in THF (3 mL) at room temperature was added $Pd(PPh_3)_4$ (5.7 mg, 0.005mmol) and LiCl (2.3 mg, 0.05 mmol) sequentially. The mixture was stirred at room temperature for 10 minutes before addition of Bu_3SnCH_2OH (**29**, 17 mg, 0.05 mmol) in THF (1 mL). Then the mixture was heated to 65 ^oC and stirred overnight. The solvent was evaporated under reduced pressure to give the crude product, which was purified by silica gel column chromatography ($CHCl_3$: MeOH = 30:1 $\rightarrow$ 20:1 $\rightarrow$ 15:1) to afforded metacolchicine **4** (3.3 mg, 32%).

$\boldsymbol{R_f}$ = 0.2 (DCM/MeOH = 15/1);

[α]$^{20}{}_D$ = -167.3 (c = 1.0, $CHCl_3$);

^{1}H NMR (400 MHz, $CDCl_3$) δ 7.51 (d, *J* = 9.7 Hz, 1H), 7.34 – 7.29 (m, 2H), 6.88 (s, 1H), 6.53 (s, 1H), 4.72 (d, *J* = 14.5 Hz, 1H), 4.65 – 4.56 (m, 2H), 3.94 (s, 3H), 3.91 (s, 3H), 3.67 (s, 3H), 3.55 (s, 1H), 2.59 – 2.52 (m, 1H), 2.47 – 2.38 (m, 1H), 2.30 – 2.20 (m, 1H), 2.00 (s, 3H), 1.86 – 1.78 (m, 1H);

^{13}C NMR (101 MHz, $CDCl_3$) δ 186.9, 169.6, 153.9, 151.7, 151.4, 150.5, 143.2, 141.8, 136.5, 134.4, 134.0, 125.3, 107.4, 65.8, 61.7, 61.4, 56.1, 52.2, 36.4, 29.8, 23.0;

IR (ν cm^{-1}) 3281, 2932, 1748, 1653, 1558, 1138, 1094, 922, 733;

HRMS-ESI (*m/z*) $[M+H]^+$ calcd for $C_{22}H_{26}O_6N$ 400.1755, found 400.1744.

Table S2 Compared NMR data [$CDC1_3$] between the synthetic (a*S*,7*S*)-metacolchicine (**4**), (a*R*,7*S*)-metacolchicine (**26**) and the isolated natural product.

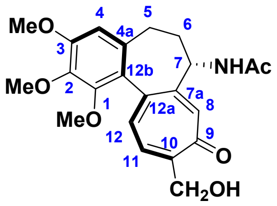

(a*S*,7*S*)-metacolchicine (4)

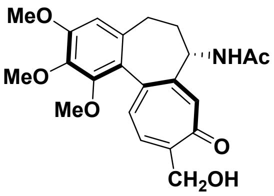

(a*R*,7*S*)-metacolchicine (26)

Carbon	^{1}H NMR & ppm (J) Natural (500 MHz)	(a*S*,7*S*)-4 (400 MHz)	Error[a]	(a*R*,7*S*)-26 (500 MHz)	Error[a]	^{13}C NMR & ppm Natural (125 MHz)	(a*S*,7*S*)-4 (100 MHz)	Erro[a]	(a*R*,7*S*)-6 (125 MHz)	Erro[a]
1	-	-				151.4	151.4	0	151.3	-0.1
2	-	-				141.8	141.8	0	141.7	-0.1
3	-	-				153.9	153.9	0	154.2	+0.3
4	6.53 (s)	6.53 (s)	0	6.54 (s)	+0.01	107.4	107.4	0	107.4	0
4a	-	-				134.0	134.0	0	134.2	+0.2
5α	2.43 (m)	2.47 – 2.38 (m)	0	2.37 (td)	-0.06	29.8	29.8	0	29.8	0
β	2.56 (m)	2.59 – 2.52 (m)	0	2.55 (dd)	-0.01					
6a	1.81 (m)	1.86 – 1.78 (m)	0	1.91 (td)	+0.10	36.4	36.4	0	36.4	0
β	2.24 (m)	2.30 – 2.20 (m)	0	2.26 (td)	+0.02					
7	4.60 (m)	4.65 – 4.56 (m)	0	4.67–4.56 (m)	0	52.2	52.2	0	52.5	+0.3
7a	-	-				151.7	151.7	0	153.7	+2.0
8	7.30 (s)	7.34 – 7.29 (s)	0	7.52 (s)	+0.22	134.4	134.4	0	134.1	-0.3
9	-	-				186.9	186.9	0	185.7	-1.2
10	-	-				150.5	150.5	0	151.0	+0.5
11	7.50 (d, 9.5)	7.51 (d, 9.7)	0.01	7.67 (d, 9.8)	+0.17	134.4	134.4	0	135.4	+1.0
12	7.31 (d, 9.5)	7.34 – 7.29 (d)	0	7.43 (d)	+0.12	136.4	136.5	0.1	137.6	+1.2
12a	-	-				143.2	143.2	0	144.4	+1.2
12b	-	-				125.3	125.3	0	125.1	-0.2
NH	6.83 (br-d, 6.7)	6.88 (br-s)	0.05	7.34 (br-d)	+0.51	-				
$N\underline{C}OCH_3$	-	-				169.5	169.6	0.1	170.0	+0.5
$NCO\underline{C}H_3$	2.00 (3H, s)	2.00 (s)	0	2.01	+0.01	23.0	23.0	0	23.0	0
10-$\underline{C}H_2OH$	4.72 (dd)	4.72 (d, 14.5)	0	4.72 (d, 15.2)	0	65.8	65.8	0	65.0	-0.8
	4.60 (dd)	4.65 – 4.56 (dd)	0	4.67–4.56 (m)	0	-				
10-$CH_2O\underline{H}$	3.51 (br-dd)	3.55 (br-s)	0.04	2.94 (br-s)	-0.57	-				
1-OCH_3	3.67 (3H, s)	3.67 (s,)	0	3.66 (s)	-0.01	61.6	61.7	0.1	61.7	+0.1
2-OCH_3	3.94 (3H, s)	3.94 (s)	0	3.93 (s)	-0.01	61.4	61.4	0	61.4	0
3-OCH_3	3.91 (3H, s)	3.91 (s)	0	3.90 (s)	-0.01	56.1	56.1	0	56.2	+0.1

a: Compared with the data of natural product

III. HPLC Analysis of *ee* Value

(a)

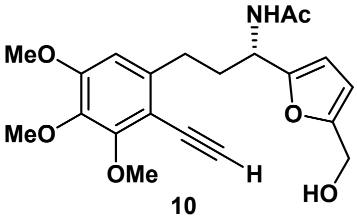

10

HPLC analysis: Daicel Chiralpak AD-H column; hexane/*i*-propanol = 90:10, 0.8 mL/min, λ = 254 nm; t_R(minor) = 20.9 min, t_R(major) = 23.6 min. > 99% *ee*.

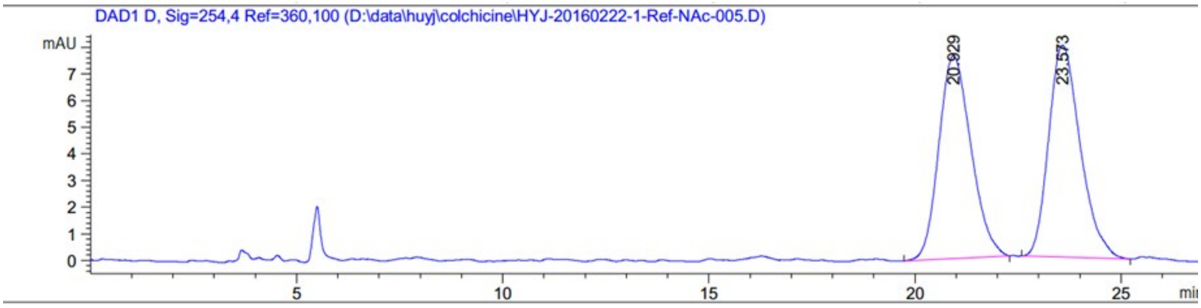

Peak #	RetTime [min]	Type	Width [min]	Area [mAU*s]	Height [mAU]	Area %
1	20.929	BB	0.8221	413.57431	7.60897	49.4017
2	23.573	BB	0.8019	423.59146	7.94638	50.5983

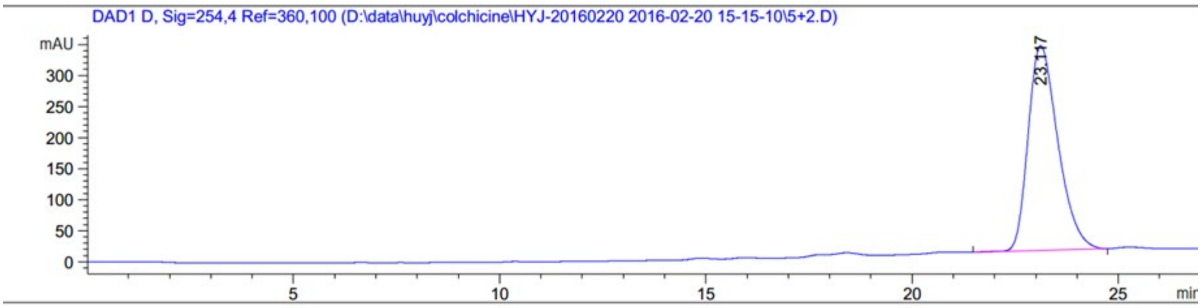

Peak #	RetTime [min]	Type	Width [min]	Area [mAU*s]	Height [mAU]	Area %
1	23.117	BB	0.7740	1.65770e4	330.05228	100.0000

(b)

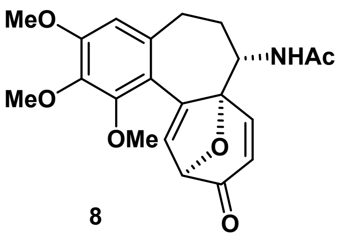

8

HPLC analysis: Daicel Chiralpak AD-H column; hexane/*i*-propanol = 75:25, 1 mL/min, λ = 254 nm; t_R(major) = 4.6 min, t_R(minor) = 5.6 min. > 99% *ee*.

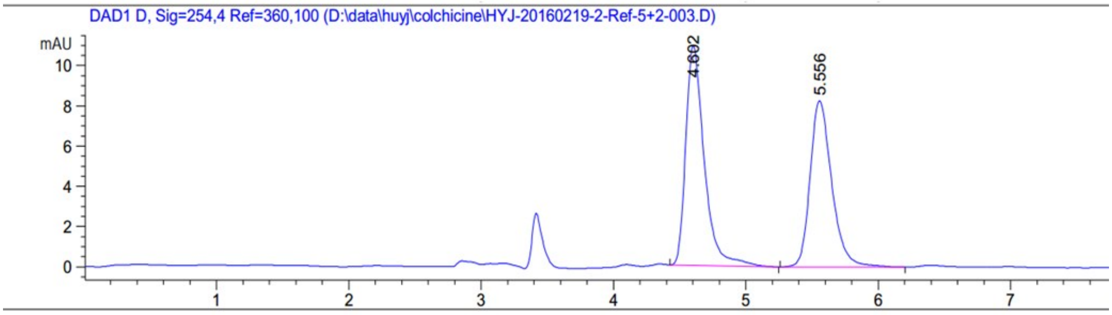

Peak #	RetTime [min]	Type	Width [min]	Area [mAU*s]	Height [mAU]	Area %
1	4.602	BB	0.1456	106.28229	10.91946	53.0118
2	5.556	BB	0.1726	94.20567	8.28594	46.9882

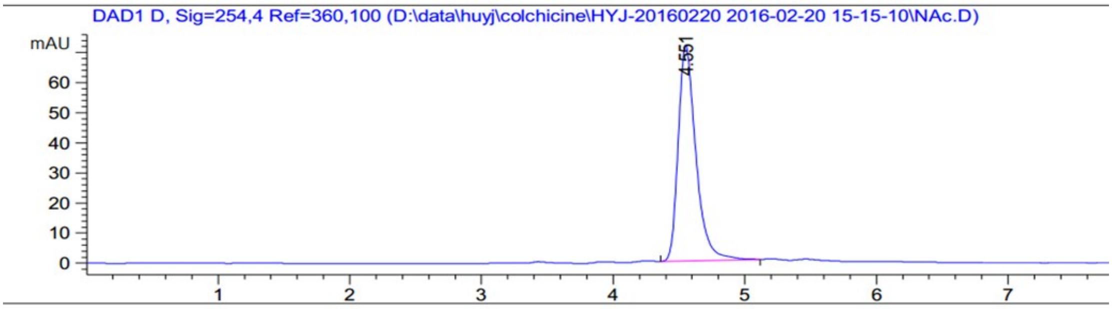

Peak #	RetTime [min]	Type	Width [min]	Area [mAU*s]	Height [mAU]	Area %
1	4.551	BB	0.1406	667.80676	71.76360	100.0000

(c)

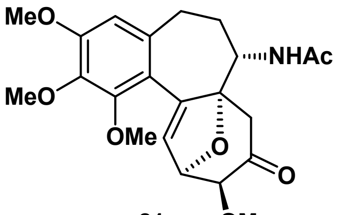

21

HPLC analysis: Daicel Chiralpak AD-H column; hexane/*i*-propanol = 80:20, 1 mL/min, λ = 260 nm; t_R(minor) = 7.4 min, t_R(major) = 11.3 min. > 99% *ee*.

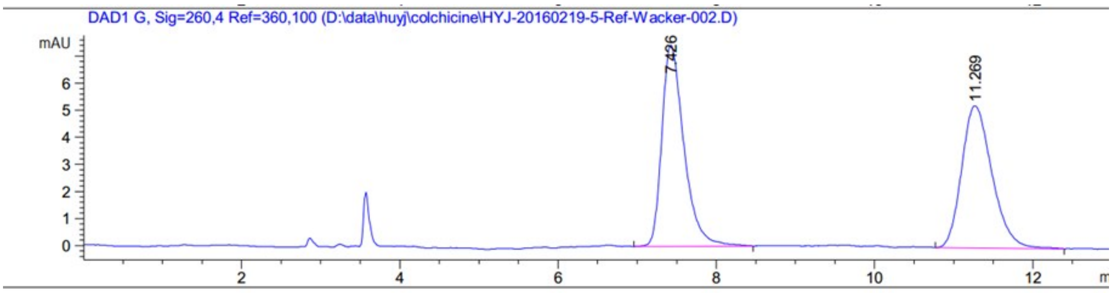

Peak #	RetTime [min]	Type	Width [min]	Area [mAU*s]	Height [mAU]	Area %
1	7.426	BB	0.2945	144.26382	7.42947	50.7805
2	11.269	BB	0.4066	139.82912	5.25265	49.2195

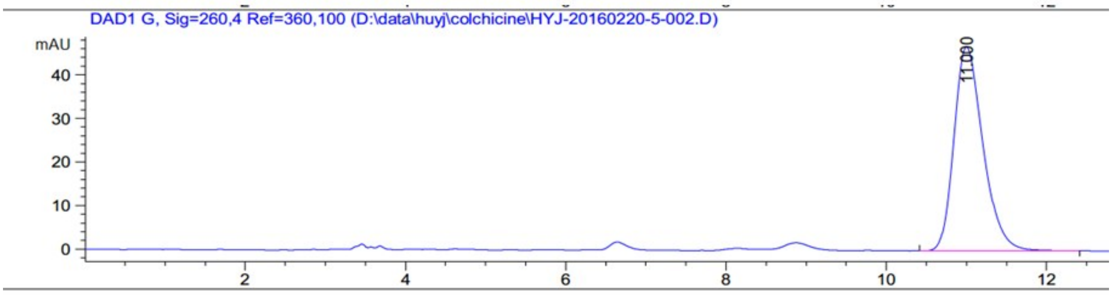

Peak #	RetTime [min]	Type	Width [min]	Area [mAU*s]	Height [mAU]	Area %
1	11.000	BB	0.3891	1185.07092	46.84715	100.0000

(d)

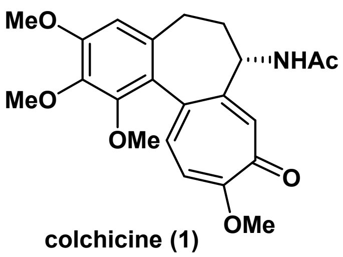

colchicine (1)

HPLC analysis: Daicel Chiralpak AD-H column; hexane/*i*-propanol = 70:30, 1 mL/min, λ = 254 nm; t_R(major) = 4.9 min, t_R(major) = 7.0 min. > 99% *ee*.

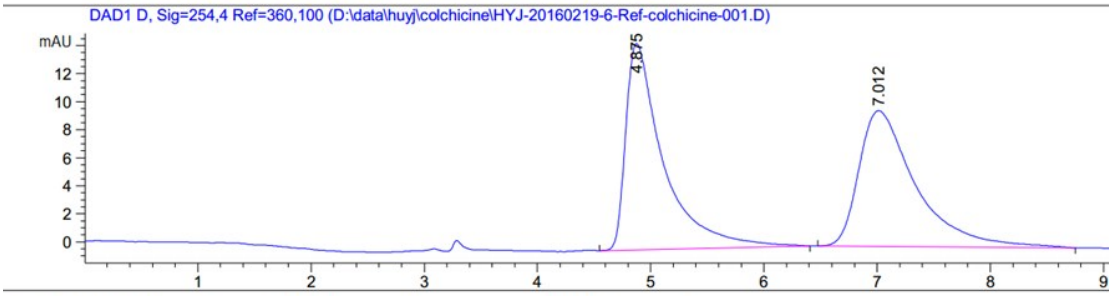

Peak #	RetTime [min]	Type	Width [min]	Area [mAU*s]	Height [mAU]	Area %
1	4.875	BB	0.3311	339.20428	14.70766	50.2097
2	7.012	BB	0.5098	336.37128	9.66964	49.7903

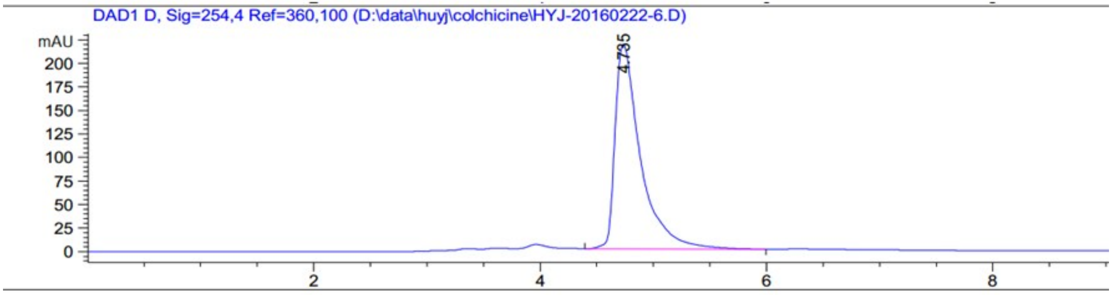

Peak #	RetTime [min]	Type	Width [min]	Area [mAU*s]	Height [mAU]	Area %
1	4.735	BB	0.2301	3401.63550	217.92906	100.0000

IV. X-ray Crystallographic Data

X-Ray data for 8:

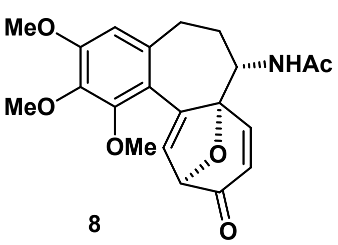

8

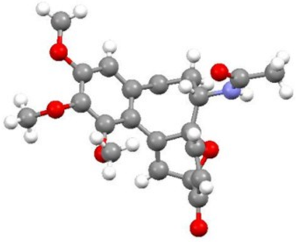

Crystal data and structure refinement for **8** (CCDC 1547674).

Empirical formula	C21 H22 N O6
Formula weight	384.40
Temperature	566(2) K
Wavelength	0.71073 A
Crystal system, space group	Monoclinic, P2(1)
Unit cell dimensions	a = 8.760(3) A alpha = 90 deg.
	b = 13.957(5) A beta = 90 deg.
	c = 16.322(5) A gamma = 90deg.
Volume	1995.7(11) A^3
Z, Calculated density	4, 1.279 Mg/m^3
Absorption coefficient	0.094 mm^-1
F(000)	812
Crystal size	0.300 x 0.200 x 0.150 mm
Theta range for data collection	1.92 to 25.34 deg.
Limiting indices	-10<=h<=10, -16<=k<=16, -18<=l<=19
Reflections collected / unique	31078 / 3624 [R(int) = 0.1949]
Completeness to theta = 25.34	99.6 %
Refinement method	Full-matrix least-squares on F^2
Data / restraints / parameters	3624 / 0 / 254
Goodness-of-fit on F^2	1.011
Final R indices [I>2sigma(I)]	R1 = 0.0860, wR2 = 0.2598
R indices (all data)	R1 = 0.0950, wR2 = 0.2884
Absolute structure parameter	3(2)

Extinction coefficient 0.38(4)

Largest diff. peak and hole 0.412 and -0.219 e.A^-3

X-Ray data for 27:

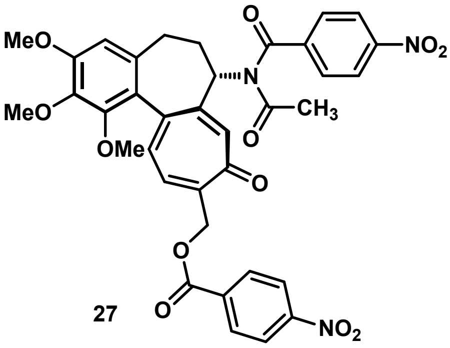

27

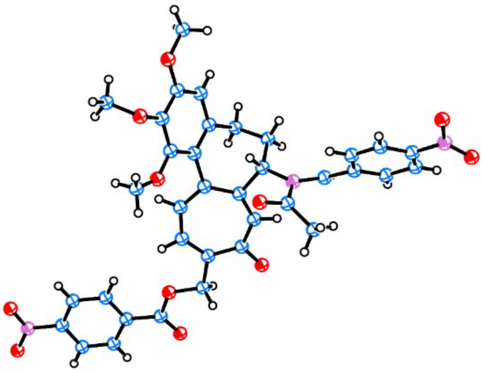

Crystal data and structure refinement for **27** (CCDC 1524362).

Bond precision: C-C = 0.0144 A Wavelength=0.71073

Cell:	a=13.869(9)	b=9.736(8)	c=14.969(11)
	alpha=90	beta=105.103(18)	gamma=90

Temperature: 293 K

Volume	1951(3)
Space group	P 21
Hall group	P 2yb
Moiety formula	C36 H31 N3 O12, C4 H5 O2
Sum formula	C40 H36 N3 O14
Mr	782.72
Dx,g cm-3	1.332
Z	2
Mu (mm-1)	0.102
F000	818.0
F000'	818.48
h,k,lmax	16,11,18
Nref	7161[3809]
Tmin,Tmax	0.980,0.980
Tmin'	0.980

Correction method= # Reported T Limits: Tmin=0.593 Tmax=1.000 AbsCorr = MULTI-SCAN

Data completeness= 1.60/0.85 Theta(max)= 25.361

R(reflections)= 0.0992(3344) wR2(reflections)= 0.3148(6095)

S = 1.023 Npar= 515

V. ^{1}H and ^{13}C NMR Spectra

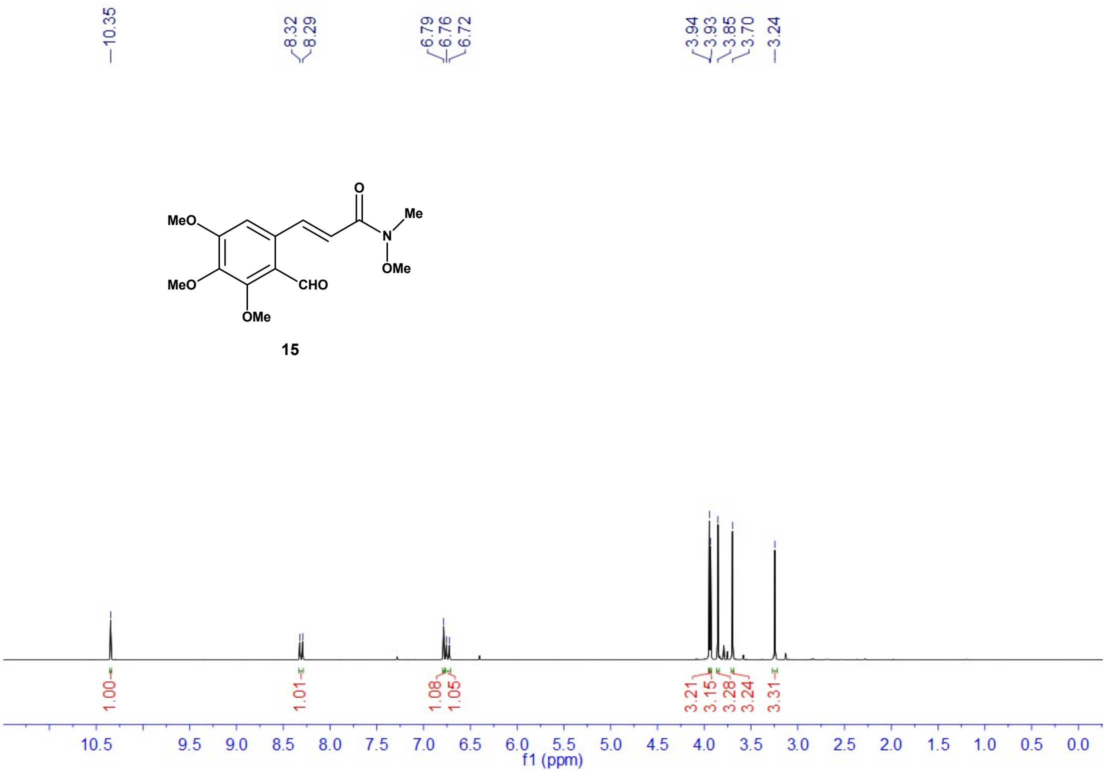

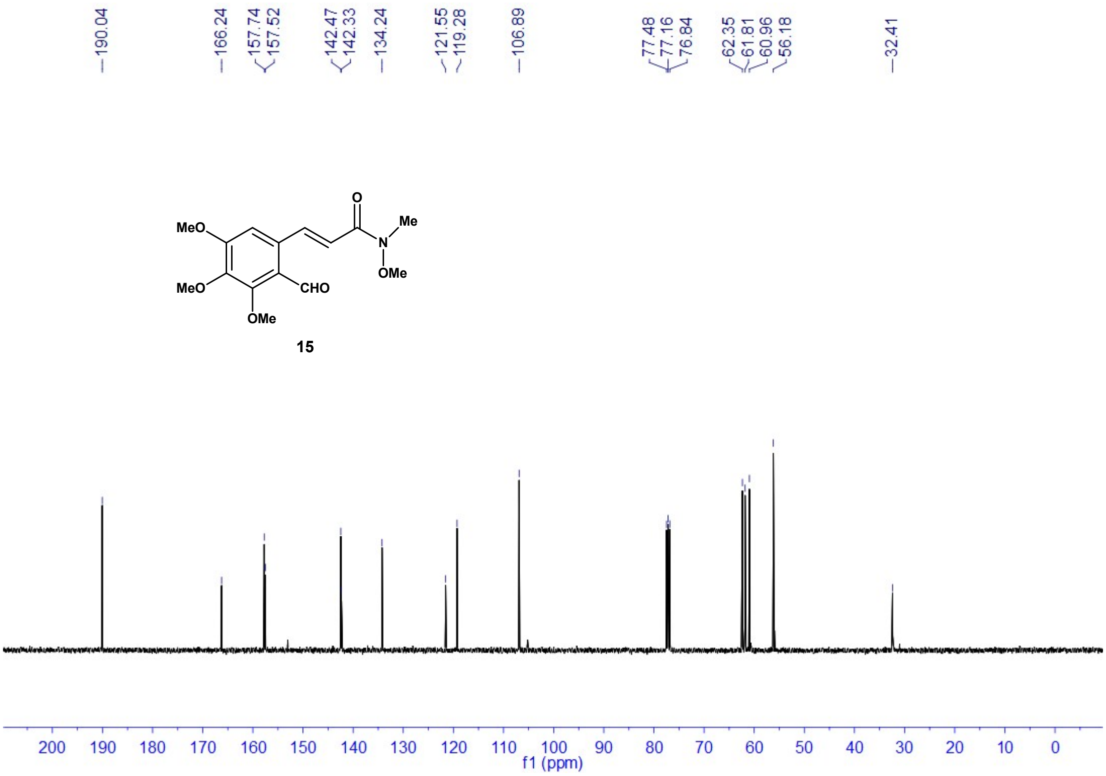

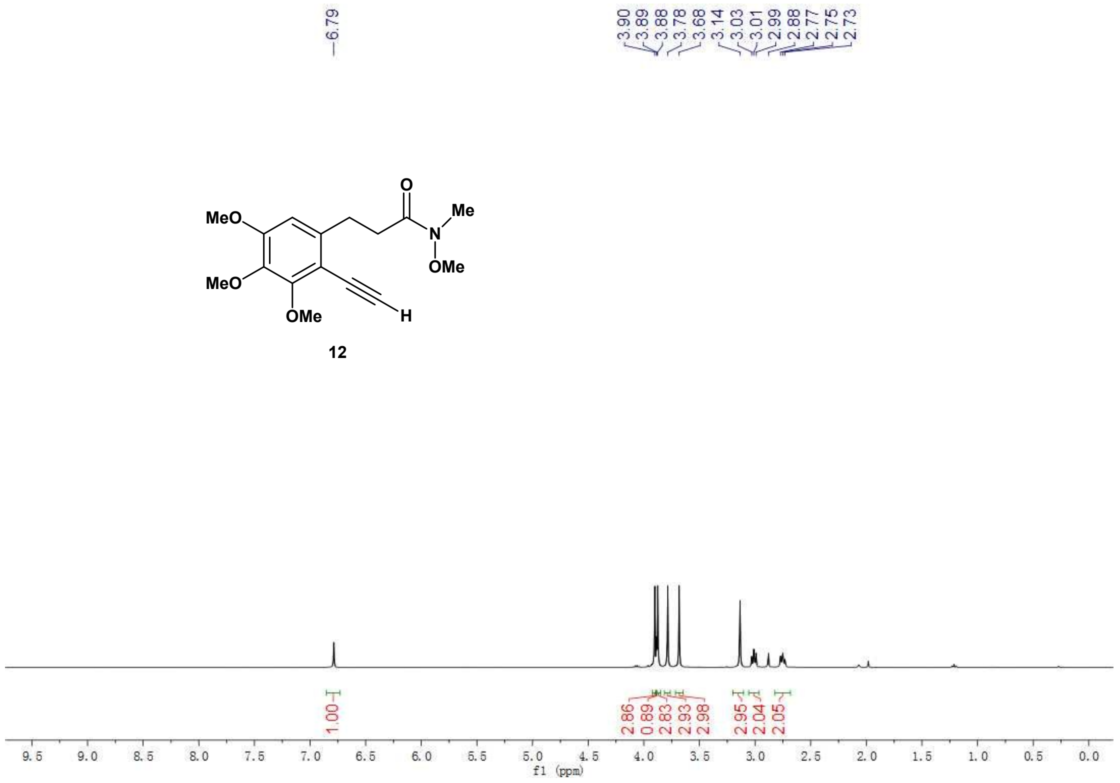
6.79
3.90
3.89
3.88
3.78
3.68
3.14
3.03
3.01
2.99
2.88
2.77
2.75
2.73
MeO
MeO
OMe
O
N
Me
OMe
H
12
1.00
2.86
0.89
2.83
2.93
2.98
2.95
2.04
2.05
f1 (ppm)

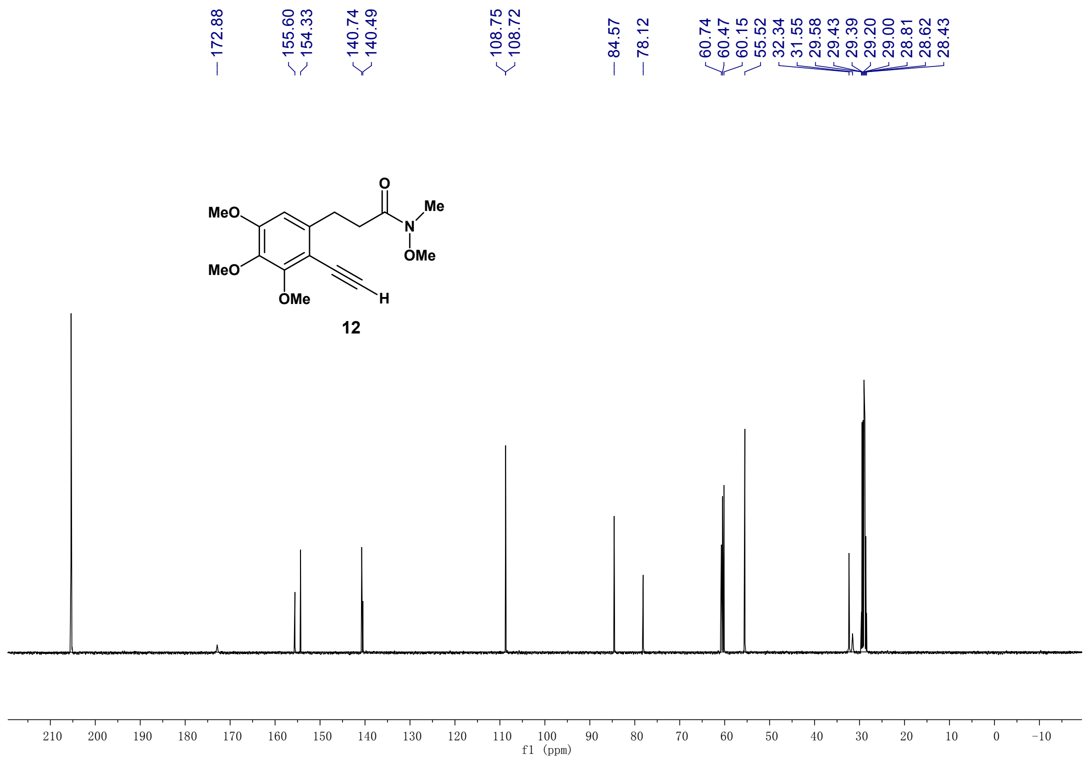
172.88
155.60
154.33
140.74
140.49
108.75
108.72
84.57
78.12
60.74
60.47
60.15
55.52
32.34
31.55
29.58
29.43
29.39
29.20
29.00
28.81
28.62
28.43
MeO
MeO
OMe
O
N
Me
OMe
H
12
f1 (ppm)

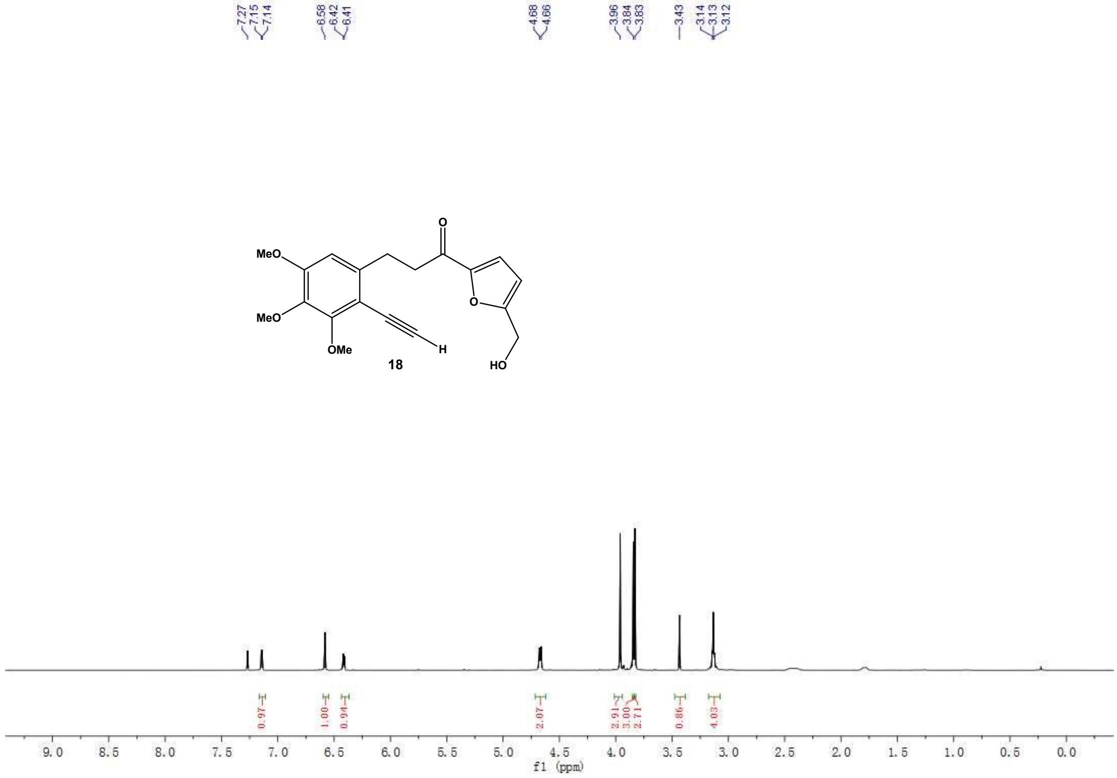
7.27
7.15
7.14
6.58
6.42
6.41
4.68
4.66
3.96
3.84
3.83
3.43
3.14
3.13
3.12
MeO
MeO
OMe
O
O
H
HO
18
0.97
1.00
0.94
2.07
2.91
3.00
2.71
0.86
4.03
f1 (ppm)

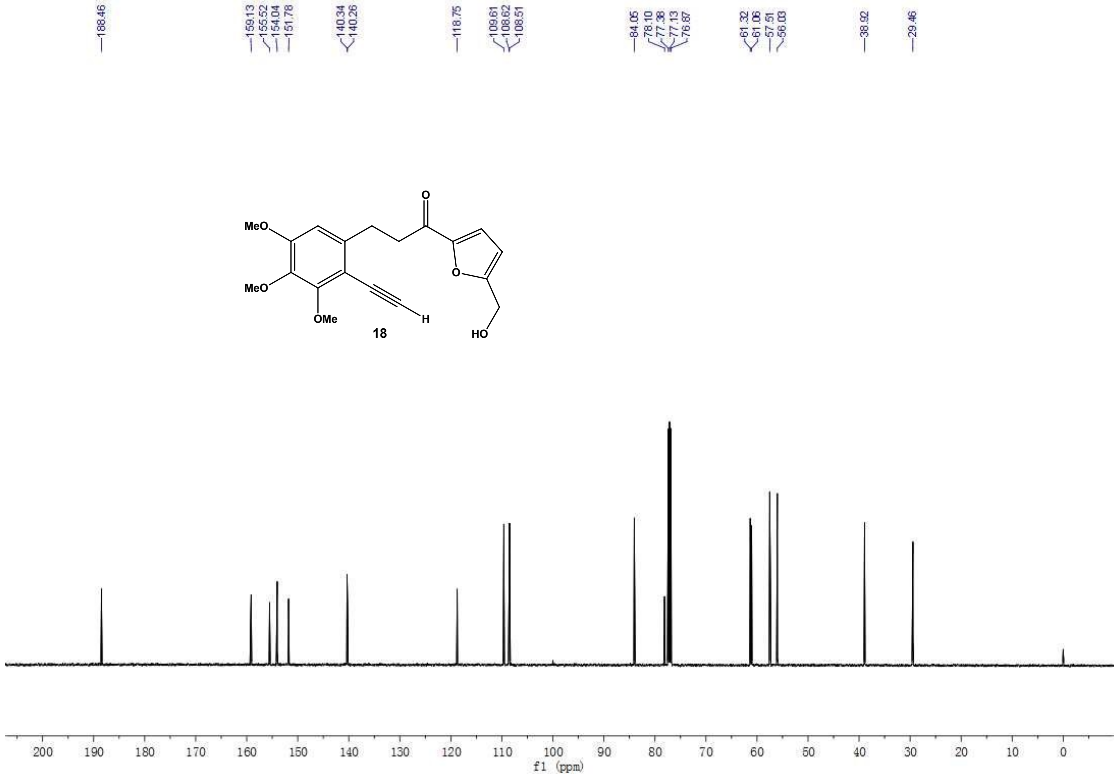
188.46
159.13
155.52
154.04
151.78
140.34
140.26
118.75
109.61
108.62
108.51
84.05
78.10
77.38
77.13
76.87
61.32
61.06
57.51
56.03
38.92
29.46
MeO
MeO
OMe
O
O
H
HO
18
f1 (ppm)

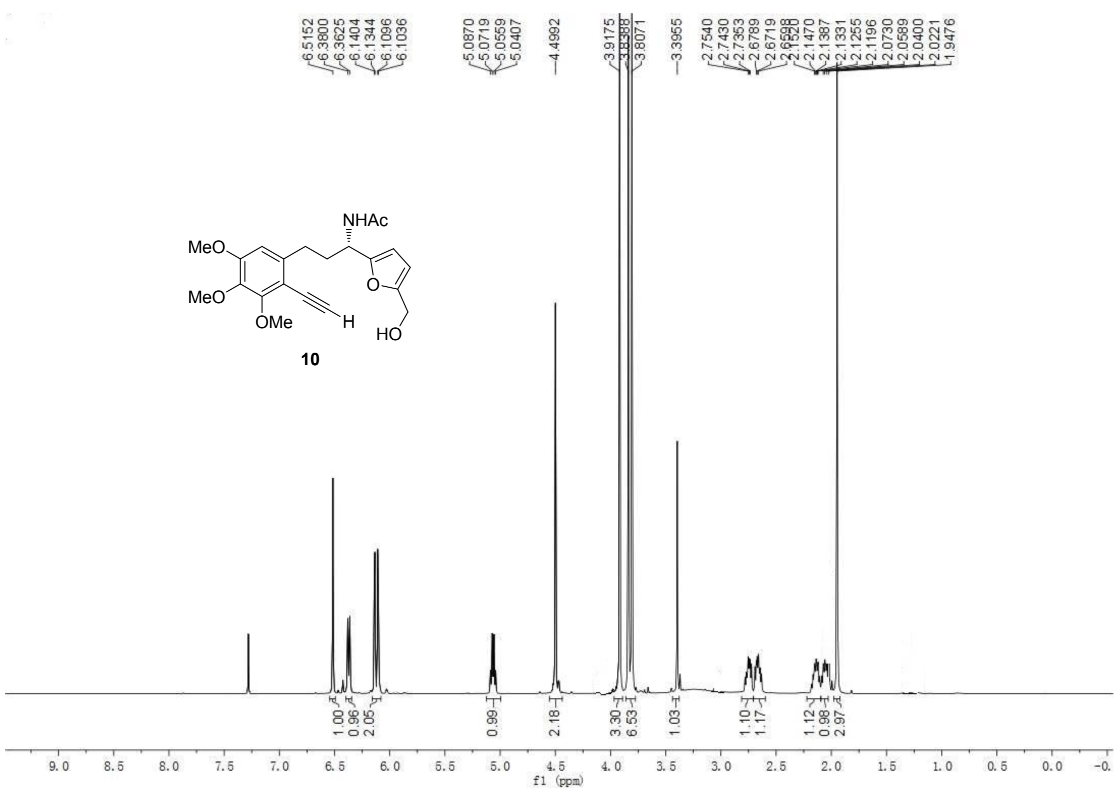

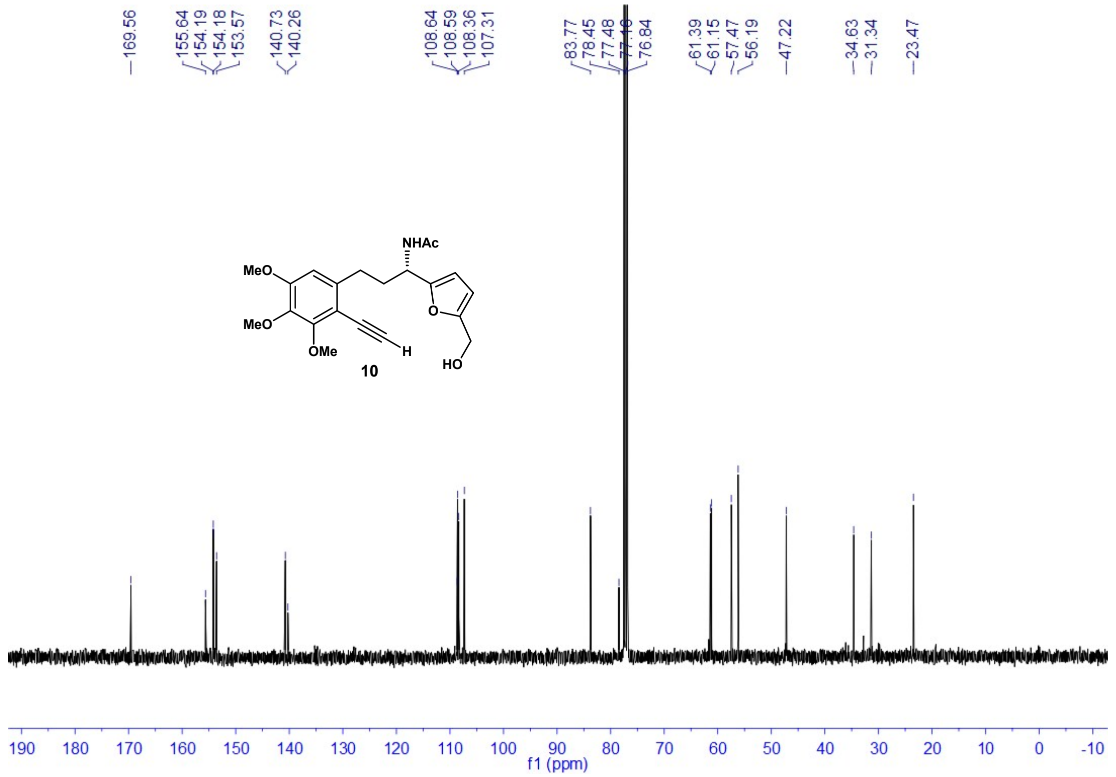

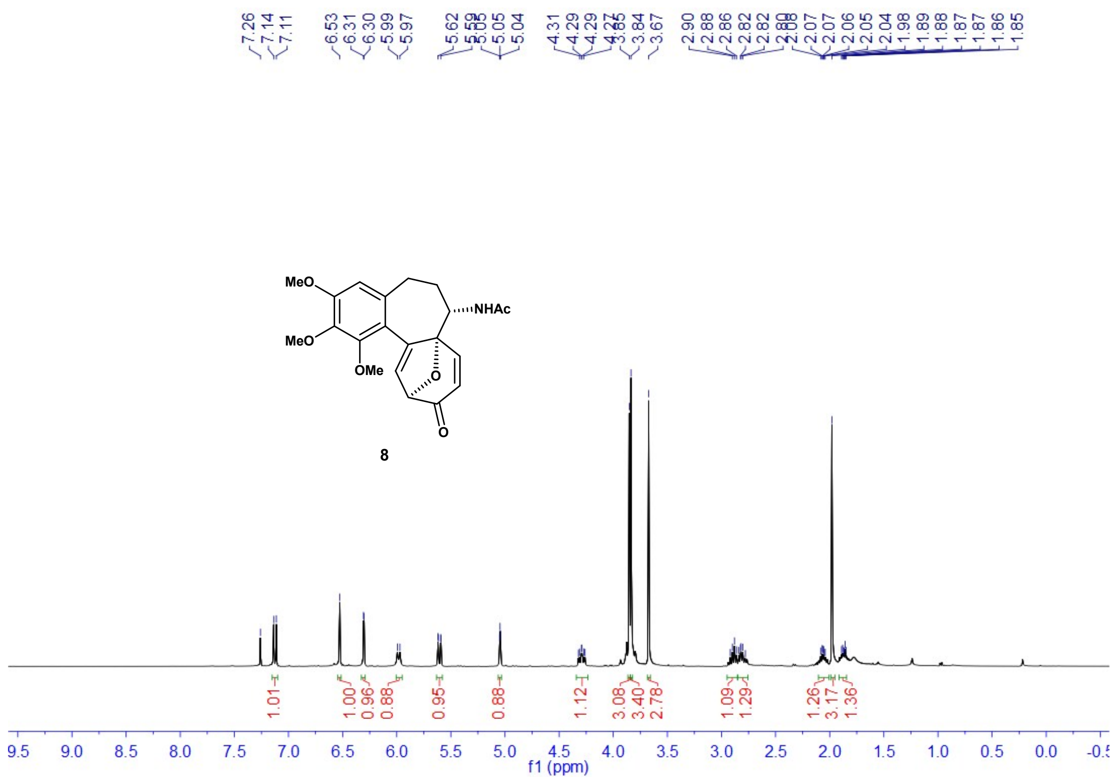

8

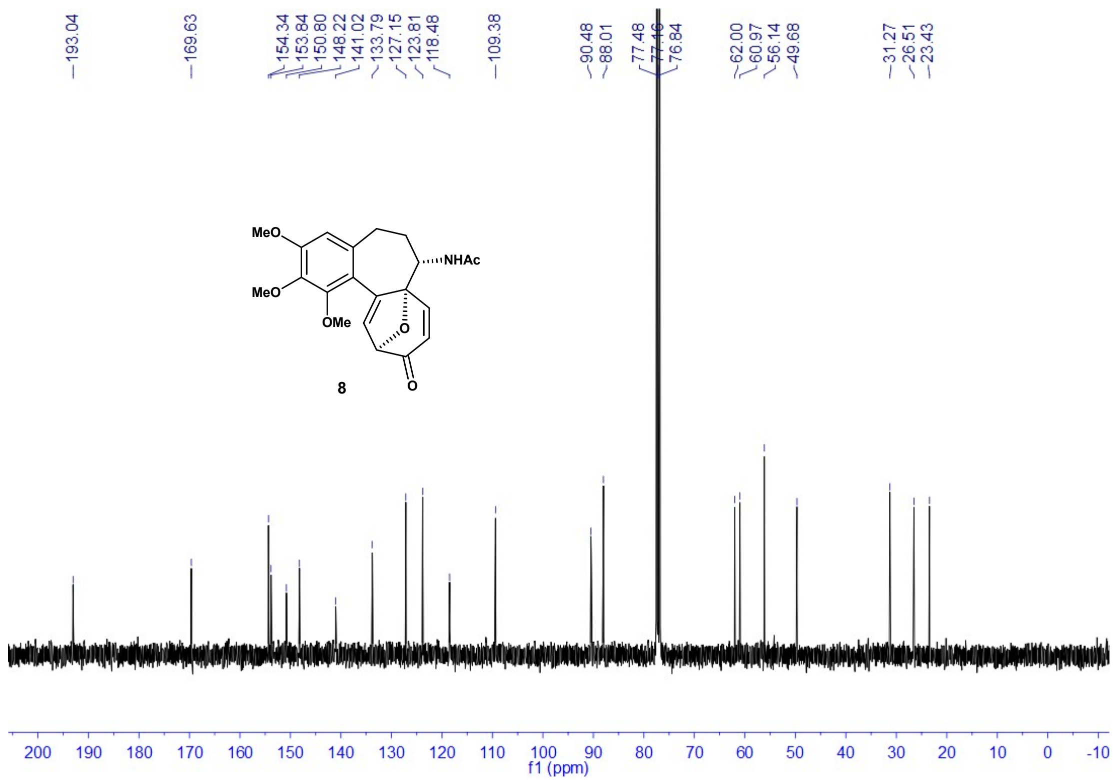

8

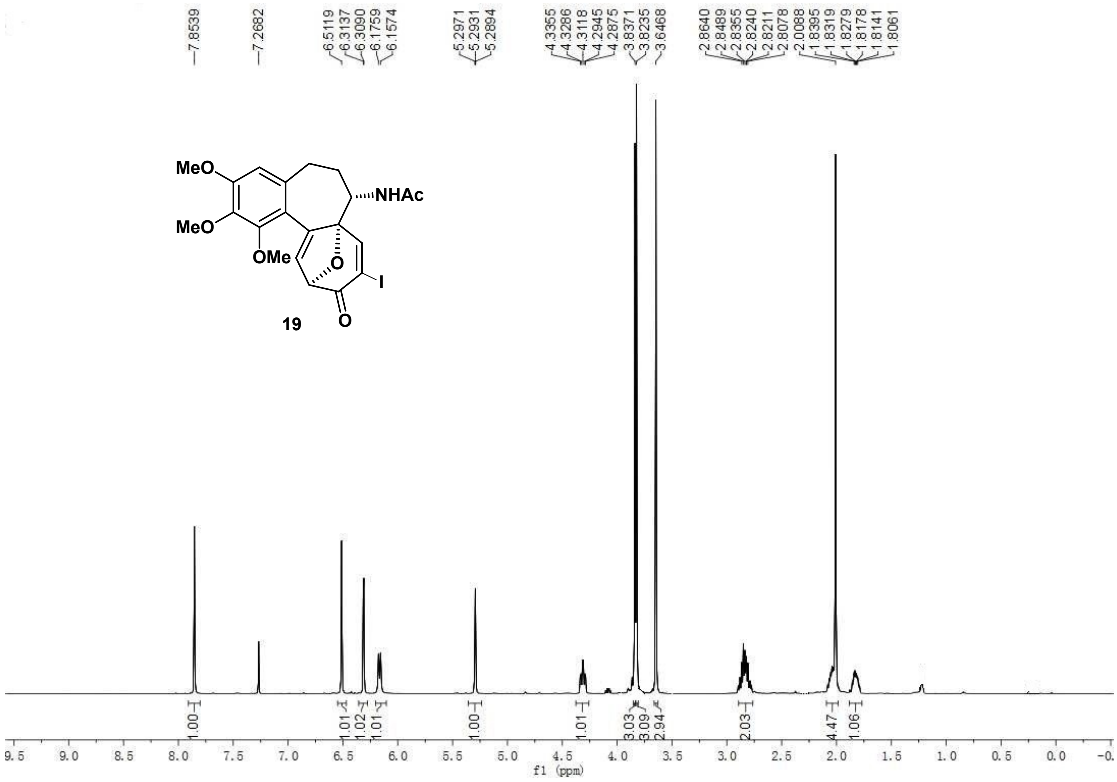
7.8539
7.2682
6.5119
6.3137
6.3090
6.1759
6.1574
5.2971
5.2931
5.2894
4.3355
4.3286
4.3118
4.2945
4.2875
3.8371
3.8235
3.6468
2.8640
2.8489
2.8355
2.8240
2.8211
2.8078
2.0088
1.8395
1.8319
1.8279
1.8178
1.8141
1.8061
MeO
MeO
OMe
O
NHAc
I
O
1.00
1.01
1.02
1.01
1.00
1.01
3.03
3.09
2.94
2.03
4.47
1.06
f1 (ppm)

19

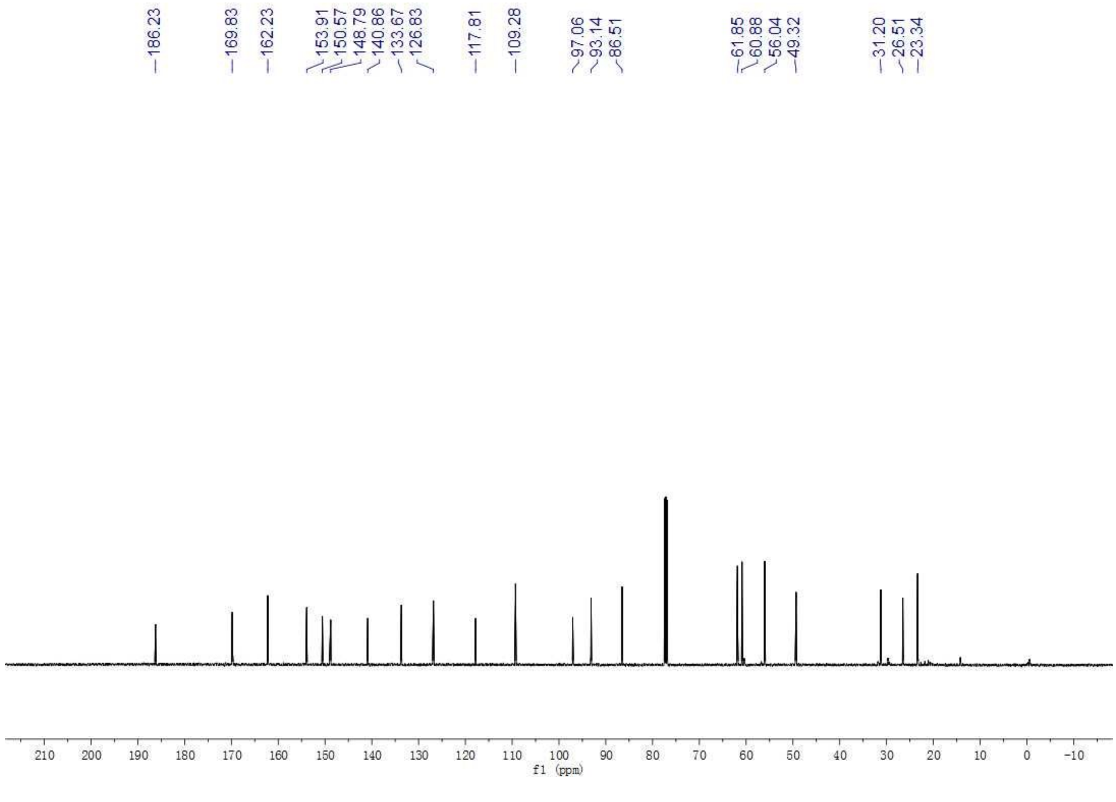
186.23
169.83
162.23
153.91
150.57
148.79
140.86
133.67
126.83
117.81
109.28
97.06
93.14
86.51
61.85
60.88
56.04
49.32
31.20
26.51
23.34
f1 (ppm)

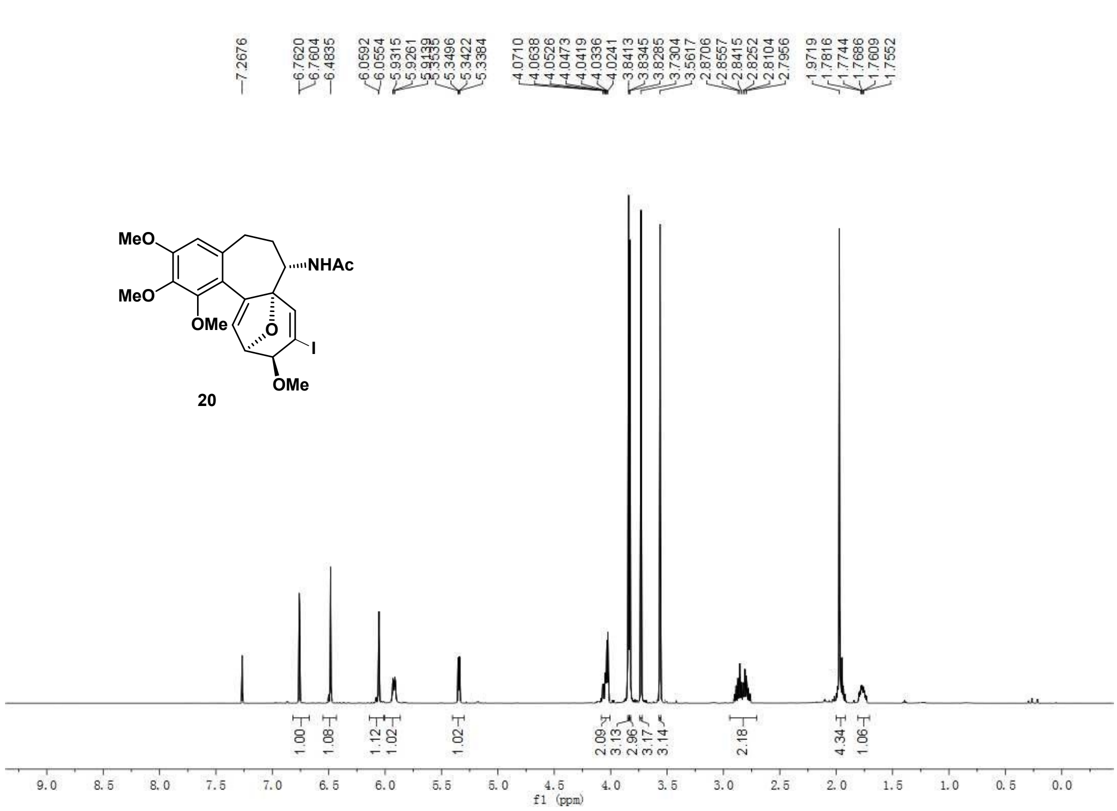

MeO
MeO
OMe
NHAc
O
I
OMe
20
f1 (ppm)

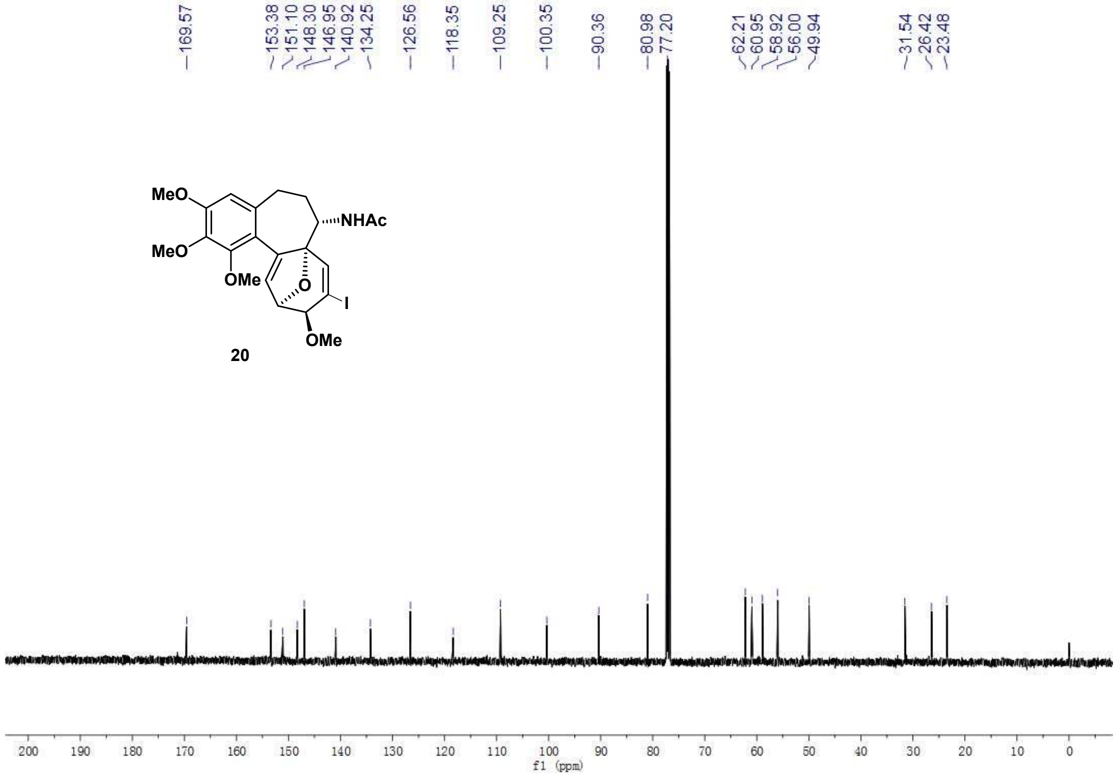

169.57
153.38
151.10
148.30
146.95
140.92
134.25
126.56
118.35
109.25
100.35
90.36
80.98
77.20
62.21
60.95
58.92
56.00
49.94
31.54
26.42
23.48
MeO
MeO
OMe
NHAc
O
I
OMe
20
f1 (ppm)

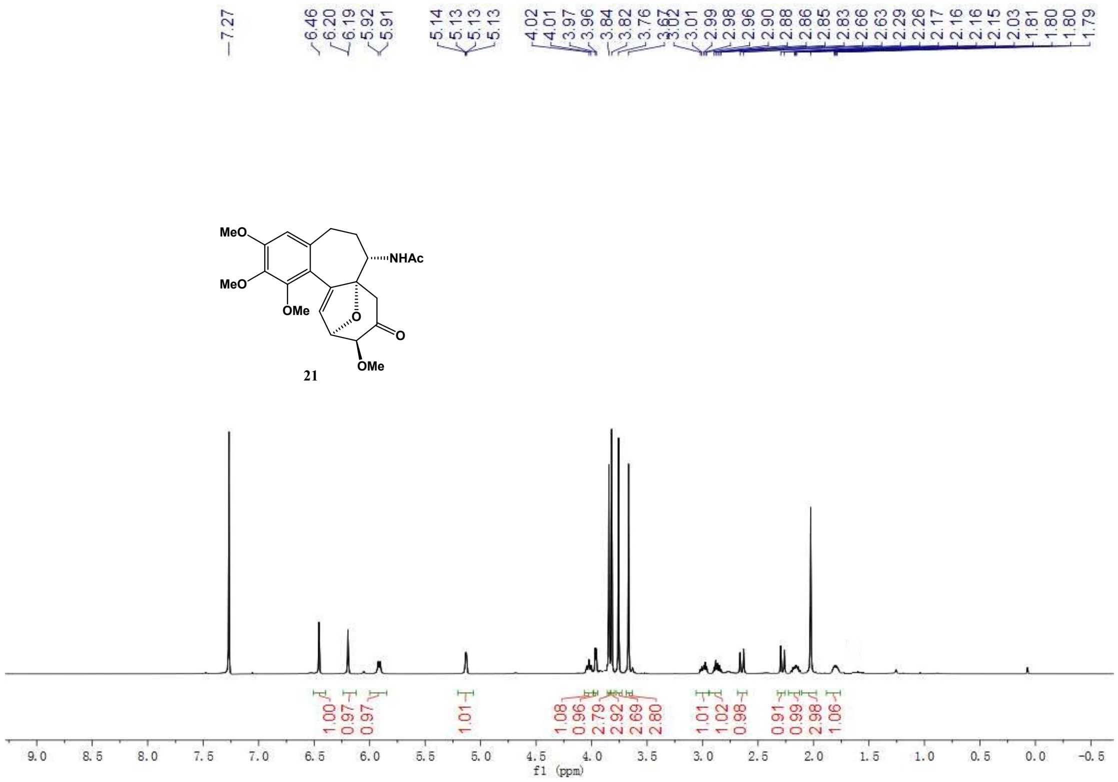
7.27
6.46
6.20
6.19
5.92
5.91
5.14
5.13
5.13
5.13
4.02
4.01
3.97
3.96
3.84
3.82
3.76
3.67
3.62
3.01
2.99
2.98
2.96
2.90
2.88
2.86
2.85
2.83
2.66
2.63
2.29
2.26
2.17
2.16
2.15
2.03
1.81
1.80
1.79
MeO
MeO
OMe
NHAc
O
O
OMe
21
1.00
0.97
0.97
1.01
1.08
0.96
2.79
2.92
2.69
2.80
1.01
1.02
0.98
0.91
0.99
2.98
1.06
f1 (ppm)

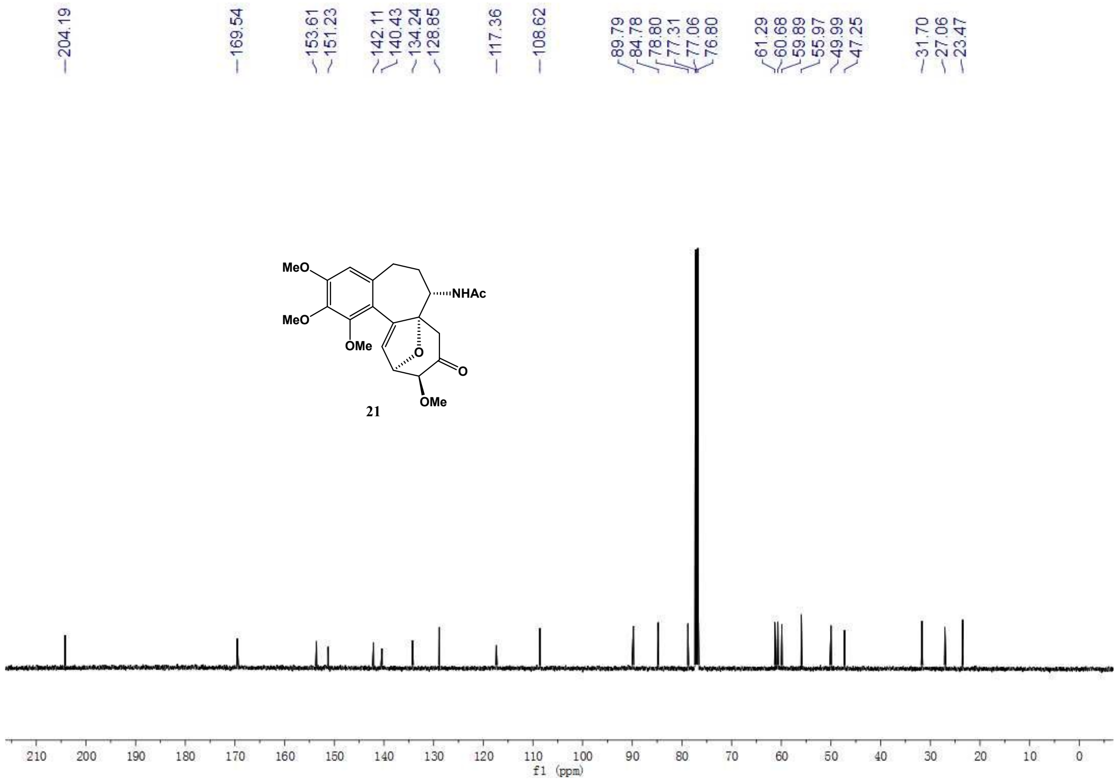
204.19
169.54
153.61
151.23
142.11
140.43
134.24
128.85
117.36
108.62
89.79
84.78
78.80
77.31
77.06
76.80
61.29
60.68
59.89
55.97
49.99
47.25
31.70
27.06
23.47
MeO
MeO
OMe
NHAc
O
O
OMe
21
f1 (ppm)

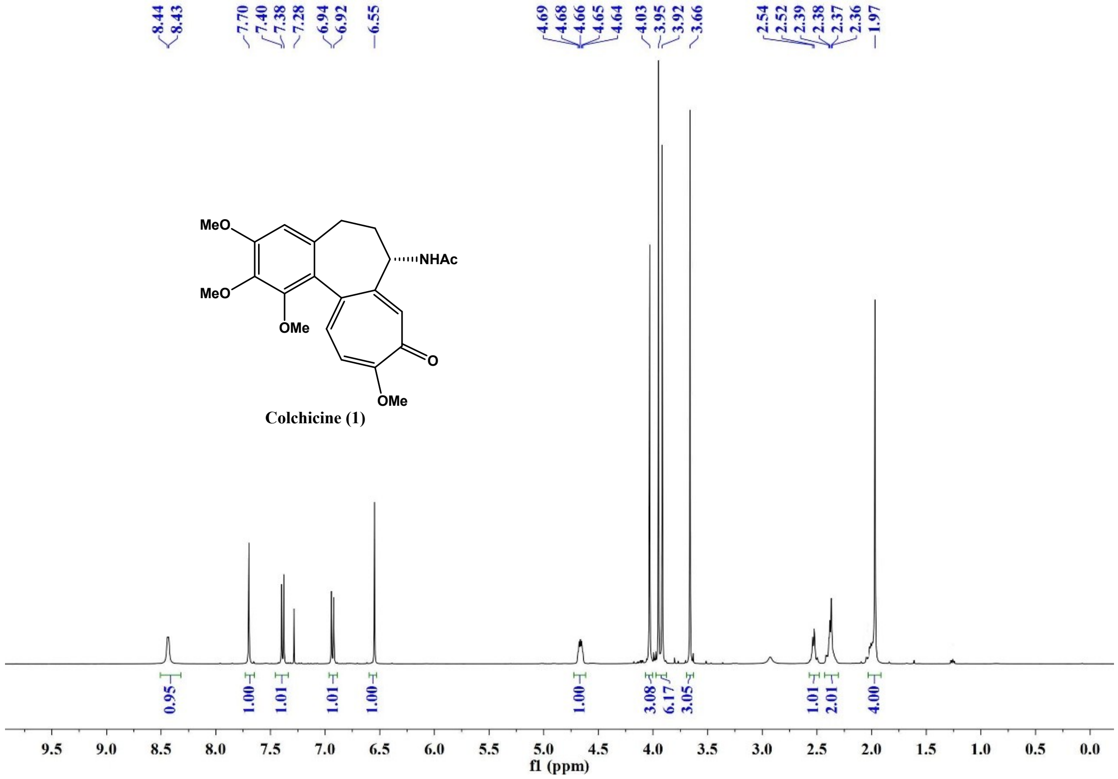

8.44
8.43
7.70
7.40
7.38
7.28
6.94
6.92
6.55
4.69
4.68
4.66
4.65
4.64
4.03
3.95
3.92
3.66
2.54
2.52
2.39
2.38
2.37
2.36
1.97
MeO
MeO
OMe
NHAc
O
OMe
Colchicine (1)
0.95
1.00
1.01
1.01
1.00
1.00
3.08
6.17
3.05
1.01
2.01
4.00
9.5 9.0 8.5 8.0 7.5 7.0 6.5 6.0 5.5 5.0 4.5 4.0 3.5 3.0 2.5 2.0 1.5 1.0 0.5 0.0
f1 (ppm)

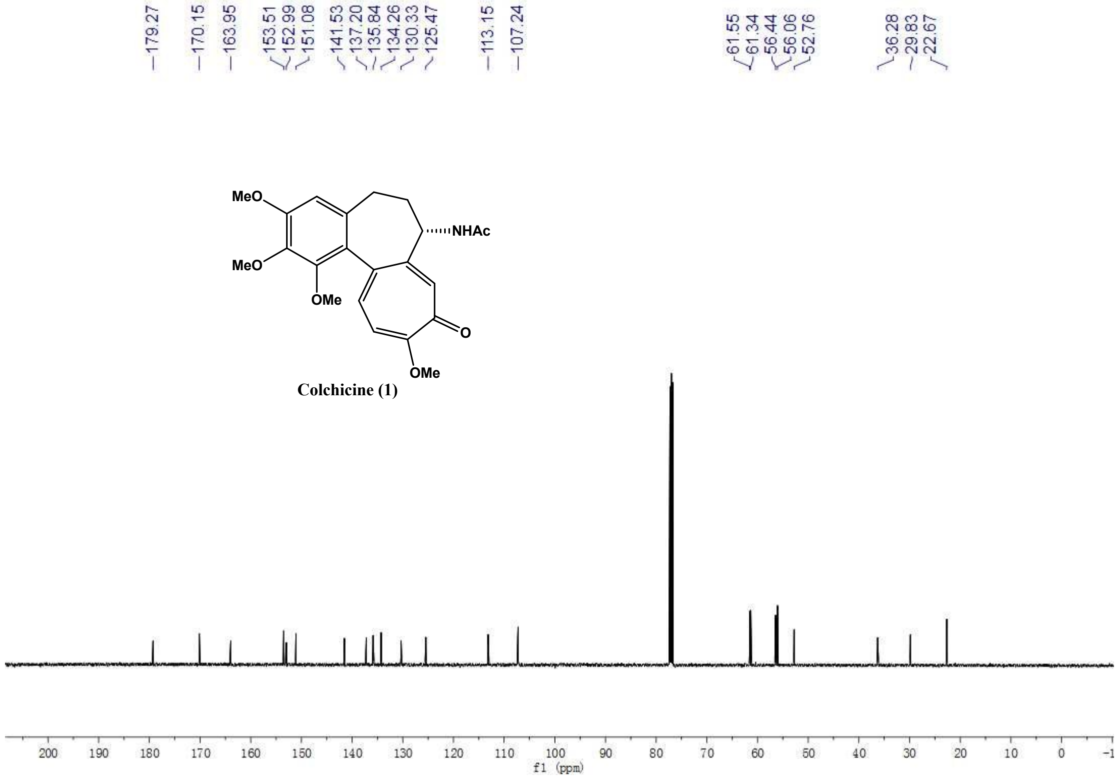

179.27
170.15
163.95
153.51
152.99
151.08
141.53
137.20
135.84
134.26
130.33
125.47
113.15
107.24
61.55
61.34
56.44
56.06
52.76
36.28
29.83
22.67
MeO
MeO
OMe
NHAc
O
OMe
Colchicine (1)
200 190 180 170 160 150 140 130 120 110 100 90 80 70 60 50 40 30 20 10 0 -1
f1 (ppm)

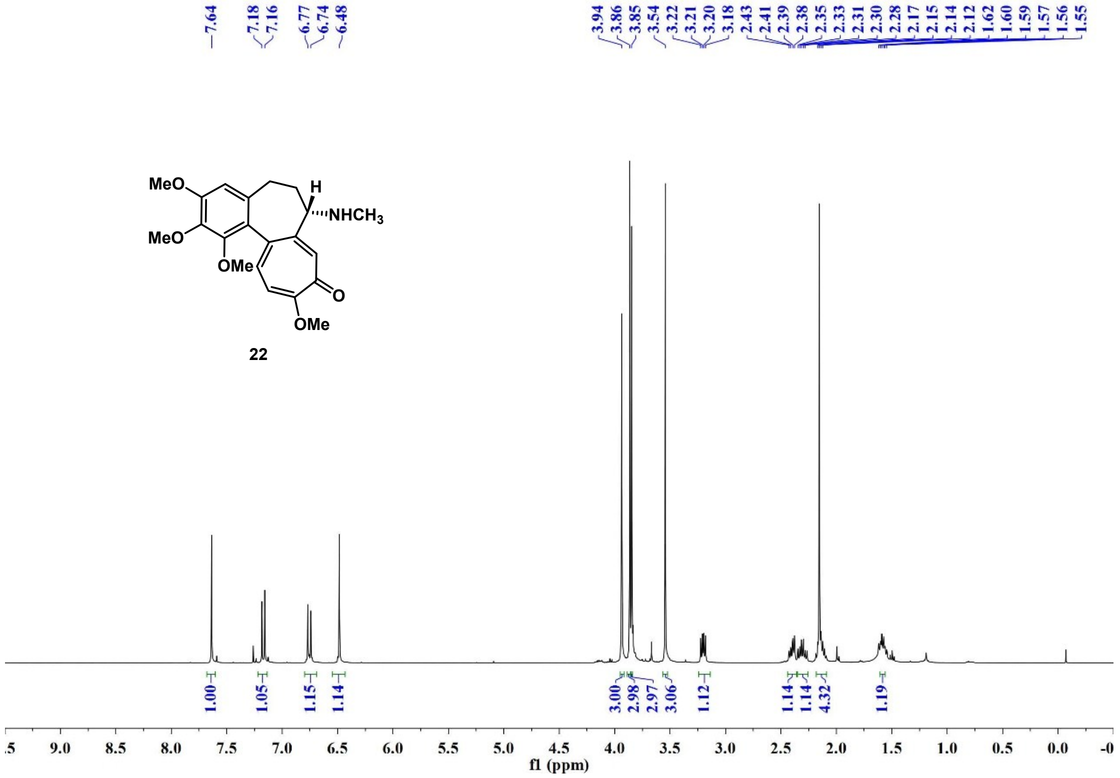
22
f1 (ppm)

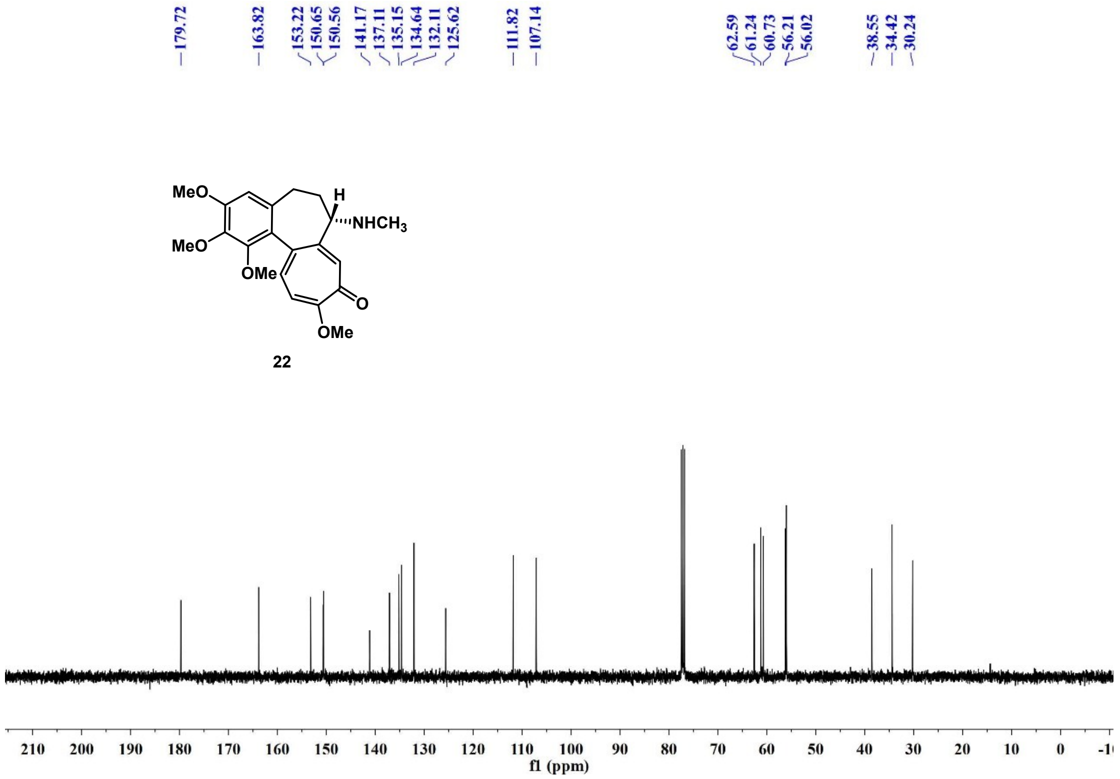
22
f1 (ppm)

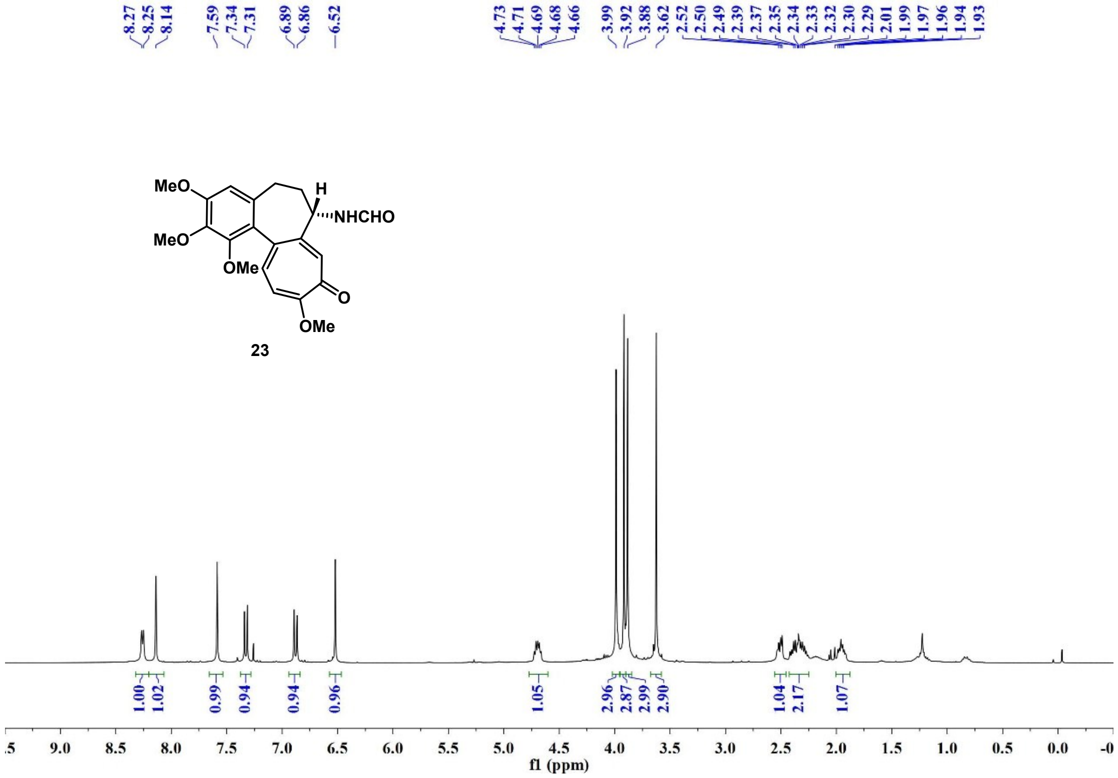
8.27
8.25
8.14
7.59
7.34
7.31
6.89
6.86
6.52
4.73
4.71
4.69
4.68
4.66
3.99
3.92
3.88
3.62
2.52
2.50
2.49
2.39
2.37
2.35
2.34
2.33
2.32
2.30
2.29
2.01
1.99
1.97
1.96
1.94
1.93
MeO
MeO
OMe
H
NHCHO
O
OMe
23
1.00
1.02
0.99
0.94
0.94
0.96
1.05
2.96
2.87
2.99
2.90
1.04
2.17
1.07
f1 (ppm)

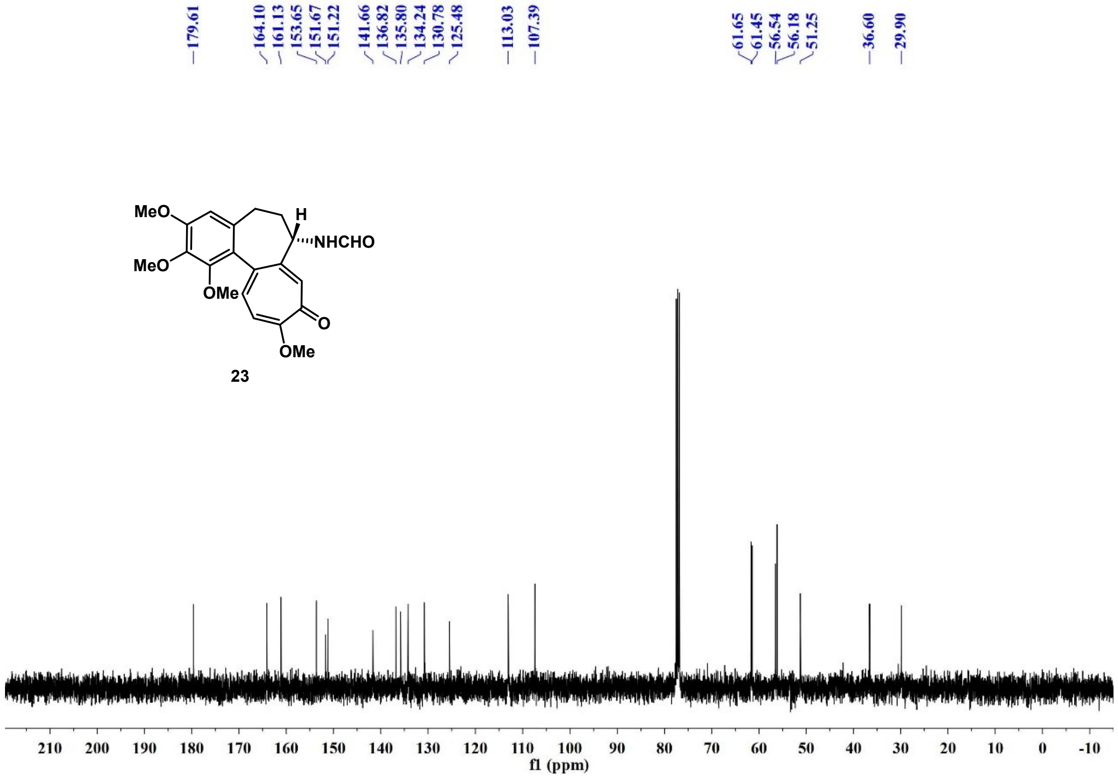
179.61
164.10
161.13
153.65
151.67
151.22
141.66
136.82
135.80
134.24
130.78
125.48
113.03
107.39
61.65
61.45
56.54
56.18
51.25
36.60
29.90
MeO
MeO
OMe
H
NHCHO
O
OMe
23
f1 (ppm)

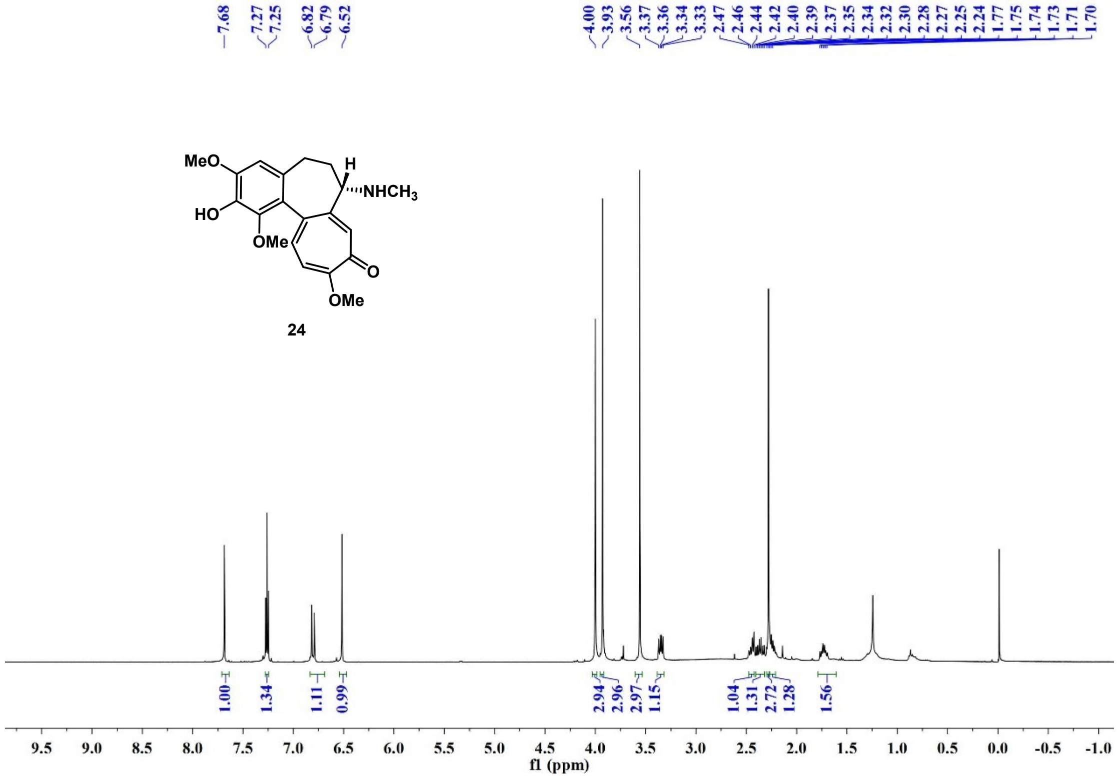
7.68
7.27
7.25
6.82
6.79
6.52
4.00
3.93
3.56
3.37
3.36
3.34
3.33
2.47
2.46
2.44
2.42
2.40
2.39
2.37
2.35
2.34
2.32
2.30
2.28
2.27
2.25
2.24
1.77
1.75
1.74
1.73
1.71
1.70
MeO
HO
OMe
H
NHCH3
O
OMe
24
1.00
1.34
1.11
0.99
2.94
2.96
2.97
1.15
1.04
1.31
2.72
1.28
1.56
f1 (ppm)

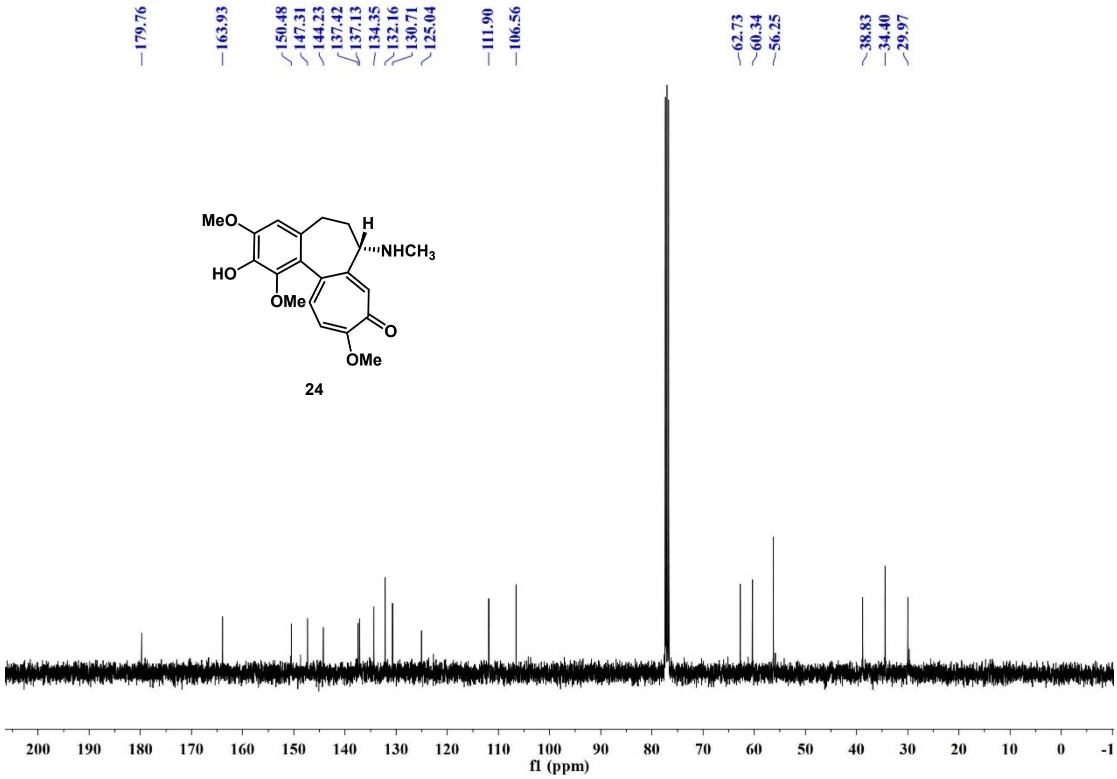
179.76
163.93
150.48
147.31
144.23
137.42
137.13
134.35
132.16
130.71
125.04
111.90
106.56
62.73
60.34
56.25
38.83
34.40
29.97
MeO
HO
OMe
H
NHCH3
O
OMe
24
f1 (ppm)

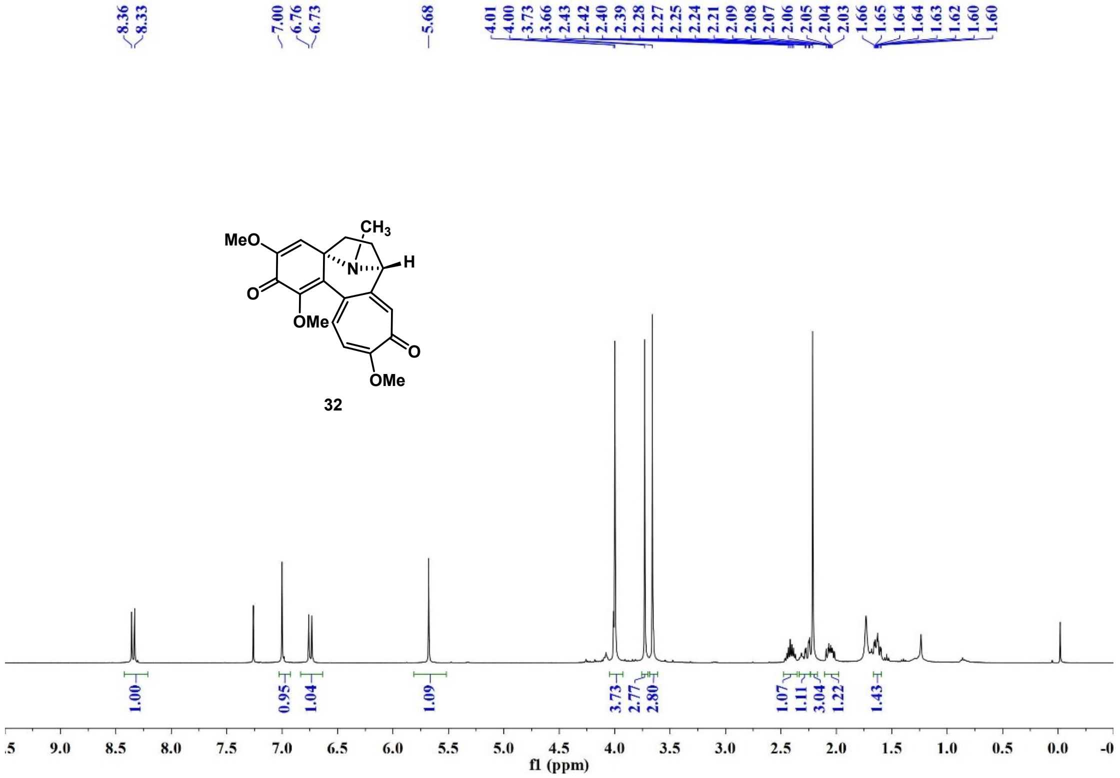
8.36
8.33
7.00
6.76
6.73
5.68
4.01
4.00
3.73
3.66
2.43
2.42
2.40
2.39
2.28
2.27
2.25
2.24
2.21
2.09
2.08
2.07
2.06
2.05
2.04
2.03
1.66
1.65
1.64
1.64
1.63
1.62
1.60
1.60
MeO
CH3
N
H
O
OMe
O
OMe
32
1.00
0.95
1.04
1.09
3.73
2.77
2.80
1.07
1.11
3.04
1.22
1.43
f1 (ppm)

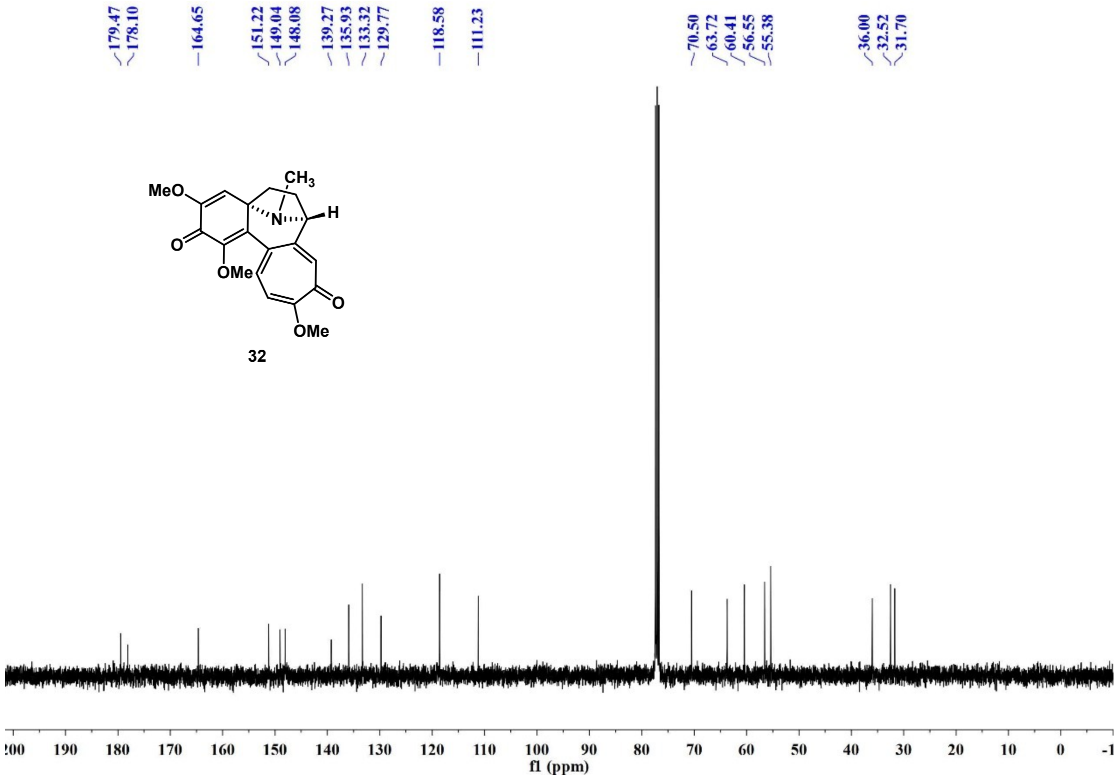
179.47
178.10
164.65
151.22
149.04
148.08
139.27
135.93
133.32
129.77
118.58
111.23
70.50
63.72
60.41
56.55
55.38
36.00
32.52
31.70
MeO
CH3
N
H
O
OMe
O
OMe
32
f1 (ppm)

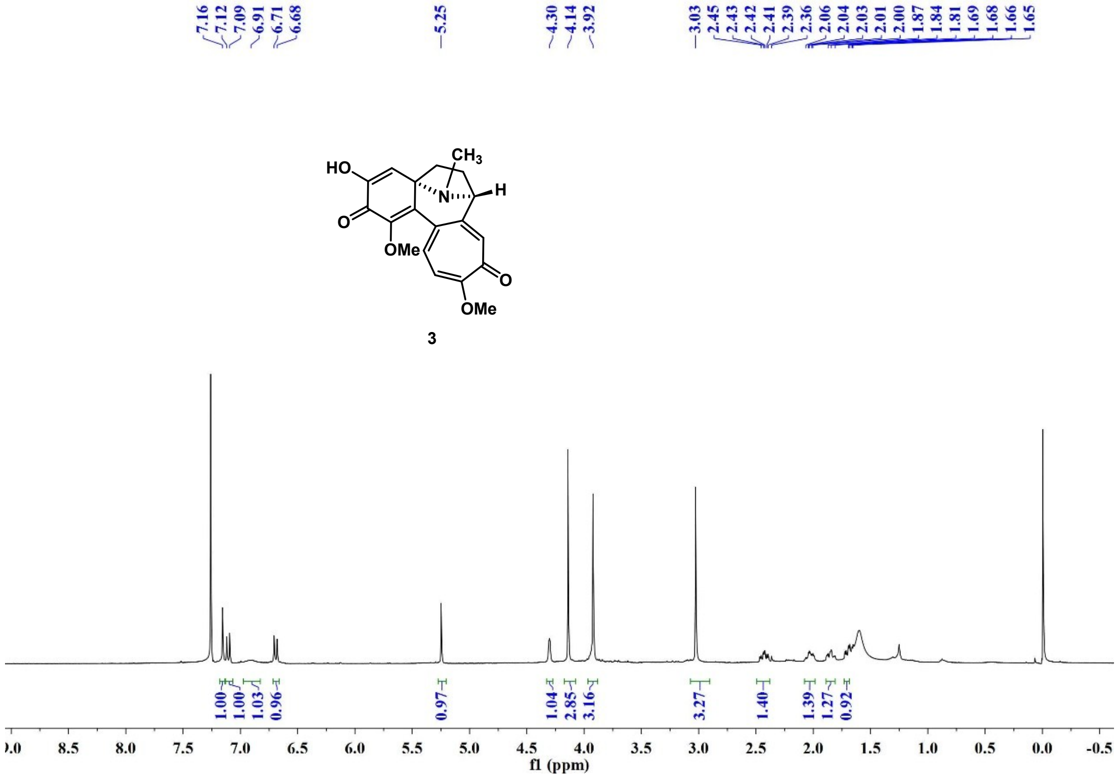
7.16
7.12
7.09
6.91
6.71
6.68
5.25
4.30
4.14
3.92
3.03
2.45
2.43
2.42
2.41
2.39
2.36
2.06
2.04
2.03
2.01
2.00
1.87
1.84
1.81
1.69
1.68
1.66
1.65
HO
CH3
N
H
O
OMe
O
OMe
3
1.00
1.00
1.03
0.96
0.97
1.04
2.85
3.16
3.27
1.40
1.39
1.27
0.92
f1 (ppm)

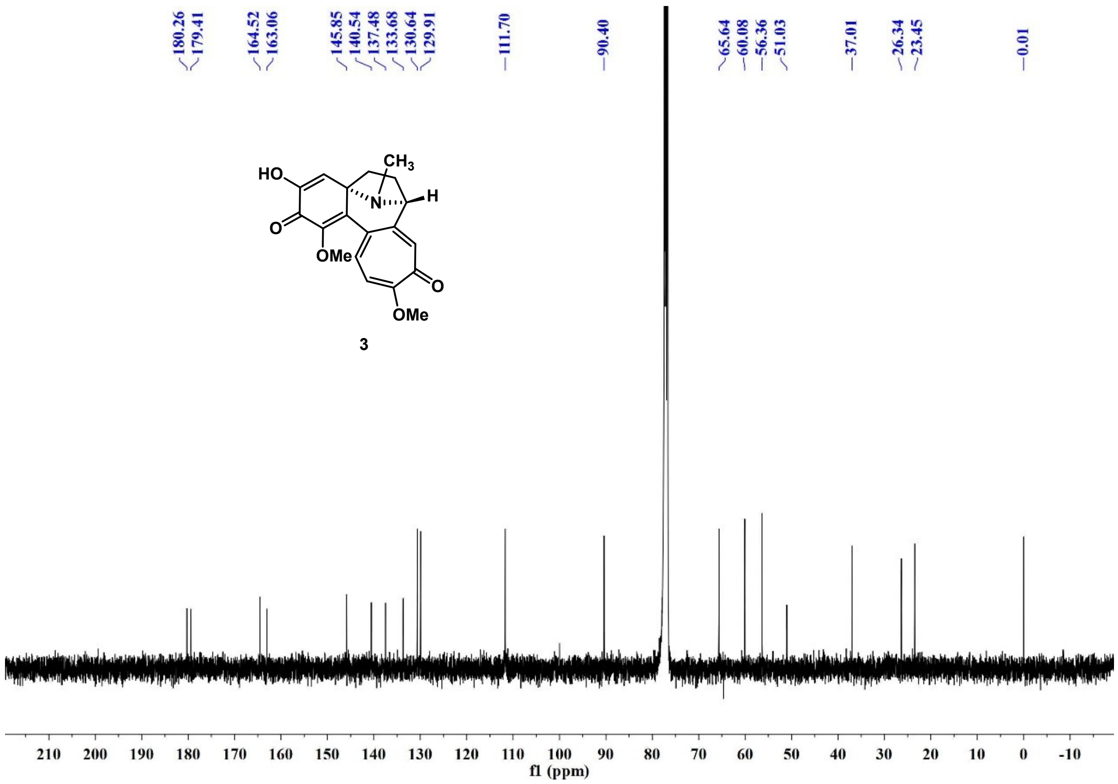
180.26
179.41
164.52
163.06
145.85
140.54
137.48
133.68
130.64
129.91
111.70
90.40
65.64
60.08
56.36
51.03
37.01
26.34
23.45
0.01
HO
CH3
N
H
O
OMe
O
OMe
3
f1 (ppm)

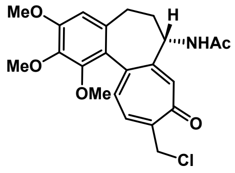

25

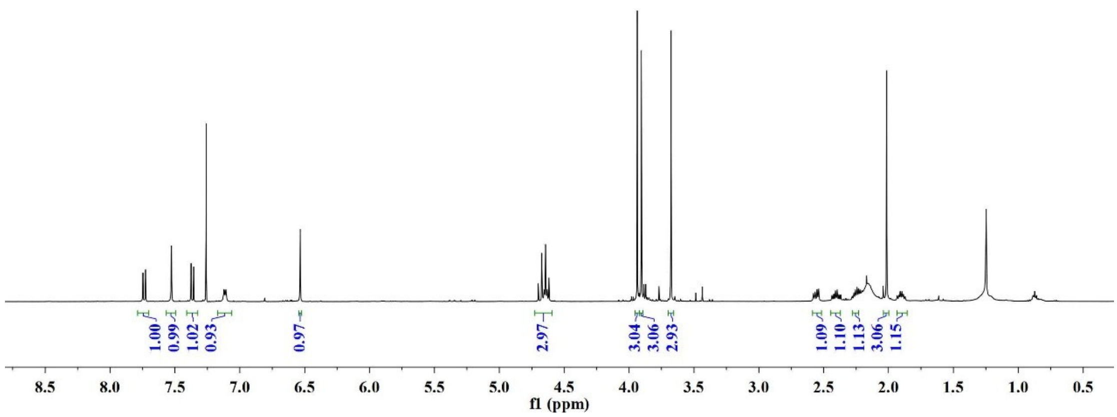

7.75 7.73 7.53 7.38 7.36 7.12 7.11 6.53 4.70 4.67 4.65 4.64 4.63 4.62 3.94 3.90 3.68 2.58 2.57 2.55 2.54 2.44 2.42 2.41 2.40 2.38 2.37 2.27 2.25 2.24 2.23 2.22 2.21 2.02 1.93 1.92 1.91 1.90 1.89
1.00 0.99 1.02 0.93 0.97 2.97 3.04 3.06 2.93 1.09 1.10 1.13 3.06 1.15
8.5 8.0 7.5 7.0 6.5 6.0 5.5 5.0 4.5 4.0 3.5 3.0 2.5 2.0 1.5 1.0 0.5
f1 (ppm)

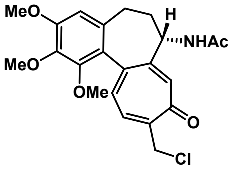

25

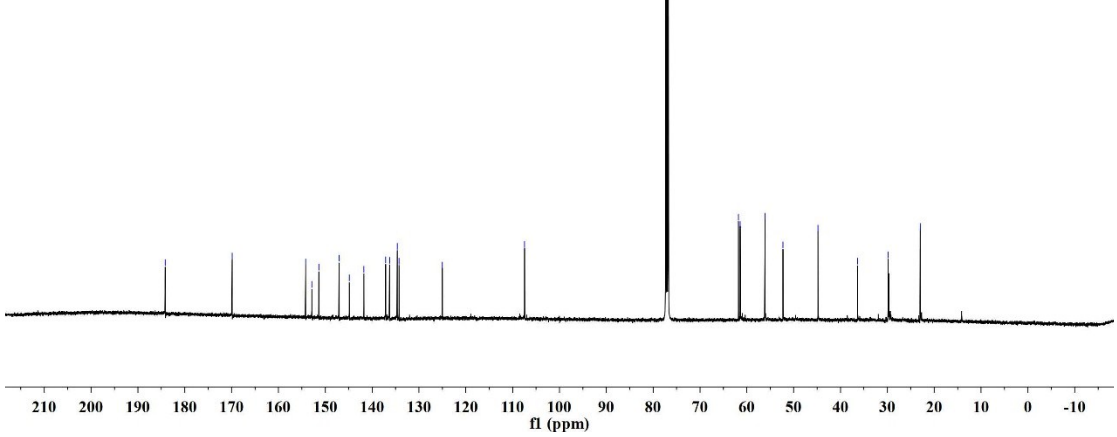

184.16 169.88 154.20 152.87 151.36 147.07 144.86 141.75 137.12 136.28 134.64 134.25 125.03 107.46 61.79 61.42 56.14 52.29 44.82 36.36 29.85 23.00
210 200 190 180 170 160 150 140 130 120 110 100 90 80 70 60 50 40 30 20 10 0 -10
f1 (ppm)

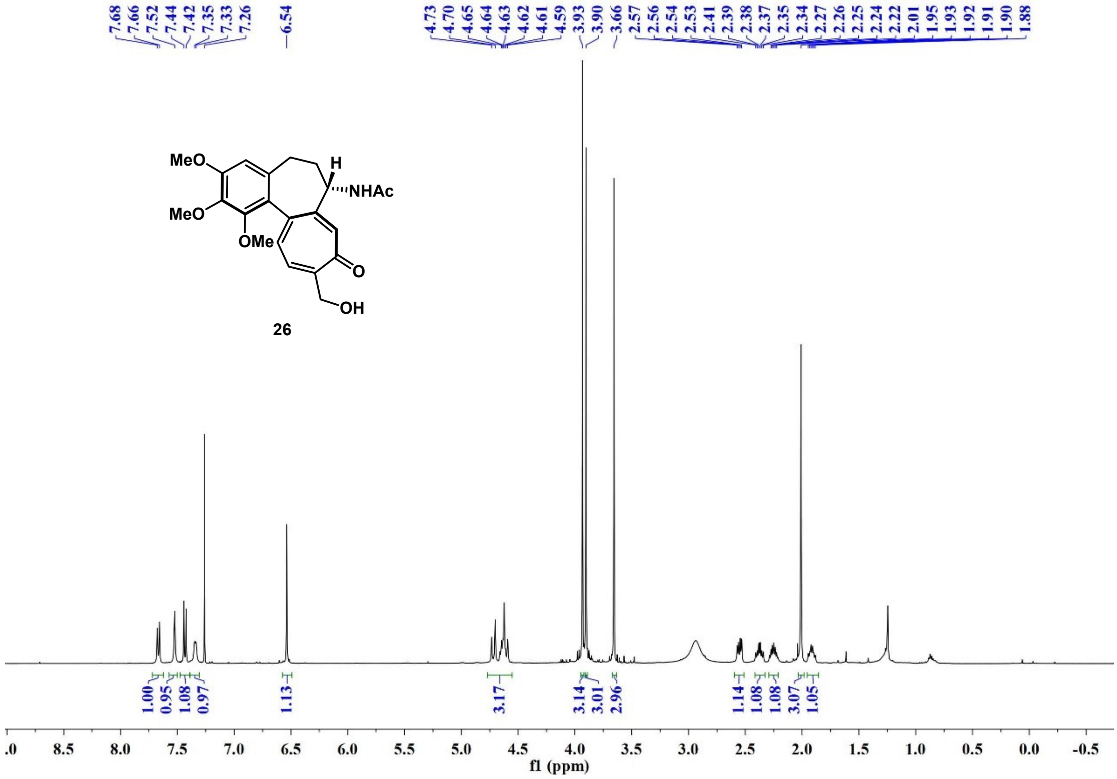
7.68 7.66 7.52 7.44 7.42 7.35 7.33 7.26 6.54
4.73 4.70 4.65 4.64 4.63 4.62 4.61 4.59 3.93 3.90 3.66 2.57 2.56 2.54 2.53 2.41 2.39 2.38 2.37 2.35 2.34 2.27 2.26 2.25 2.24 2.22 2.01 1.95 1.93 1.92 1.91 1.90 1.88
MeO
MeO
OMe
H
NHAc
O
OH
26
1.00 0.95 1.08 0.97 1.13 3.17 3.14 3.01 2.96 1.14 1.08 1.08 3.07 1.05
f1 (ppm)

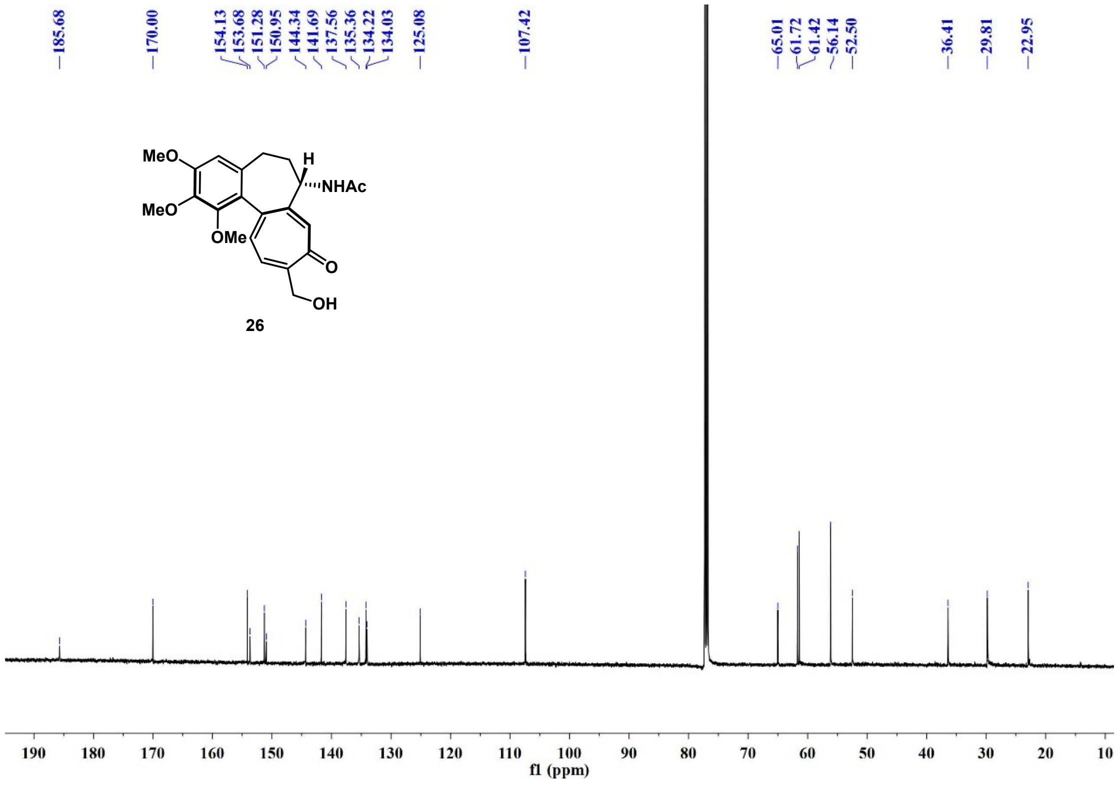
185.68 170.00 154.13 153.68 151.28 150.95 144.34 141.69 137.56 135.36 134.22 134.03 125.08 107.42
65.01 61.72 61.42 56.14 52.50 36.41 29.81 22.95
MeO
MeO
OMe
H
NHAc
O
OH
26
f1 (ppm)

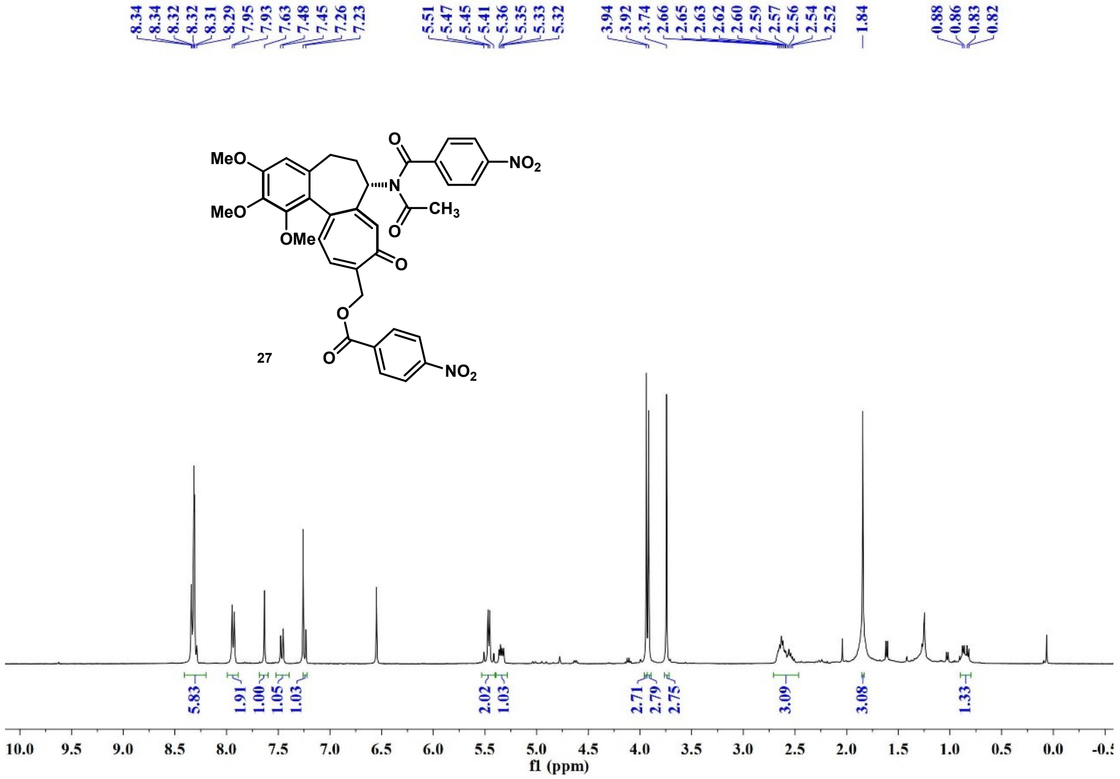

8.34
8.34
8.32
8.32
8.31
8.29
7.95
7.93
7.63
7.48
7.45
7.26
7.23
5.51
5.47
5.45
5.41
5.36
5.35
5.33
5.32
3.94
3.92
3.74
2.66
2.65
2.63
2.62
2.60
2.59
2.57
2.56
2.54
2.52
1.84
0.88
0.86
0.83
0.82
MeO
MeO
OMe
N
O
NO2
CH3
O
O
O
O
NO2
27
5.83
1.91
1.00
1.05
1.03
2.02
1.03
2.71
2.79
2.75
3.09
3.08
1.33
f1 (ppm)

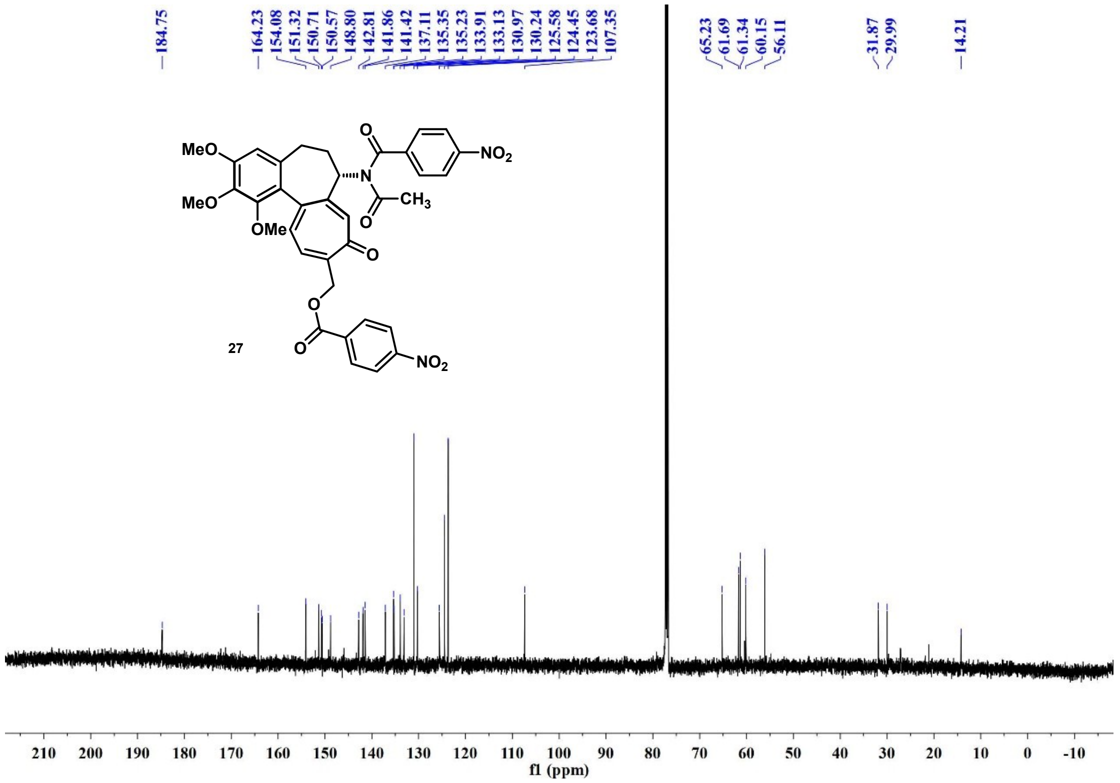

184.75
164.23
154.08
151.32
150.71
150.57
148.80
142.81
141.86
141.42
137.11
135.35
135.23
133.91
133.13
130.97
130.24
125.58
124.45
123.68
107.35
65.23
61.69
61.34
60.15
56.11
31.87
29.99
14.21
MeO
MeO
OMe
N
O
NO2
CH3
O
O
O
O
NO2
27
f1 (ppm)

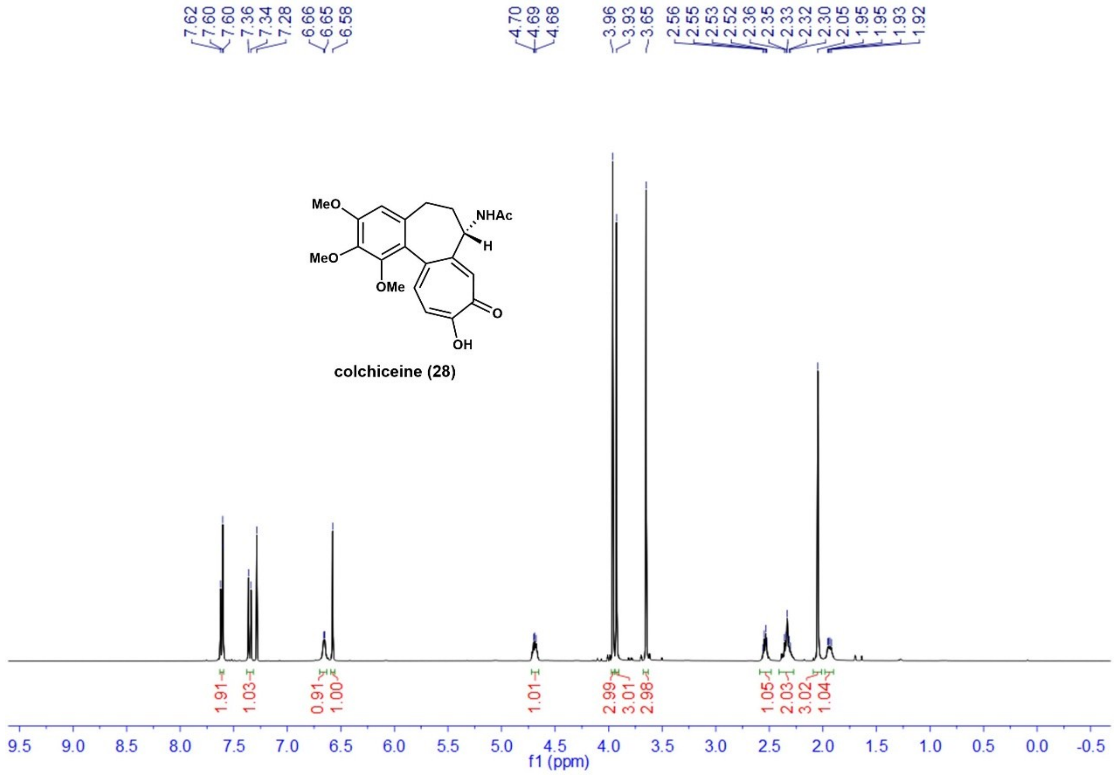
7.62 7.60 7.60 7.36 7.34 7.28 6.66 6.65 6.58
4.70 4.69 4.68
3.96 3.93 3.65
2.56 2.55 2.53 2.52 2.36 2.35 2.33 2.32 2.30 2.05 1.95 1.95 1.93 1.92
MeO
MeO
OMe
NHAc
H
O
OH
colchiceine (28)
1.91 1.03 0.91 1.00 1.01 2.99 3.01 2.98 1.05 2.03 3.02 1.04
9.5 9.0 8.5 8.0 7.5 7.0 6.5 6.0 5.5 5.0 4.5 4.0 3.5 3.0 2.5 2.0 1.5 1.0 0.5 0.0 -0.5
f1 (ppm)

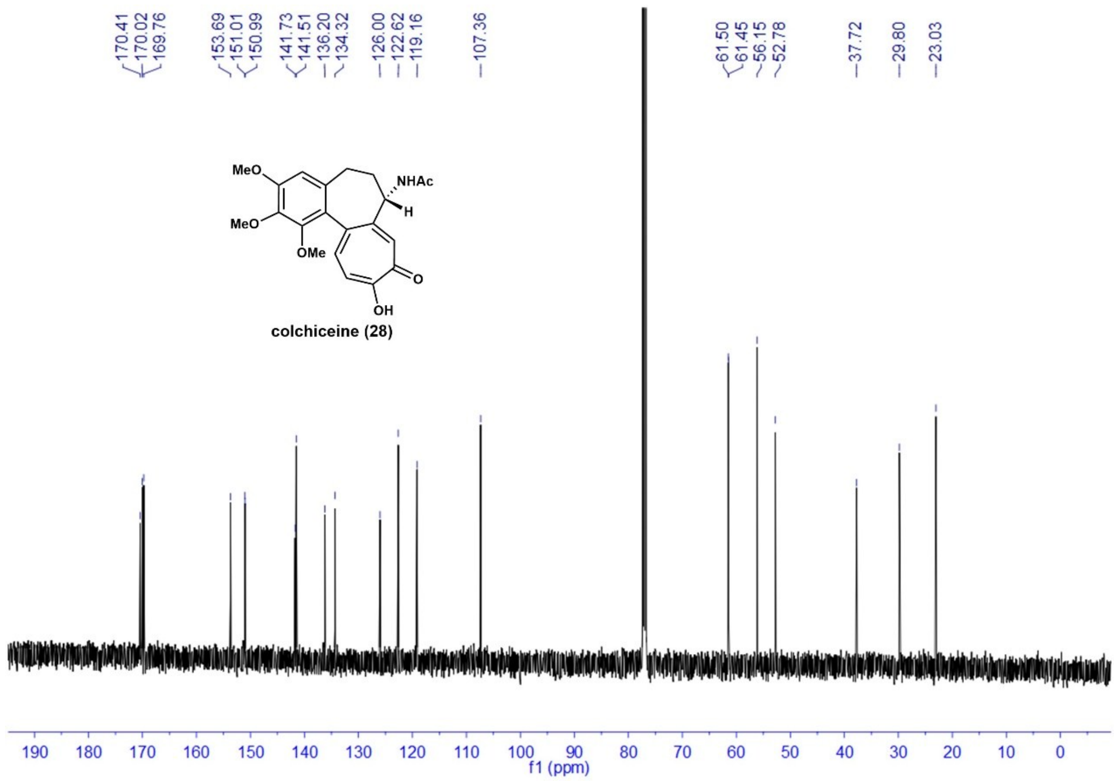
170.41 170.02 169.76
153.69 151.01 150.99
141.73 141.51 136.20 134.32
126.00 122.62 119.16
107.36
61.50 61.45 56.15 52.78
37.72
29.80
23.03
MeO
MeO
OMe
NHAc
H
O
OH
colchiceine (28)
190 180 170 160 150 140 130 120 110 100 90 80 70 60 50 40 30 20 10 0
f1 (ppm)

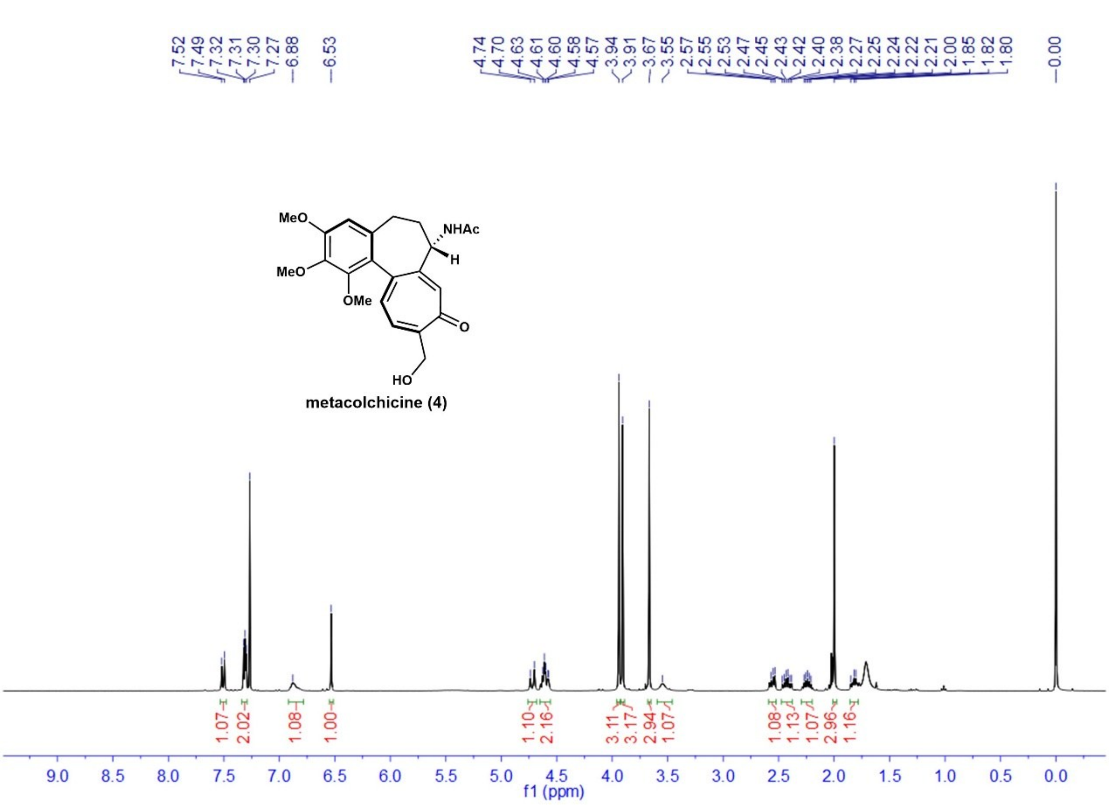
7.52
7.49
7.32
7.31
7.30
7.27
6.88
6.53
4.74
4.70
4.63
4.61
4.60
4.58
4.57
3.94
3.91
3.67
3.55
2.57
2.55
2.53
2.47
2.45
2.43
2.42
2.40
2.38
2.27
2.25
2.24
2.22
2.21
2.00
1.85
1.82
1.80
0.00
MeO
MeO
OMe
NHAc
H
O
HO
metacolchicine (4)
1.07
2.02
1.08
1.00
1.10
2.16
3.11
3.17
2.94
1.07
1.08
1.13
1.07
2.96
1.16
9.0 8.5 8.0 7.5 7.0 6.5 6.0 5.5 5.0 4.5 4.0 3.5 3.0 2.5 2.0 1.5 1.0 0.5 0.0
f1 (ppm)

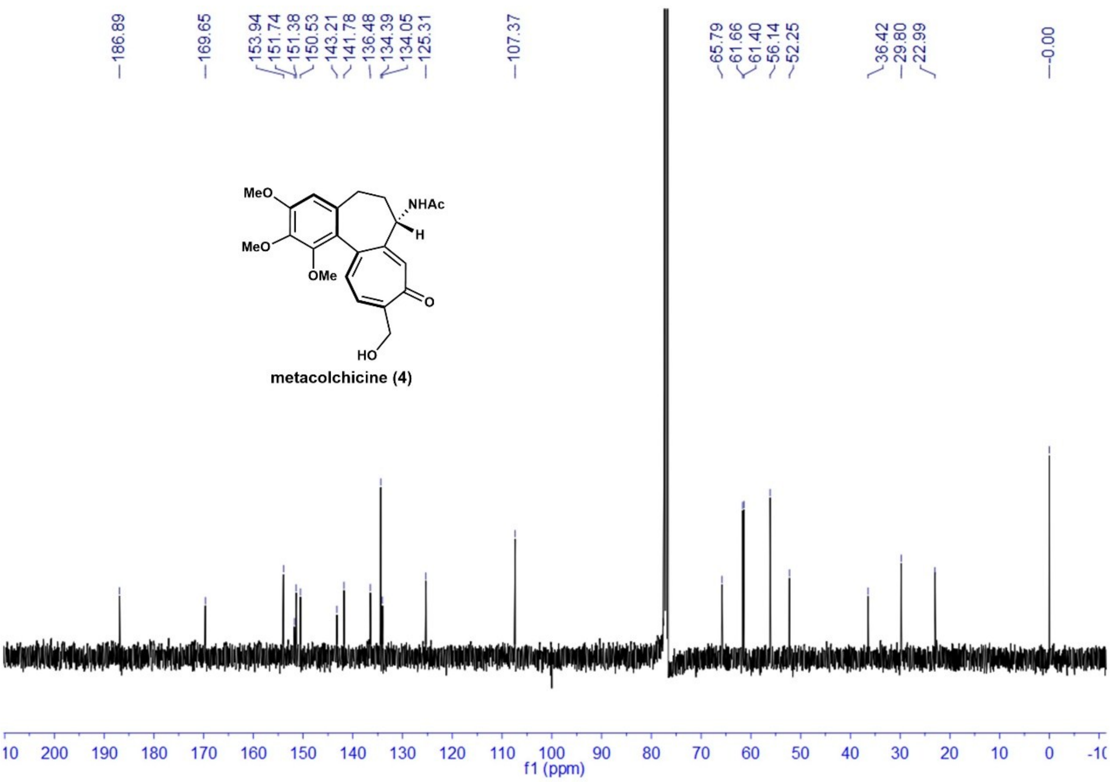
186.89
169.65
153.94
151.74
151.38
150.53
143.21
141.78
136.48
134.39
134.05
125.31
107.37
65.79
61.66
61.40
56.14
52.25
36.42
29.80
22.99
0.00
MeO
MeO
OMe
NHAc
H
O
HO
metacolchicine (4)
10 200 190 180 170 160 150 140 130 120 110 100 90 80 70 60 50 40 30 20 10 0 -1(
f1 (ppm)

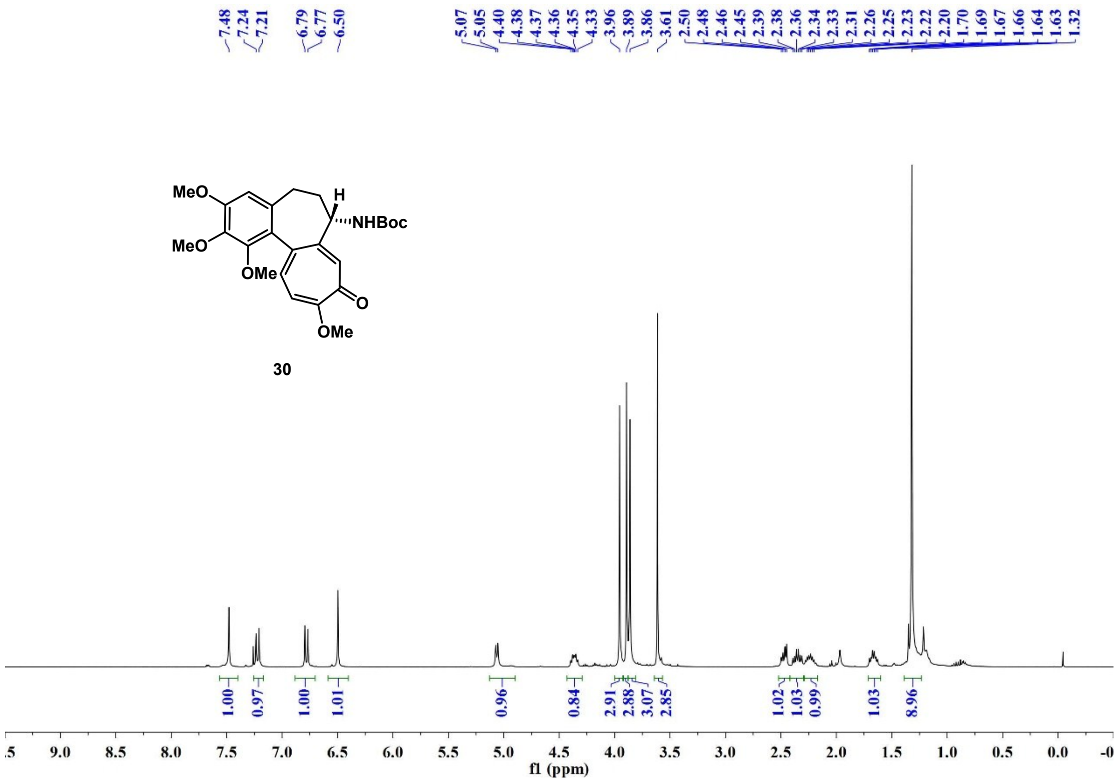
7.48
7.24
7.21
6.79
6.77
6.50
5.07
5.05
4.40
4.38
4.37
4.36
4.35
4.33
3.96
3.89
3.86
3.61
2.50
2.48
2.46
2.45
2.39
2.38
2.36
2.34
2.33
2.31
2.26
2.25
2.23
2.22
2.20
1.70
1.69
1.67
1.66
1.64
1.63
1.32
MeO
MeO
OMe
OMe
H
NHBoc
O
30
1.00
0.97
1.00
1.01
0.96
0.84
2.91
2.88
3.07
2.85
1.02
1.03
0.99
1.03
8.96
f1 (ppm)

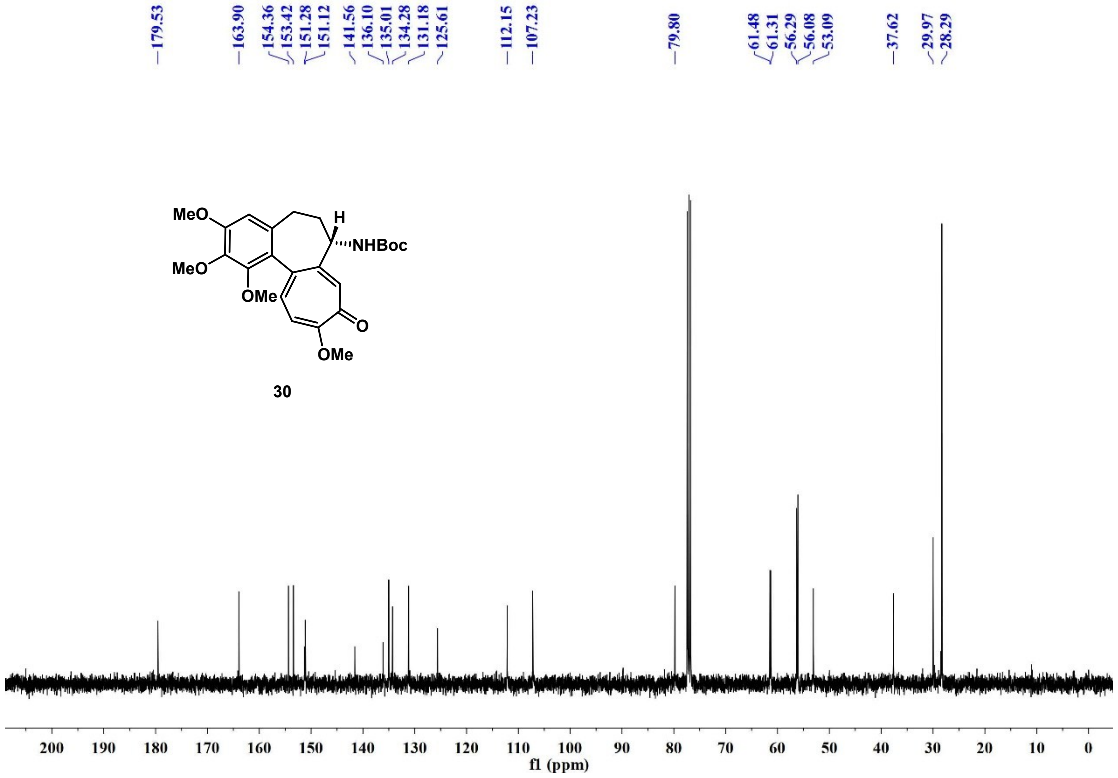
179.53
163.90
154.36
153.42
151.28
151.12
141.56
136.10
135.01
134.28
131.18
125.61
112.15
107.23
79.80
61.48
61.31
56.29
56.08
53.09
37.62
29.97
28.29
MeO
MeO
OMe
OMe
H
NHBoc
O
30
f1 (ppm)

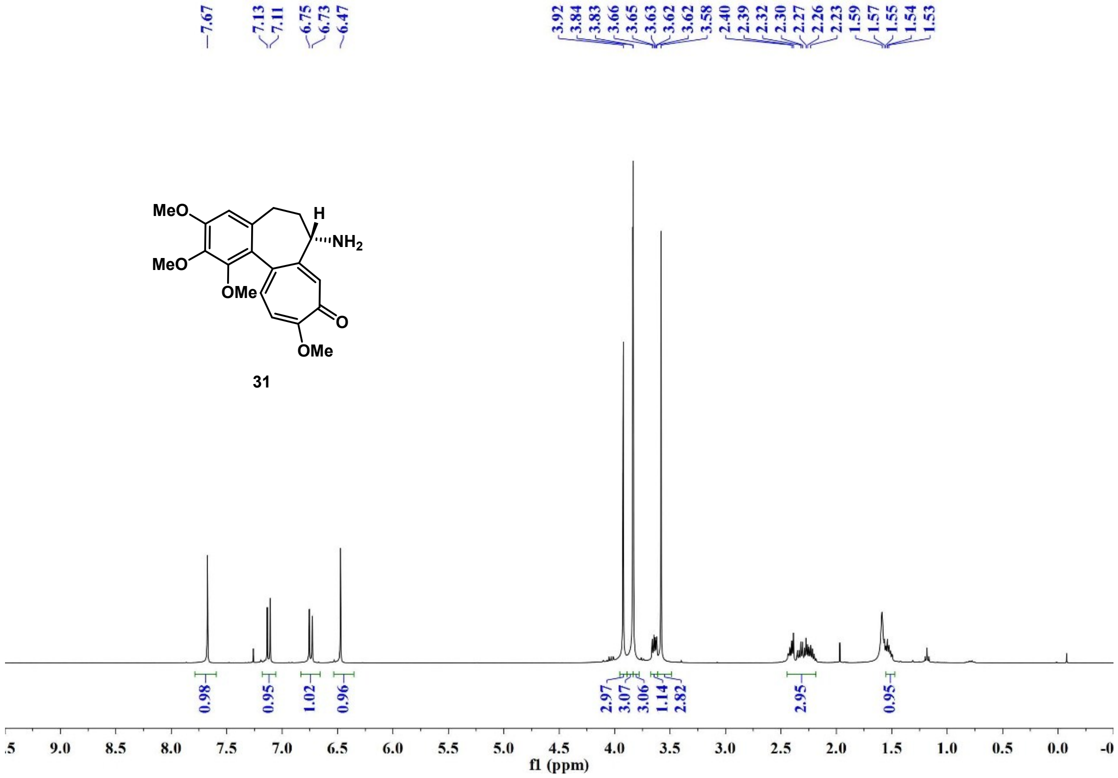
7.67
7.13
7.11
6.75
6.73
6.47
3.92
3.84
3.83
3.66
3.65
3.63
3.62
3.62
3.58
2.40
2.39
2.32
2.30
2.27
2.26
2.23
1.59
1.57
1.55
1.54
1.53
MeO
MeO
OMe
OMe
H
NH2
O
0.98
0.95
1.02
0.96
2.97
3.07
3.06
1.14
2.82
2.95
0.95
f1 (ppm)

31

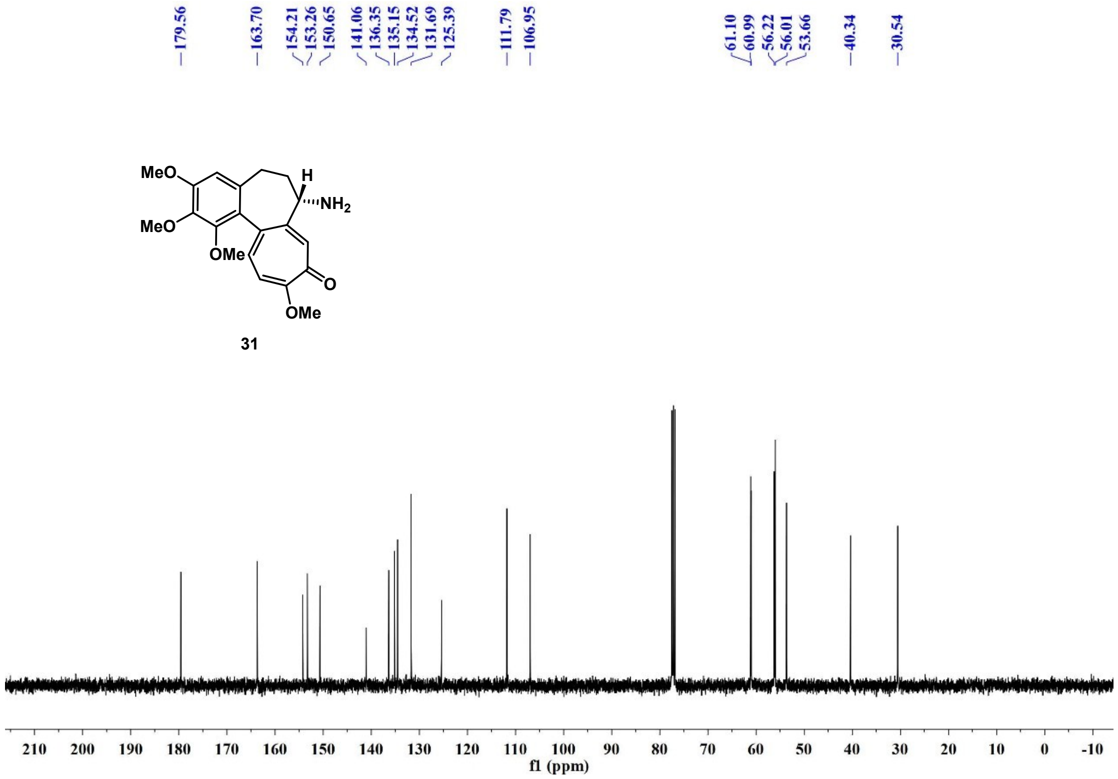
179.56
163.70
154.21
153.26
150.65
141.06
136.35
135.15
134.52
131.69
125.39
111.79
106.95
61.10
60.99
56.22
56.01
53.66
40.34
30.54
MeO
MeO
OMe
OMe
H
NH2
O
f1 (ppm)

31

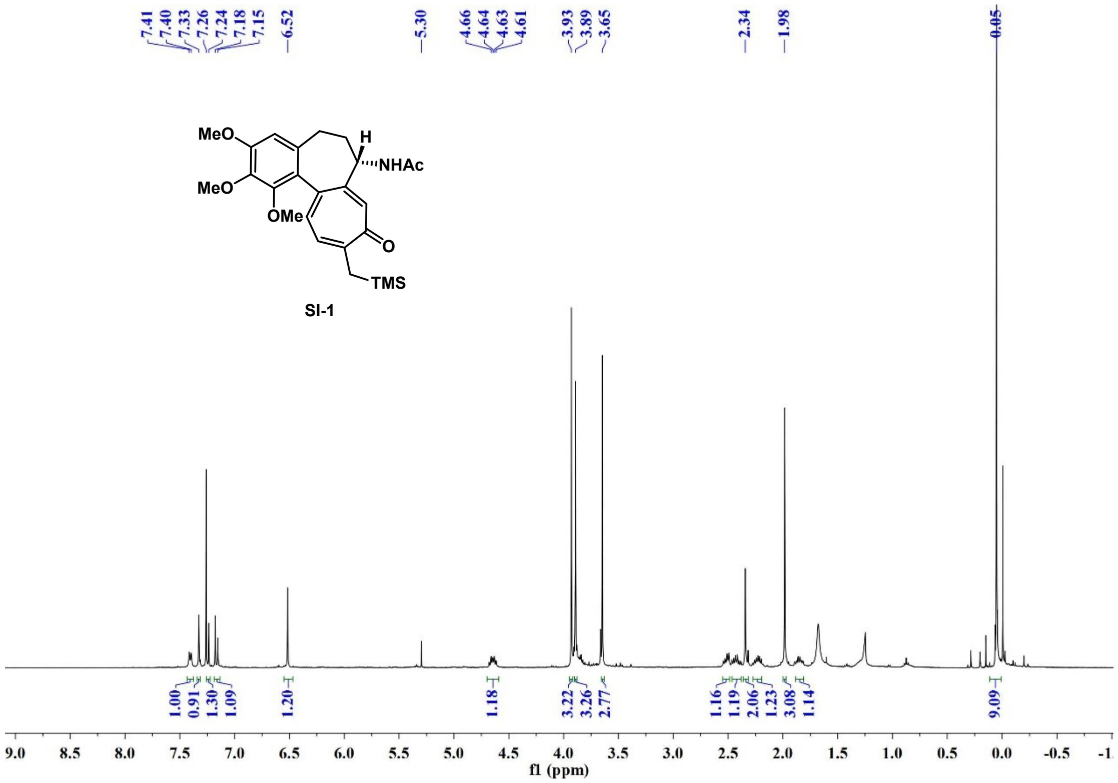
7.41
7.40
7.33
7.26
7.24
7.18
7.15
6.52
5.30
4.66
4.64
4.63
4.61
3.93
3.89
3.65
2.34
1.98
0.05
MeO
MeO
OMe
H
NHAc
O
TMS
SI-1
1.00
0.91
1.30
1.09
1.20
1.18
3.22
3.26
2.77
1.16
1.19
2.06
1.23
3.08
1.14
9.09
f1 (ppm)

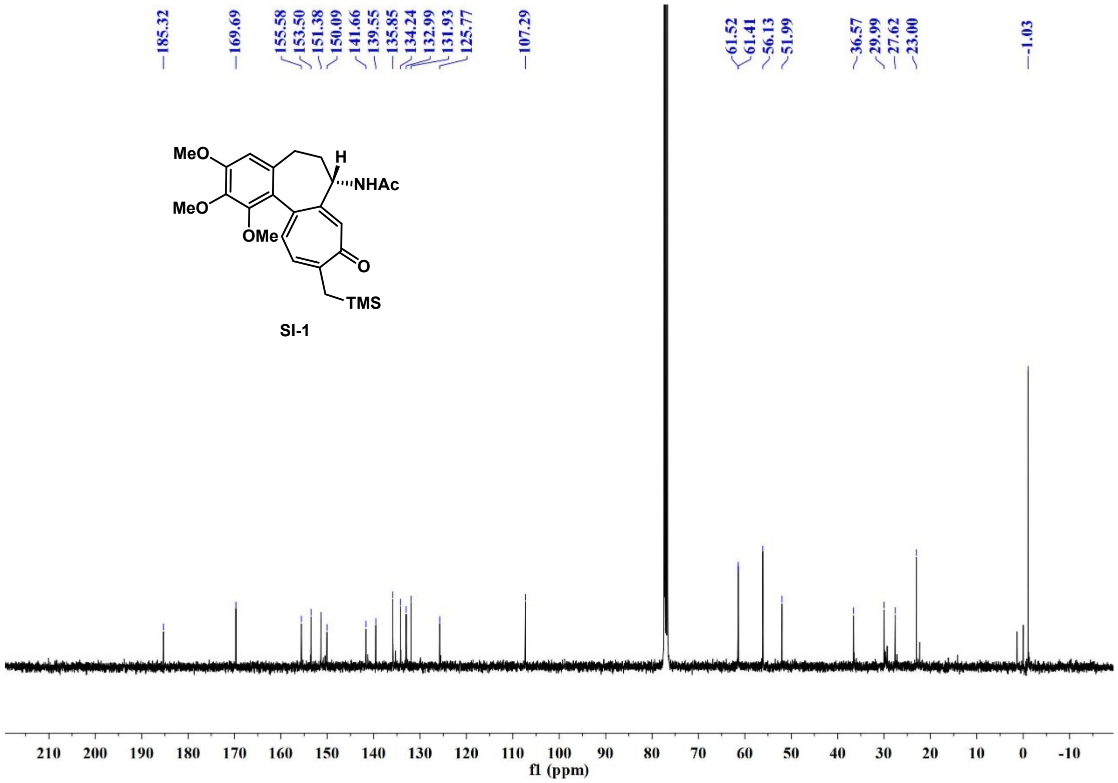
185.32
169.69
155.58
153.50
151.38
150.09
141.66
139.55
135.85
134.24
132.99
131.93
125.77
107.29
61.52
61.41
56.13
51.99
36.57
29.99
27.62
23.00
-1.03
MeO
MeO
OMe
H
NHAc
O
TMS
SI-1
f1 (ppm)

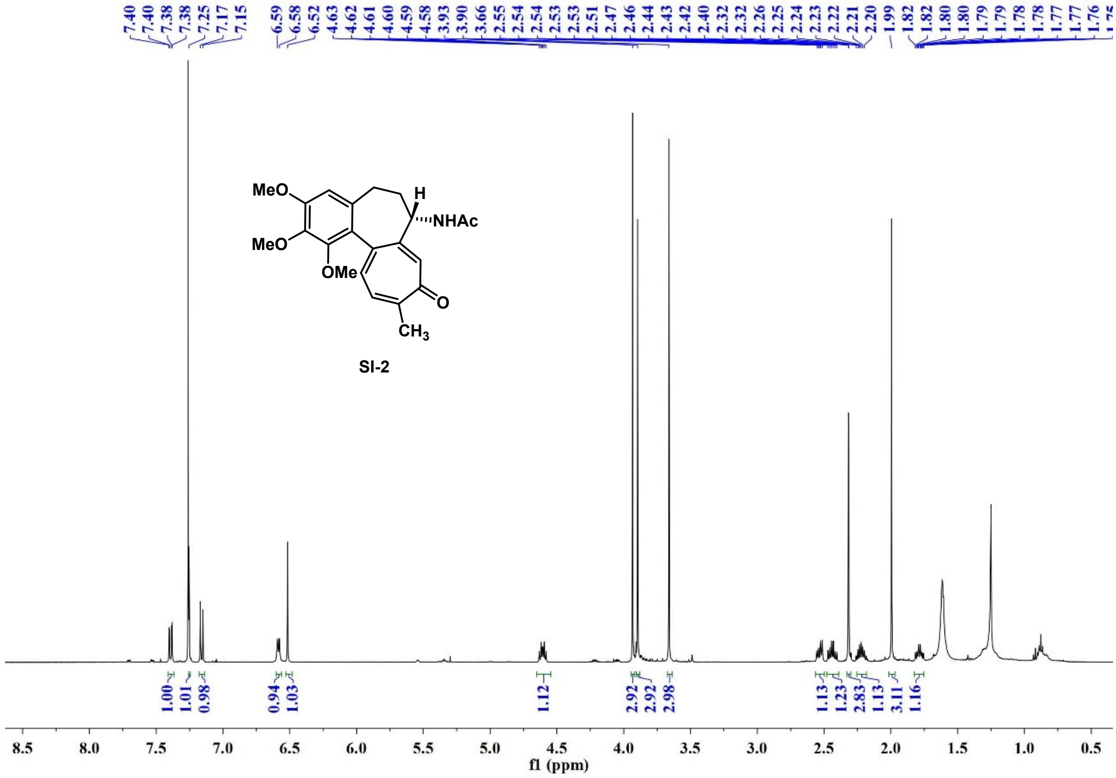
MeO
MeO
OMe
H
NHAc
O
CH3
SI-2
f1 (ppm)

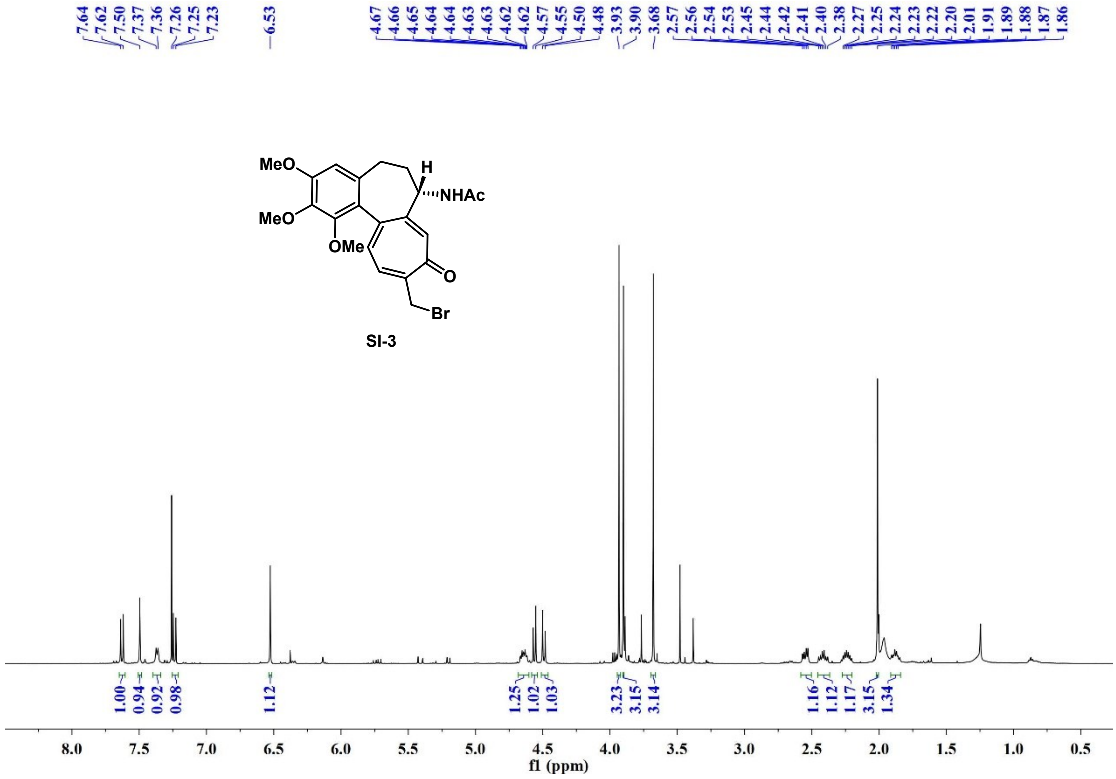
MeO
MeO
OMe
H
NHAc
O
Br
SI-3
f1 (ppm)

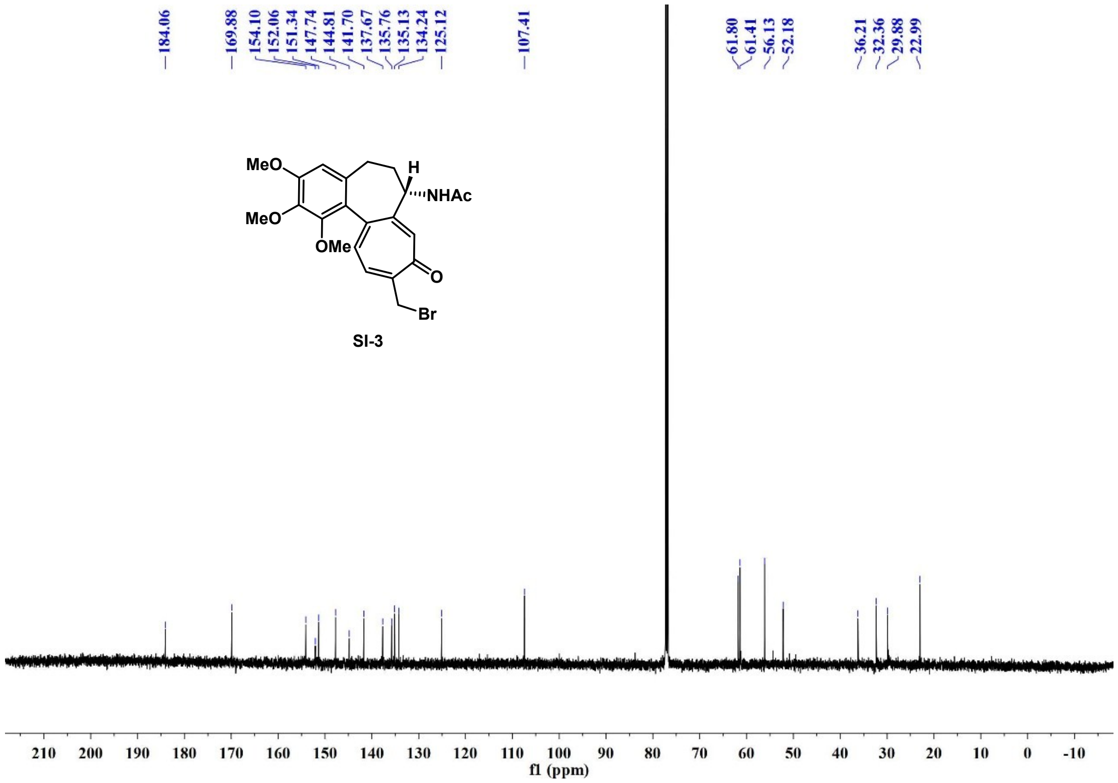

184.06
169.88
154.10
152.06
151.34
147.74
144.81
141.70
137.67
135.76
135.13
134.24
125.12
107.41
61.80
61.41
56.13
52.18
36.21
32.36
29.88
22.99
MeO
MeO
OMe
H
NHAc
O
Br
SI-3
210 200 190 180 170 160 150 140 130 120 110 100 90 80 70 60 50 40 30 20 10 0 -10
f1 (ppm)

VI Biological study

Materials

4', 6-Diamidino-2-phenylindole dihydrochloride (DAPI), 3-(4,5-dimethylthiazol-2-yl)-2,5-diphenyltetrazolium bromide (MTT), propidium iodide (PI) and Ribonuclease A (RNase A) were purchased from Sigma-Aldrich (St. Louis, MO, USA). Fetal bovine serum (FBS), DMEM and penicillin-streptomycin (PS) were supplied by Life Technologies (NY, USA). The anti-β-tubulin antibody was purchased from Cell Signaling Technology (Boston, USA), and Goat anti-Mouse IgG H&L (Chromeo 488) second antibody was obtained from Abcam (Cambridge, UK). Tubulin polymerization assay kit was purchased from Cytoskeleton (Denver, CO, USA).

Cell culture

Human lung carcinoma cell line A549, human breast cancer MDA-MA-231, human colonic carcinoma cell line LoVo were obtained from the American Type Culture Collection (Rockville, MD, USA). All cells were cultured in DMEM supplemented with 10% (*v/v*) fetal bovine serum and 1% (*v/v*) penicillin-streptomycin (Invitrogen) at 37°C under a humidified atmosphere of 5% CO_2.

MTT assay

The cytotoxocities of these synthesized colchicinoids in three human cancer cells were determined by 3-(4,5-cimethylthiazol-2-yl)-2,5-diphenyl tetrazolium bromide (MTT) assay as described previously[12].

Cell cycle analysis

DNA content analysis was performed as described previously[12].

Cell Morphology

Tubulin cytoskeleton organization in MDA-MB-231 cells treated with **30** and colchicine were incubated with anti-β-tubulin antibody and Alexa Fluor 488 secondary antibody. Images were taken using a laser scanning confocal microscope (LSM 510, Zeiss).

Tubulin polymerization assay *in vitro*

Tubulin polymerization assay was performed using tubulin polymerization assay kit according to the manufacturer's instruction.

Microscale thermophoresis binding assay

Binding were calculated for 30 or colchicine and tubulin protein, using microscale thermophoresis[13]. A range of concentrations of the tested componds (30 ranges from 28 μM to 0.05 μM, colchicine ranges from 390 μM to 0.05 μM,) were incubated with tubulin protein marked by fluorescence NT-647 for 5 min in assay buffer. The samples were loaded into the NanoTemper glass capillaries and microthermophoresis (Monolith NT.115). Kd was analyzed based on the mass action equation using the NanoTemper software.

Table S3: Cell growth inhibitory effects of newly synthesized colchicinoids

Compounds	IC_{50} [a] (x ± SD) μM		
	A549	MDA-MB-231	LoVo
**1**	0.0710± 0.0110	0.0332±0.0310	0.0087±0.0023
**2**	>50	>50	>50
**3**	>50	>50	>50
**4**	0.4481 ± 0.1190	0.5752 ± 0.4881	0.4030 ± 0.0647
**22**	0.0276 ± 0.0106	0.0310 ± 0.0047	0.0346 ± 0.0032
23	**0.0028 ± 0.0009**	**0.0032 ± 0.0003**	**0.0035 ± 0.0006**
**24**	3.6120 ± 1.3900	0.0329 ± 0033	0.1466 ± 0.1936
**26**	34.6400 ± 4.2400	33.1878 ± 4.5039	46.2686 ± 0.6266
**27**	>50	>50	>50
**28**	0.0314 ± 0.0066	0.0033 ± 0.0004	0.0042 ± 0.0008
**30**	0.0637 ± 0.0159	0.1316 ± 0.1629	0.0327 ± 0.0032
**31**	0.0562 ± 0.0011	0.0322 ± 0.0056	0.0304 ± 0.0052
**32**	16.0900 ± 3.6100	36.0588 ± 2.7287	35.9833 ± 6.3822

[a] IC_{50} values are expressed as the mean values **±** S.D. from three independent experiments

Figure S1. Inhibition of the polymerization of tubulin by 23 and direct binding of 23 to tubulin *in vitro*.

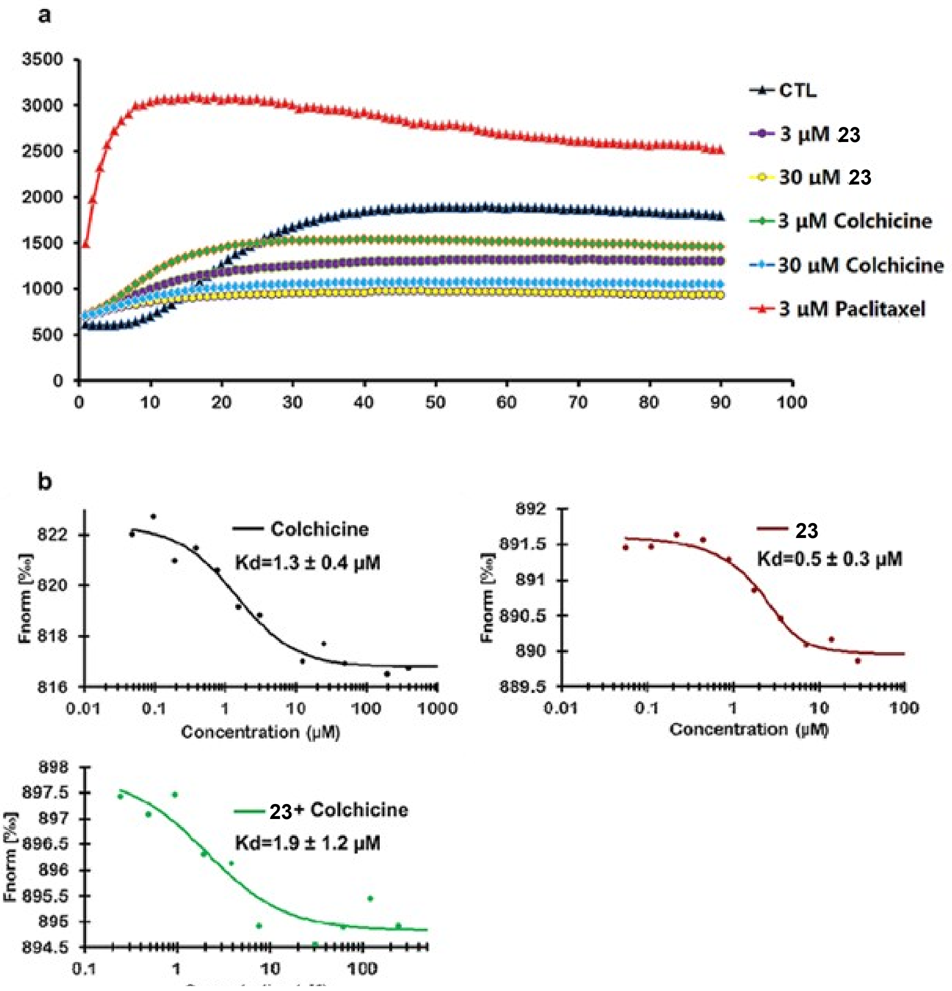

a, Effects of **23** (3 µM and 30 µM), colchicine (**1**, 3 µM and 30 µM) and paclitaxel (3 µM) on the polymerization of tubulin. Absorbance readings were recorded using an Infinite® F500 Multimodel Plate Reader (TECAN, Austria) at 1 min intervals for 90 min at 37 °C. b, Binding isotherms for **23** and colchicine (**1**) to tubulin, directly measured by microscale thermophoresis based on changes in the signal. Black line, colchicine (**1**); red line, **23**; green line, tubulin pretreated with **23** for 0.5 h before treatment with colchicine (**1**). These data are representative of three independent experiments, and the error bars indicate the standard deviation.

Figure S2. Disruption of the microtubule structure and induction of G_2/M cell cycle arrest in MDA-MB-231 cells by 23.

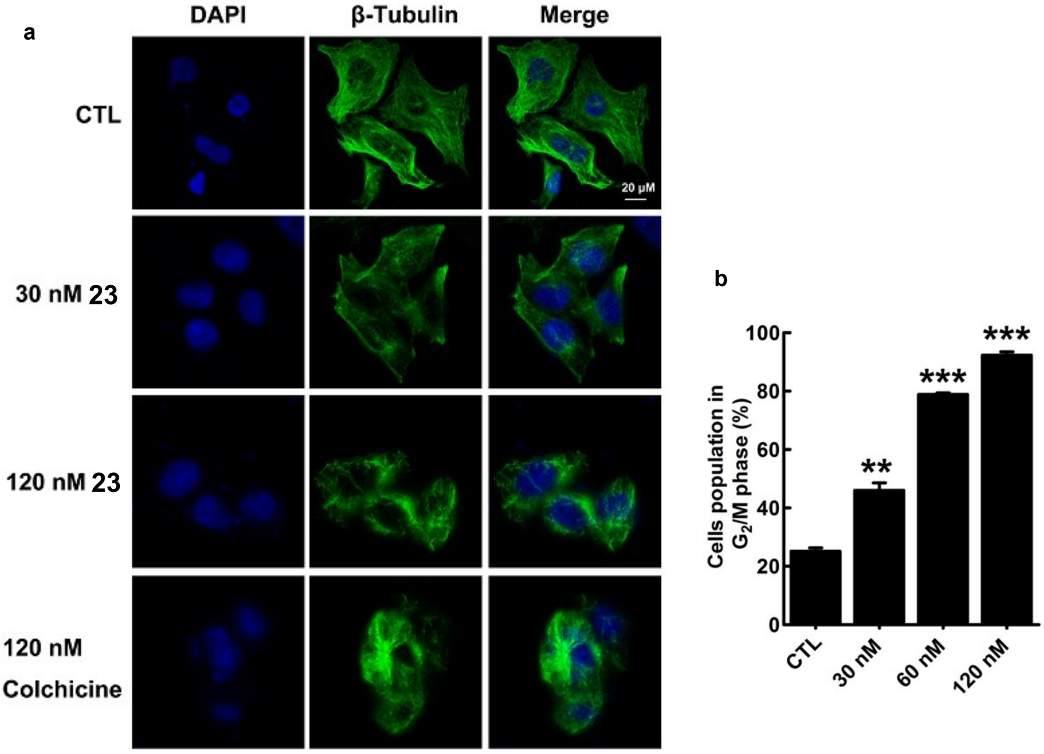

a, Effect of **23** on the organization of the microtubule network in MDA-MB-231 cells. Cells were treated with **23** for 12 h. Cells were also treated with colchicine (**1**) as a positive control under the same conditions. Original magnification: 630×; Scale bar: 20 μm. b, Effect of **23** on cell cycle distribution in MDA-MB-231 cells. Cells were incubated with **23** (30, 60 and 120 nM) for 24 h, fixed, stained with PI and RNase A, and analysed by flow cytometry (Millipore, Guava easyCyte™). These data are expressed as the mean values ± S.D. derived from three separate cultures ($p \leq 0.05$).

Table S4: A summary of colchicine syntheses.[16]

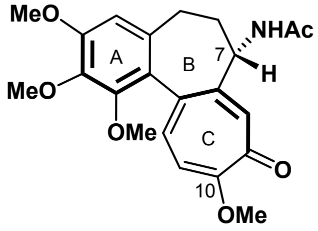

(–)-(a*R*,7*S*)-colchicine (1)

Synthetic challenges:

- **Unusual 6-7-7 membered ring system**
- **Regioselective construction of the tropolone C-ring**
- **Stereoselective synthesis of the stereogenic axis**
- **Enantioselective installation of the C-7-acetamido group**

Target	Group	Starting compound	Key Reactions	Steps (Yield)
(±)-colchicine (**1**)	Eschenmoser (1959)[17]	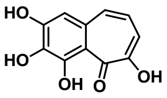	Ring expansion	22 (0.00006%)
(–)-colchicine (1)	Banwell (1996)[18]	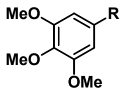 **5a: R = CHO**	Cationic cyclization/ Ring expansion	15 (0. 9%)
	Cha (1998)[19]	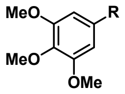 **5b: R = $(CH_2)_3OH$**	[4+3] cycloaddition	18 (1.9%)
	Schmalz (2005)[20]	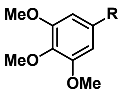 **5c: R = $(CH_2)_2COOH$**	Rh-Triggered [3+2] Cycloaddition Cascade	17 (0.8%)
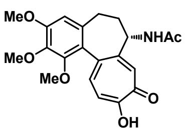 (±)-colchiceine	Nakamura (1962)[21]	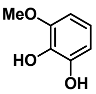	Decarboxylative Dieckmann condensation	20 (0.007%)
	Woodward (1963)[22]	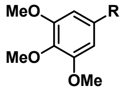 **5a: R = CHO**	Friedel–Crafts alkylation/Dieckmann condensation	23 (unreported)
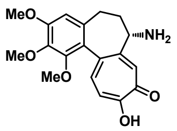 (±)-trimethylcolchicinic acid	Van Tamelen (1959)[23]	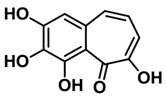	Acyloin condensation	18 (0.0005%)
	Evans (1984)[24]	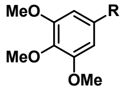 **5a: R = CHO**	Friedel–Crafts cyclization/ Ring expansion and rearrangement	12 (5%)
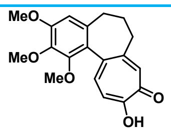 (±)-desacetamidocolchiceine	Scott (1965)[25]	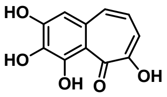	Oxidative Coupling	11 (0.1%)

	Toromanoff (1965)[26]	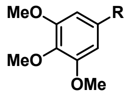 5d: R = $(CH_2)_3Cl$	Friedel–Crafts/ Dieckmann condensation	15 (0.3%)
	Kaneko (1968)[27]	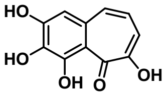	Oxidative Coupling	11 (0.5%)
	Kato (1974)[28]	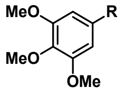 5e: R = $(CH)_2CO_2Me$	Oxidative Coupling	9 (1%)
	Boger (1985)[29]	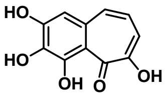	[4+3] Cycloaddition	10 (3.5%)
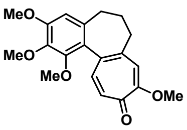	Tobinaga (1974)[30]	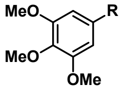 5a: R = CHO	Electrochemical oxidative cyclization /Ring expansion and rearrangement	7 (24%)
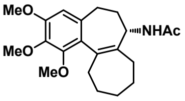	Wenkert (1989)[31]	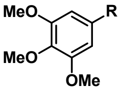 5c: R = $(CH_2)_2COOH$	[3,3]-Sigmatropic rearrangement/ Dieckmann condensation	10 (6.3%)
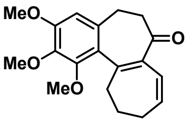	Hanna (2007)[32]	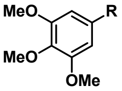 5c: R = $(CH_2)_2COOH$	Tandem RCM reaction	12 (1.3%)

Table S5: A summary of the introduction of C-7-amino chiral center.

<table>
<tr><th>Group</th><th>Year</th><th>Key reactions</th></tr>
<tr><td>Banwell</td><td>1996</td><td>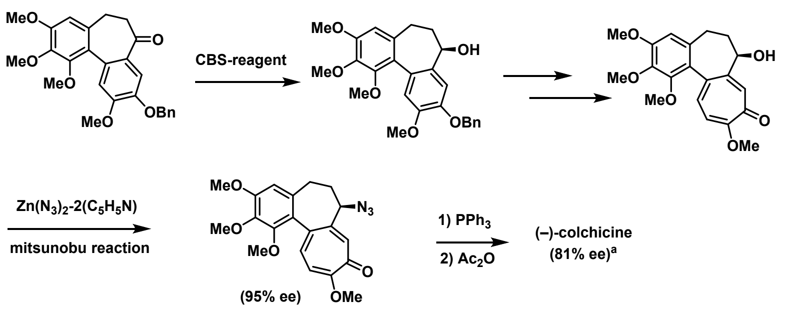
</td></tr>
<tr><td>Cha</td><td>1998</td><td>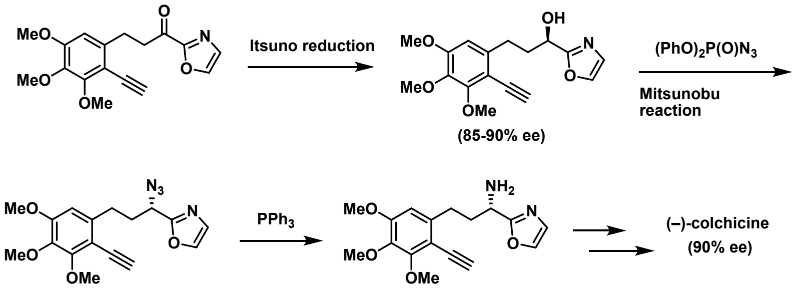
</td></tr>
<tr><td>Schmalz</td><td>2006</td><td>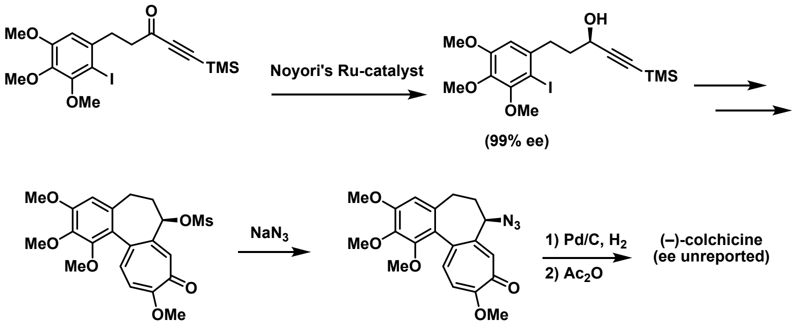
</td></tr>
<tr><td>Our group</td><td>2017</td><td>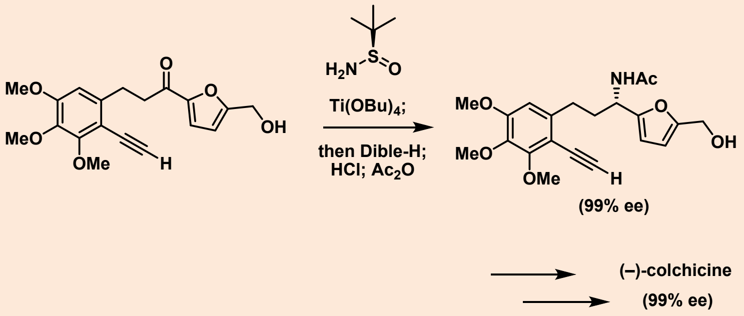
</td></tr>
</table>

[a] "The partial racemisation observed in the final stages of this synthesis is attributed to the substantial acidity of the C-7 proton associated with the intermediate phosphoimine involved in the azide reduction step. "[18]

References

1 H. Hagiwara, Y. Sugawara, K. Isobe, T. Hoshi and T. Suzuki, *Org. Lett.* 2004, **6**, 2325.

2 V. Rys, A. Couture, E. Deniau and P. Grandclaudon, *Eur. J. Org. Chem.* 2003, 1231.

3 J. Carreras, G. Gopakumar, L. G. Gu, A. Gimeno, P. Linowski, J. Petuskova and W. Thiel, M. Alcarazo, *J. Am. Chem. Soc.* 2013, **135**, 18815.

4 D. K. Nielsen, L. L. Nielsen, S. B. Jones, L. Toll, M. C. Asplund and S. L. Castle, *J. Org. Chem.* 2009, **74**, 1187.

5 K. Padala and M. Jeganmohan, *Org. Lett*. 2012, **14**, 1134.

6 H. G. Capraro and A. Brossi, *Helv. Chim. Acta.* 1979, **62**, 965.

7 R. F. Raffauf, A. L. Farren, and G. E. Ullyot, *J. Am. Chem. Soc.* 1953, **75**, 3854.

8 F. Q. Alali, T. El-Elimat, C. Li, A. Qandil, A. Alkofahi, K. Tawaha, J. P. Burgess, Y. Nakanishi, D. J. Kroll, H. A. Navarro, J. O. Falkinham, M. C. Wani and N. H. Oberlies, *J. Nat. Prod.* 2005, **68**, 173.

9 G. W. A. Mine, *Drugs: Synonyms & Properties*. 2000.

10 H. Potesilova, J. Jr. Hrbek and F. Santavy, *Collect. Czech. Chem. Commun.* 1967, **32**, 141.

11 R. Dumont, A. Brossi and J. V. Silverton, *J. Org. Chem*. 1986, **51**, 2515.

12 J. Zheng, L. Deng, M. F. Chen, X. Z. Xiao, S. W. Xiao, C. P. Guo, G. K. Xiao, L. L. Bai, W.C. Ye, D. M. Zhang and H. R. Chen, *Eur.J.Med.Chem.* 2013, **65**, 158-167.

13 J. L. Parker and S. Newstead, *Nature*, 2014, **507**, 68.

14 M. A. Jordan and L. Wilson, *Nat. Rev. Cancer* 2004, **4**, 253.

15 F. Pellegrini and D. R. Budman, Cancer Invest. 2005, **23**, 264.

16 T. Graening and H.G. Schmalz, *Angew. Chem. Int. Ed.* 2004, **43**, 3230

17 (a) J. Schreiber, W. Leimgruber, M. Pesaro, P. Schudel and A. Eschenmoser, *Angew. Chem*. 1959, **71**, 637; (b) J. Schreiber, W. Leimgruber, M. Pesaro, P. Schudel and A. Eschenmoser, *Helv. Chim. Acta* 1961, **44**, 540.

18 M. G. Banwell, *Pure Appl. Chem.* 1996, **68**, 539.

19 (a) J. C. Lee, S. J. Jin and J. Cha, *J. Org. Chem*. 1998, **63**, 2804; (b) J. C. Lee, S. J. Jin and J. Cha, *Tetrahedron* 2000, **56**, 10175.

20 (a) T. Graening, W. Friedrichsen, J. Lex and H. G. Schmalz, *Angew. Chem*. 2002, **114**, 1594; *Angew. Chem. Int. Ed*. 2002, **41**, 1524; (b) T. Graening, V. Bette, J. Neudorfl, J. Lex and H. G. Schmalz, *Org. Lett*. 2005, **7**, 4317.

21 (a) T. Nakamura, Y. Murase, R. Hayashi and Y. Endo, *Chem. Pharm. Bull*. 1962, **10,** 281; (b) G. Sunugawa, T. Nakamura and J. Nakazawa, *Chem. Pharm. Bull.* 1962, **10**, 291; (c) T. Nakamura, *Chem. Pharm. Bull*. 1962, **10**, 299.

22 R. B. Woodward, The Harvey Lecture Series 1963, **59**, 31.

23 (a) E. E. van Tamelen, T. A. Spencer, Jr., D. S. Allen, and Jr., R. L. Ovis, *J. Am. Chem. Soc.* 1959, **81**, 6341; (b) E. E. van Tamelen, T. A. Spencer, Jr., D. S. Allen, and Jr., R. L. Ovis, *Tetrahedron* 1961, 8.

24 (a) D. A. Evans, D. J. Hart and P. M. Koelsch, *J. Am. Chem. Soc*. 1978, **100**, 4593; (b) D. A. Evans, S. P. Tanis and D. J. Hart, *J. Am. Chem. Soc*. 1981, **103**, 5813.

25 A. I. Scott, F. McCapra, R. L. Buchanan, A. C. Day and D. W. Young, *Tetrahedron* 1965, **21**, 3605.

26 J. Martel, E. Toromanoff and C. Huynh, *J. Org. Chem*. 1965, **30**, 1752.

27 S. I. Kaneko and M. Matsui, *Agric. Biol. Chem.* 1968, **32**, 995.

28 M. Kato, F. Kido, M. D. Wu and A. Yoshikoshi, *Bull. Chem. Soc. Jpn*. 1974, **47**, 1516.

29 (a) D. L. Boger and C. E. Brotherton, *J. Org. Chem*. 1985, **50**, 3425; (b) D. L. Boger and C. E. Brotherton, *J. Am. Chem. Soc*. 1986, **108**, 6713.

30 E. Kotani, F. Miyazaki and S. Tobinaga, *J. Chem. Soc. Chem. Commun*. 1974, 300.

31 E. Wenkert and H. S. Kim in *Studies in Natural Products Chemistry (Part B), Vol. 3* (Ed.: Atta-ur-Rahman), Elsevier, Amsterdam, 1989, p. 287.

32 F. D. Boyer and I. Hanna, *Org. Lett*. 2007, **9**, 2293.
